# Poly(bis-arylimidazoliums) possessing high hydroxide ion exchange capacity and high alkaline stability

**DOI:** 10.1038/s41467-019-10292-z

**Published:** 2019-05-24

**Authors:** Jiantao Fan, Sapir Willdorf-Cohen, Eric M. Schibli, Zoe Paula, Wei Li, Thomas J. G. Skalski, Ania Tersakian Sergeenko, Amelia Hohenadel, Barbara J. Frisken, Emanuele Magliocca, William E. Mustain, Charles E. Diesendruck, Dario R. Dekel, Steven Holdcroft

**Affiliations:** 1 Department of Chemistry, Simon Fraser University, 8888 University Drive, Burnaby, BC V5A 1S6 Canada; 2 The Wolfson Department of Chemical Engineering, Technion-Israel Institute of Technology, Haifa, 3200003 Israel; 3 Department of Physics, Simon Fraser University, 8888 University Drive, Burnaby, BC V5A 1S6 Canada; 4 Department of Chemical and Biomolecular Engineering, University of Connecticut, Storrs, Connecticut 06269 USA; 5 Schulich Faculty of Chemistry, Technion-Israel Institute of Technology, Haifa, 3200008 Israel

**Keywords:** Fuel cells, Polymer synthesis, Fuel cells, Polymers

## Abstract

Solid polymer electrolyte electrochemical energy conversion devices that operate under highly alkaline conditions afford faster reaction kinetics and the deployment of inexpensive electrocatalysts compared with their acidic counterparts. The hydroxide anion exchange polymer is a key component of any solid polymer electrolyte device that operates under alkaline conditions. However, durable hydroxide-conducting polymer electrolytes in highly caustic media have proved elusive, because polymers bearing cations are inherently unstable under highly caustic conditions. Here we report a systematic investigation of novel arylimidazolium and bis-arylimidazolium compounds that lead to the rationale design of robust, sterically protected poly(arylimidazolium) hydroxide anion exchange polymers that possess a combination of high ion-exchange capacity and exceptional stability.

## Abstract (web summary)

The hydroxide anion exchange polymers are key materials of solid polymer electrolyte devices that operate under alkaline conditions. Here the authors show the rational design of such polymers that exhibit high ion exchange capacity and exceptional stability under highly caustic conditions.

## Introduction

Alkaline anion-exchange membranes (AAEMs) have gained attention due to their potential integration into a wide range of electrochemical energy storage and conversion technologies^1–14^. Anion-conducting polymers possess immobilized cationic groups that promote ion conduction but these same cations are the weak link in caustic media^15–19^. We have previously reported^20^ that the *o*-dimethylphenyl, C2-sterically protected benzimidazolium cation is more stable in basic environments than the phenyl-linked benzimidazolium analog due to enhanced steric hindrance around the C2-benzimidazolium position, which serves to inhibit ring-opening degradation. *o*-Dimethylphenyl-protected benzimidazolium polymer analogs are stable in 2 M KOH at 60 °C for extended periods^20–22^. Futhermore, Coates et al.^23^ reported enhanced alkaline stability of *o*-dimethylphenyl-protected imidazolium cations, and Fan et al.^24^ explored sterically protected polymeric analogs, reporting a poly(arylimidazolium) cationic polyelectrolyte that exhibited prolonged stability in 10 M KOH at elevated temperatures. Studies of the *o*-dimethylphenyl imidazolium model compound indicated that ring-opening degradation was greatly suppressed to the extent that the next available mode of degradation, dealkylation, became prominent. Investigating degradation pathways of poly(arylimidazoliums) under aggressive, caustic conditions, minimizing the dealkylation degradation pathway, while maintaining a high ion-exchange capacity (IEC) to promote anion conduction, is the focus in this paper. Developing an effective synthetic route for higher molecular weight poly(arylimidazoliums) in order to enhance their dimensional stability for the purpose of fabricating robust membranes is a second focus.

Based on density functional theory (DFT) calculations^24^, the computed free energies for degradation via ring-opening increases with the size of the protecting group around the C2-imidazolium position. However, among phenyl, *o*-dimethylphenyl, and *o*-diphenylphenyl C2-protected imidazoliums, the free energies for degradation via demethylation are similar, which indicates the sterically protecting groups around the C2 position exert negligible effect on this decomposition pathway. Bulky substituents attached to the N1/N3-imidazolium position may exert a stabilizing effect on the imidazolium cations. Coates et al.^23^ have shown that increasing the length of an alkyl chain at one of these positions enhances the stability of the imidazolium cation. Long et al.^25^ reported that for *n*-alkyltrimethyl ammonium cations the energy barrier for degradation via dealkylation increases from methyl to butyl.

In this work, various imidazolium and (bis)imidazolium cations are synthesized by alkylating corresponding imidazoles with different alkyl side chains and their stability to caustic conditions evaluated. The results are used to down select optimal cationic functionalities for the synthesis of poly(arylimidazolium) analogs that lead to robust, sterically protected poly(arylimidazolium) hydroxide anion exchange membranes that possess high IEC and exceptional stability under highly caustic conditions.

## Results

### Suppressing dealkylation of methylated imidazolium

**Fig. 1:**
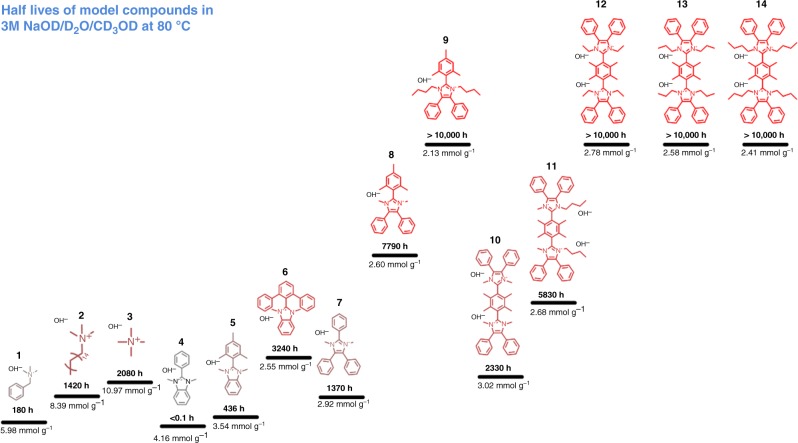
Half-lives of cationic groups in 3 M NaOD/D_2_O/CD_3_OD. Half-lives of six novel imidazolium cations **9**–**14** prepared for this work, along with ammonium cations^24^ **1**–**3**, and previously published benzimidazolium cations^27^ **4**–**6** and imidazolium cations^24^ **7**–**8**. All model compounds were studied as 0.02 M solutions in 3 M NaOD containing 7:3 wt. CD_3_OD:D_2_O at 80 °C. The water to ion ratio (hydration level), *λ*, is 4.8. Half-lives (*t*_1/2_) of compounds **9–14** are estimated from data reported in Supplementary Figs. 1–12. The hydroxide ion-exchange capacity is provided

**Fig. 2:**
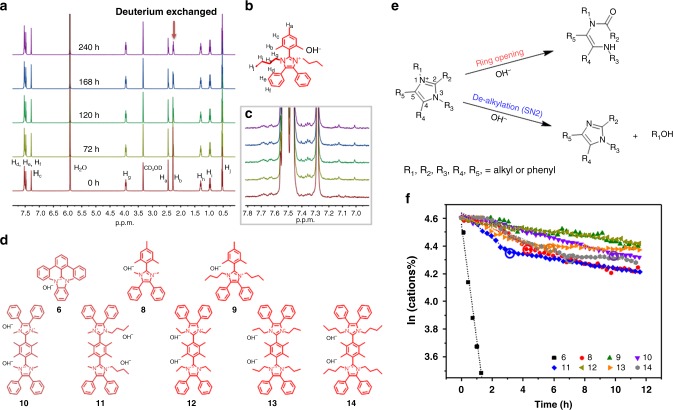
Degradation of imidazolium. **a**, **b**, **c** Selected regions of the ^1^H NMR spectra of **9** (0.02 M) in 3 M NaOD/CD_3_OD/D_2_O (7:3 wt. CD_3_OD:D_2_O) after being heated at 80 °C as a function of time. **d**, Compounds structure corresponding to plot in Fig. 2f. **e**, Degradation pathways of imidazolium cation. **f** Remaining percentage of model compounds **6** and **8**–**14** as a function of time at *λ* = 1 and 0.5 M OH^−^ at room temperature. Circled points represent break points, see text for details. NMR-determined stability data of novel compound **9**–**14** are provided in Supplementary Figs. 1–12

Six imidazolium model compounds **9**–**14** possessing steric-protecting groups at the C2 position and different alkyl side chains attached to the N1/N3 imidazole position were synthesized. The half-life of these in 3 M NaOD at 80 °C are shown in Fig. 1, together with the half-life of previously reported ammonium cations and benzimidazolium cations, shown for comparison. Compound **1**, the most widely studied quaternary ammonium (QA) cation, degraded to 50% of its original amount within 180 h via demethylation and debenzylation. In the absence of the phenyl ring, compounds **2** and **3** are substantially longer-lived (half-lives of 1420 and 2080 h, respectively). Hibbs^26^ reported membranes comprising a poly(phenylene) backbone with compounds **1** and **2** attached as pendent cationic groups that exhibit 33% and 5% loss in conductivity over 14 days in 4 M KOH at 90 °C, respectively. Our previous studies of benzimidazoliums cations **4**, **5**, and **6** demonstrated increasing stability of the C2 substituent: phenyl < *o*-dimethylphenyl < *o*-diphenylphenyl (half-lives of <0.1, 436 and 3240 h, respectively)^27^. The stability of the corresponding three polymers showed a similar trend in stability, i.e., rapid degradation (minutes) in 0.3 M KOH at 25 °C^20^, 8% degradation after 168 h in 1 M KOH at 80 °C^22^, and 5% degradation after 168 h in 2 M KOH at 80 °C^27^, respectively. The major degradation pathway, illustrated in Fig. 2e, evolved from ring-opening for **4** and **5** to both ring-opening and demethylation for **6**, and is supported by DFT calculations, which show that increased steric crowding at the C2 position raises the energy barrier for ring-opening degradation. Imidazolium cations **7** and **8** exhibit enhanced stability compared with benzimidazolium cations **4** and **5**. Compound **7** degraded by both ring-opening and dealkylation, whereas compound **8** degraded solely by the dealkylation pathway^24^.

**Fig. 3:**
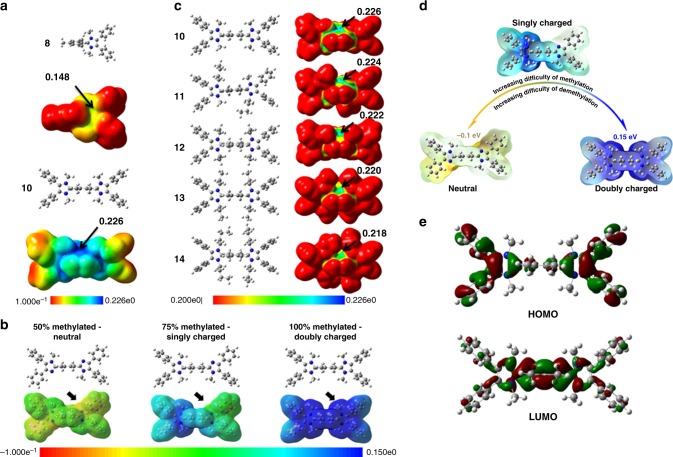
DFT analyses of imidazoliums. **a** Electrostatic potentials (ESP) of **8** and **10** (potential bar 0.1 to 0.226 eV); **b** dimethylated (neutral), trimethylated (singly charged), and tetramethylated (doubly charged) imidazole (or imidazolium) (potential bar −0.1 to 0.15 eV underneath); **c** ESP of **10**–**14** (potential bar 0.2–0.226 eV). **d** Methylated or demethylated scheme of compound **10**. **e** HOMO and LUMO orbitals of **10**

Given these correlations, we focused efforts on suppressing dealkylation of imidazolium by utilizing longer alkyl side chains attached to the N1/N3 position. Compound **9**, with its longer alkyl chains, was found to be significantly more stable (*t*_1/2_ > 10,000 h) than **8** (dimethyl). No degradation of **9** was observed by nuclear magnetic resonance (NMR) spectroscopy after 10 days (Fig. 2a–d), which not only supports that dealkylation has been suppressed but also indicates that Hoffman elimination (potentially due to the presence of β-hydrogens) is absent. However, a consequence of attaching longer chains at the *N*-position is that the molecular weight of the repeat unit increases and hence the charge per mass ratio of the molecule decreases, which may adversely affect ionic conductivity of membranes prepared therefrom. A consequence of introducing two *n*-butyl groups at the expense of two methyl groups, e.g., is that the IEC of **9** and thus the maximum IEC of polymers prepared form **9** is limited to 2.13 mmol g^−1^ (reduced from 2.6 mmol g^−1^, dimethyl). To counter this, we prepared bis-imidazolium cations **10–14** for which the charge per mass is higher, as indicated in Fig. 1. The IEC of bis-imidazolium cations **10**–**14** are in the range 3.02–2.41 mmol g^−1^ compared with 2.60–2.13 mmol g^−1^ for imidazoliums **8** and **9**. However, it is found that the proximity of the second imidazolium cation in **10** lowers its stability compared with the analogous single imidazolium cation **8**. This can be explained by the higher electrostatic potential (ESP), illustrated in Fig. 3a. The ESP around the imidazolium cation **10** is 0.226 eV, compared with 0.148 eV for compound **8**, which suggests that the electrostatic attraction between the cation and hydroxide anion is stronger, and may lead to more rapid attack of hydroxide on the imidazolium. A difference in the dihedral angles between the imidazolium and C2-phenyl might also play a role^20^; however, the dihedral angles of *o*-dimethylphenyl and imidazolium ring for **10** and **8** are similar (82° vs. 79°) so this explanation may be ruled out. Indirect experimental evidence that a second imidazolium placed in close proximity increases the localized positive charge density of the molecule is drawn from the observation that complete alkylation (100% methylation) of the precursor of compound **10** to form the doubly charged molecule is synthetically much more difficult than alkylation to form the singly charged species (i.e., 75 % alkylation). For example, 75% alkylation of the precursor to compound **10** (i.e., single cation formation) can be accomplished at room temperature but further alkylation to the double cation requires much higher temperatures and pressurized conditions. This observation is also supported by ESP calculations where it can be shown that the positive charge of one imidazolium lowers the electron density and nucleophilicity of the nitrogen on the adjoining imidazole (Fig. 3b). Here, the neutral precursor of **10** (50% methylated) possesses a lower ESP than the non-methylated N– for the singly charged version of **10** (75% methylated), indicating that one positive charge on compound **10** renders the remaining non-methylated N a weaker nucleophile for the final alkylation reaction (Fig. 3d). Furthermore, DFT calculations predict that upon dealkylation of one of the alkyl groups in **10**, the remaining imidazolium is further stabilized. Despite activation toward mono-dealkylation of **10**, caused by formation of the second cationic charge, compound **10** exhibits a remarkable half-life of >2000 h under aggressive, caustic alkaline conditions. Attaching longer alkyl chains, with their stronger electron-donating effect on the imidazolium ring, reduces the DFT-calculated ESP values, as shown in Fig. 3c. Moreover, longer alkyl chains confer increased steric hindrance around the alkyl-N bond. Thus, **12**–**14** exhibit exceptional stabilities of >10,000 h (Fig. 1) in 3 M NaOD/D_2_O/CD_3_OD. For completion, NMR-determined stability data of compounds **9**–**14** are shown in Supplementary Figs. 1–12.

### Stability under *λ* = 1 conditions

Given that the stability of several bis-imidazoliums in 3 M NaOD/D_2_O/CD_3_OD were indistinguishably high, harsher conditions were examined to probe their relative stability. Recently, Dekel et al.^28–31^ reported that very low hydration levels accelerate degradation of cationic groups in alkaline media, and that hydration levels of *λ* < 5 increases the rate of degradation of cationic groups by up to several orders of magnitude. Model compounds **1–14** were therefore examined under minimal hydration, i.e., *λ* = 1, using ultra-dry KOH (0.5 M in dimethyl sulfoxide (DMSO)/crown ether) according to the procedure outlined in ref. ^32^. The evolution of NMR spectra of the degradation of model compounds is shown in Supplementary Figs. 13–24 and the rate of degradation is plotted in Fig. 2f and tabulated in Supplementary Table 1. As anticipated, all compounds exhibited orders of magnitude lower stability under these hasher conditions. Interestingly, it was observed that Hoffmann degradation of the alkane side chains is the dominant decomposition pathway under these conditions, as opposed to dealkylation as was observed in 3 M NaOD/D_2_O/CD_3_OD. These results are consistent with the DFT calculations carried out for *λ* = 0 condition^25^, which provides evidence that the preferential degradation pathway does not solely depend on the structure of cationic groups; hydration level also plays an important role. Under these ultra-low hydration levels, **1** and **3** exhibit the highest chemical stability with half-lives >180 and 600 h, respectively, due to the absence of β-hydrogens; nucleophilic attack by the hydroxide ion is dominant in this environment^30,32^. However, although the 1 and 3 are stable in the *λ* = 1 condition, when they are attached to a polymer, they are not stable in the actual operation, such as fuel cell (FC) and water electrolysis, as the *λ* level is generally higher in these devices. The trends of stability of the (benz)imidazoliums under these conditions are consistent in trend with their stability in 3 M NaOD/D_2_O/CD_3_OD, where compounds **6**, **8**, and **9** exhibit increasing stability. The least stable model compounds under low hydration conditions were **2**, **4**, **5**, and **7**, which decomposed immediately to the extent that degradation plots were unobtainable. Bis-imidazolium compounds **10**, **11**, **13**, and **14** exhibited a two-stage rate of degradation, which is also consistent with the calculations illustrated in Fig. 3b–d, which indicate that after the loss of one N-alkyl group (and hence loss of the first cationic charge), the remaining cationic group is less susceptible to degradation. The calculated highest occupied molecular orbital and lowest unoccupied molecular orbital orbit of **10** provide additional evidence that the positive charge is distributed over the N1/C2/N3 atoms of the imidazole ring and also explains why deuterium exchange may occur at protons associated on the tetramethylphenyl group (Fig. 2a).

### Poly(arylimidazoliums)

**Fig. 4:**
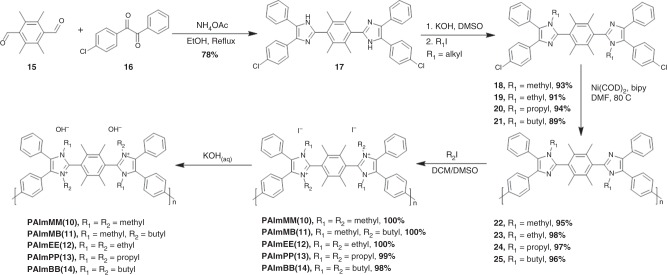
Synthesis of poly(arylimidazoliums) PAImXY(#). X and Y represent alkyl chains and **#** represents the analogous model compound illustrated in Fig. 1. Five polymers were synthesized: PAImMM(**10**), PAImMB(**11**), PAImEE(**12**), PAImPP(**13**), and PAImBB(**14**), possessing the same architecture as compounds **10**–**14**, where M = methyl, E = ethyl, P = propyl, and B = butyl

As compounds **12–14** demonstrated exceptional chemical stability in both 3 M NaOD/D_2_O/CD_3_OD (*λ* = 4.8) and 0.5 M KOH/DMSO/crown ether (*λ* = 1), analogous polymers with the same molecular architecture were designed and synthesized. Novel, sterically protected poly(arylimidazoliums) were prepared by Yamamoto-coupling homo-polymerization of dichloro-imidazole monomers as shown in Fig. 4. This synthetic route is substantially different to that previously reported for a poly(arylimidazoliums), which was based on a bis-diketone/dialdehyde/ammonium polycondensation^24^.The newly devised route is an important advancement, as it mitigates issues of solubility during polymerization that limit the molecular weight of the poly(arylimidazoliums).

**Fig. 5:**
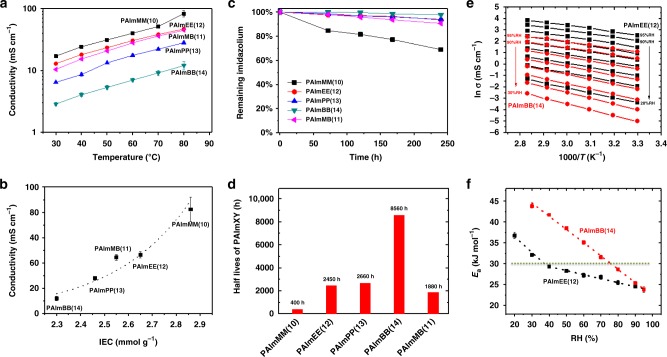
Properties of PAImXY(#). **a** Ionic conductivity of PAImXY(**#**) (Cl^−^). Temperature-dependent conductivity under 95% RH. **b** Ionic conductivity at 80 °C, 95 % RH vs. IEC of PAImXY(**#**). **c** Stability of PAImXY(**#**) to caustic solution after immersion in 10 M KOH at 80 °C for 240 h. **d** Calculated half-life. **e** Arrhenius plots of ion conductivity of PAImEE(**12**) (black) and PAImBB(**14**) (red) in chloride form at various temperature and RH in air. **f** The corresponding calculated activation energy at a given RH

Biaryl couplings in a polymer generally lead to low-molecular-weight polymer, because highly rigid aromatic backbones reduce their solubility. However, the synthetic strategy adopted allowed preparation of an intermediary polymer, in its semi-alkylated form, which afforded much higher solubility, higher high-molecular-weight poly(arylimidazoles) (Mw = 140 kDa, Polydispersity Index = 1.70), as shown in Supplementary Fig. 25. N-alkylation of the polymers was achieved by reacting the polymer with different alkylating reagents in dichloromethane/DMSO at 80 °C in a pressure reactor for 3 days, to produce various poly(arylimidazoliums) in their iodide form. Transparent, colorless, flexible, and tough membranes were obtained after casting and washing with 1 M KOH (Supplementary Fig. 26, Table 1). Membranes were converted to the chloride form and characterized. As shown in Table 1, PAImMM(**10**)Cl^−^ exhibits an anion conductivity of 33 mS cm^−1^ for a hydration level of 9. The swelling of PAImMM(**10**) Cl^−^ in water is 45 wt%, which is low given the membrane’s high IEC, 2.86 mmol g^−1^. Polymers prepared with longer alkyl side chains correspondingly possess lower IEC^33,34^. The swelling of PAImEE(**12**), PAImPP(**13**), and PAImBB(**14**) decreases from 26.1 % to 20.3%, to 14.0%, with calculated *λ*-values of 6, 5, and 3, respectively. Compared with PAImEE(**12**), the unsymmetrically alkylated polymer PAImMB(**11**) has a higher water uptake and hydration level, although it possesses a lower IEC and swelling ratio than PAImEE(**12**). Maximum conductivities of 82 and 12 mS cm^−1^ were observed at 80 °C under 95% relative humidity (RH) for PAImMM(**10**) and PAImBB(**14**) (Fig. 5a), respectively. The large difference in conductivity is due to the 0.5 mmol g^−1^ difference in IEC and subsequent decrease in water content. A clear relationship between conductivity and IEC can be observed from Fig. 5b.

**Table 1:** Properties of PAImXY(#) membranes

	Water uptake (wt %)	Swelling (wt%)	IEC_Cl_^a^ (mmol g^−1^)	*λ* ^b^	*σ*^c^ (mS cm^−1^)	TS^d^ (MPa)	E@B^e^ (%)	Modulus^f^ (MPa)
PAImMM(**10**)	48.2 ± 1.3	45.0 ± 2.3	2.86	9	32.7 ± 2.4	75.0 ± 3.9	17.4 ± 2.7	1549 ± 148
PAImMB(**11**)	30.0 ± 2.0	24.2 ± 1.5	2.55	7	15.9 ± 0.8	67.0 ± 3.3	23.7 ± 3.4	1040 ± 112
PAImEE(**12**)	28.1 ± 1.6	26.1 ± 4.0	2.65	6	21.3 ± 1.6	64.0 ± 11.6	28.7 ± 8.0	1075 ± 212
PAImPP(**13**)	22.3 ± 1.3	20.3 ± 2.0	2.46	5	14.8 ± 1.2	57.2 ± 2.0	19.5 ± 1.2	1292 ± 46
PAImBB(**14**)	12.2 ± 2.0	14.0 ± 1.2	2.30	3	8.5 ± 0.7	65.2 ± 5.3	20.4 ± 1.8	1095 ± 54

Water uptake and swelling ratio of PAImXY(**#**)measured in the chloride form. ^a^Theoretical ion-exchange capacity of PAImXY(**#**). ^b^Hydration level, [H_2_O]/[Cl^−^] molar ratio. ^c^Ionic conductivity of PAImXY(**#**) in chloride form at 22 °C in water. ^d^TS tensile strength. ^e^E@B elongation at break. ^f^Young’s modulus

Chemical stability to caustic solutions was examined by calculating the percentage of the imidazolium remaining in the polymer after immersion in 10 M KOH at 80 °C as a function of time (Supplementary Figs. 27–31). Imidazolium units of 66.0%, 93.6%, 93.9%, and 97.7% in PAImMM(**10**), PAImEE(**12**), PAImPP(**13**), and PAImBB(**14**), respectively, remained intact after 240 h. The stability of PAImXY(**#**) to highly caustic solutions increases with the N-alkyl length, which is consistent with the alkaline stability of the model compounds The two-stage degradation of PAImMM(**10**) observed in low hydration conditions (Fig. 5c, black) and the observation of the inflection point around 20% degradation is also consistent with degradation rate of the model compounds in alkaline/low water content media (Fig. 2f, purple). However, according to the ^1^H NMR analysis, the degradation of the membranes occurs via both dealkylation (major) and ring-opening (minor) pathways, which is different from that of the model compound where only dealkylation was observed. The detection of ring-opening degradation, albeit relatively minor (dealkylation: ring-opening is 4:1), shows up here not only because of the lower *λ*-value (4.3 for 10 M KOH_aq_ vs. 4.8 for 3 M NaOD/D_2_O/CD_3_OD) but possibly due to torsional stress within the polymer, which may reduce the dihedral angle between tetramethylphenyl and the imidazolium ring leading to reduced steric hindrance around the C2-imidazolium.

On the basis of their relatively high anion conductivity and high stability in caustic solutions, PAImEE(**12**) and PAImBB(**14**) were chosen for further study. The anion conductivity of PAImEE(**12**)Cl^−^ and PAImBB(**14**)Cl^−^, measured at various temperatures and humidities, follows an Arrhenius-type behavior shown in Fig. 5e. Both PAImEE(**12**) and PAImBB(**14**) possess a similar *E*_a_ of 24 kJ mol^−1^ at 95% RH (Fig. 5f). The *E*_a_ of PAImBB(**14)** is always higher than that of PAImEE(**12**) below 95% RH, because there is a difference in the dependence of *E*_a_ with humidity. Whereas *E*_a_ for PAImBB(**14**) increases linearly as the humidity is decreased over the range measured, *E*_a_ for PAImEE(**12**) increases slowly with decreasing RH until RH = 40%, at which point *E*_a_ increases more rapidly. This suggests that at RH < 40%, a change in hydration level causes a significant change in the activation of chloride ion transport for PAImEE(**12**). This is consistent with the lower water sorption properties of PAImBB(**14**) compared with PAImEE(**12**), with the fact that a decreasing quantity of imbibed water will ultimately lead to poorly connected hydrophilic regions and a larger energy barrier for ion transport.

### Morphology

**Fig. 6:**
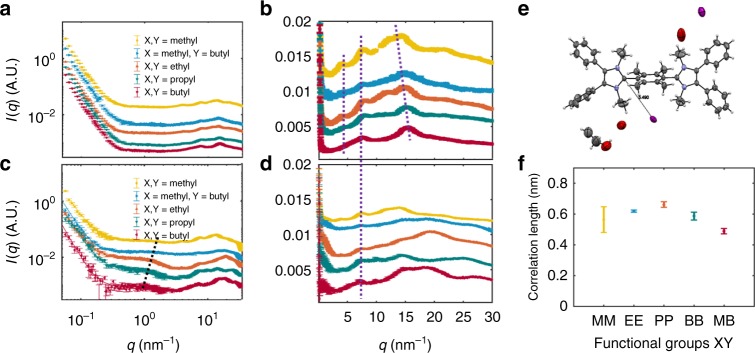
X-ray scattering profiles of PAImXY(#) and X-ray crystal structure of **12**. X-ray scattering profiles of PAImXY(**#**)(I-) in vacuo (**a**, **b**) and hydrated (**c**, **d**). Figures in the left hand column (**a**, **c**) are plotted on a log–log scale to show overall structure. Figures in the middle column (**b**, **d**) are plotted lin–lin to focus on the wide-angle scattering (smaller structure). The curves are vertically offset for clarity. **e** X-ray crystal structure of model compound **12** in its iodide form (ellipsoid set at 50% probability) with distance of iodide to C2 represented by a line. **f** The size of the correlated regions observed in the hydrated samples with different side chains obtained by fitting Equation 6 to the SAXS data

X-ray scattering was employed to determine the morphology of PAImXY(**#**) membranes and explore changes in morphology induced by changing the N-alkyl group. X-ray scattering of the iodide form of PAImMM(**10**) under vacuum (Fig. 6a, yellow) yields a complex scattering pattern: four distinct peaks are located at ~4, 8, 16, and 20 nm^─1^. Only the 16 nm^−1^ peak changes position significantly for polymer analogs bearing longer N-alkyl groups, although the 4 and 20 nm^−1^ peaks weaken in intensity. Only the 8 and 16 nm^−1^ peaks are clearly visible in the scattering profile for PAImBB(**14**) (Fig. 6a, b, red). The 4 nm^−1^ and 8 nm^−1^ peaks correspond to Bragg lengths of ~1.6 and 0.8 nm. As DFT results reveal that the monomer length is 1.6 nm, we interpret these peaks as being the monomer–monomer and anion–anion spacings along the polymer backbone. The 16 nm^−1^ peak corresponds to a Bragg length of ~0.4 nm and is assigned to the distance between the anion and the cationic imidazolium unit. The 16 nm^─1^ peaks shift to higher scattering wavevector (Fig. 6b) as the N-alkyl group is lengthened from methyl to butyl, which implies the distance between anion and imidazolium is decreasing. This is consistent with X-Ray diffraction (XRD) single-crystal analysis of the model compounds **10**–**14**, which shows that anion resides closer to the imidazolium ring as the N-alkyl group increases in length and may be caged within longer N-alkyl groups. Supplementary Figs. 32–36 show single-crystal unit cells with anion-imidazolium distances of 8.95, 5.14, 5.49, 5.28, 4.62 Å, respectively, for **10**, **11**, **12**, **13**, and **14**, respectively. The crystal structure of compound **12** is shown in Fig. 6e as an example here. Although the precise origin of the 20 nm^−1^ is not known, the corresponding length scale is inter-atomic. X-ray scattering plots of hydrated materials are shown in Fig. 6c, d. A scattering knee is apparent at ~1 nm^−1^ for all samples, although more visible in PAImEE(**12**), PAImPP(**13**), and PAImBB(**14**). This scattering is attributed to correlated water-rich hydrophilic regions. The fact that the peak is more prominent in the materials with longer side chains suggests that the water-rich regions are more distinct in these materials. Results of fitting Eq. 6 to the scattering data are shown in Fig. 6f. The correlation length of the water-rich regions increases with N-alkyl length in the materials. The material with mixed N-alkyl units, PAImMB(**11**) shows a shorter correlation length, although the knee was extremely weak in this data. Transmission electron microscopic analyses also indicate that phase segregation becomes more distinct as alkyl chain attached to the N-groups is lengthened (Supplementary Fig. 37). Despite increased phase segregation being synonymous with enhanced water-rich regions and enhanced ionic pathways for promoting ion conductivity^35–40^, this is not the case in PAImXY(**#**), because in this instance increasing the N-alkyl length not only cages the anions but also reduces the IEC and hence water content, resulting in poorly connected hydrophilic regions, which explains the lower *E*_a_ of PAImEE(**12**) compared with PAImBB(**14**) (Fig. 5f).

### Analyses in FCs and water electrolyzers

**Fig. 7:**
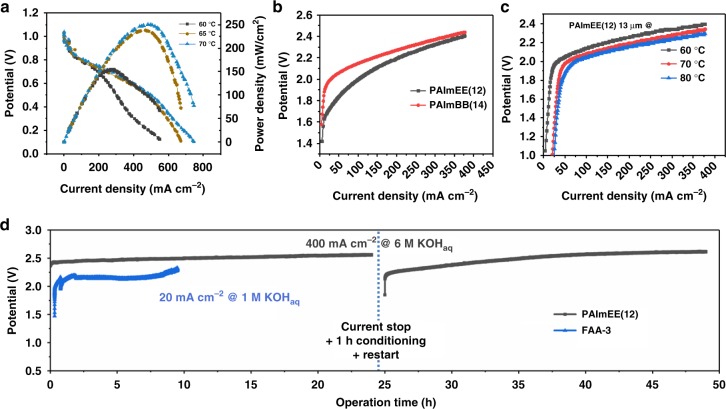
*I*–*V* curves for fuel cells and water electrolyzers. **a** Polarization curves of PAImBB(**14**) AEMFC with 0.5 mg PtRu per cm^2^ (anode) and 0.5 mg Pt per cm^2^ (cathode), and 20 μm membrane thickness. Conditions were 60, 65, and 70 °C, respectively. Here, 1 mL min^−1^, zero back pressure. **b** Polarization curves of PAImEE(**12**) [25 μm thick] and PAImBB(**14**) [20 μm thick] AEM electrolyzer at 60 °C. **c** Polarization curves of PAImEE(**12**) [13 μm thick] AEM electrolyzer at 60, 70 and 80 °C in 6 M KOH. **d** PAImEE(**12**), 25 μm, maintained at 400 mA cm^−2^, and FAA-3, 25 μm, at 20 mA cm^−2^ at 60 °C. The difference in voltage between FAA-3 and PAImEE(**12**) in **c** can be attributed to the different current densities at which the cell was operated

As a proof-of-principle of the applicability of these polymers in electrochemical devices, PAImBB(**14**) membrane was incorporated into an AEMFC, and PAImBB(**14**) and PAImEE(**12**) were each integrated into an AAEM electrolyzer (AEM EL). Electrodes were prepared using solid powder anion-conducting ionomers and catalysts, as reported in details elsewhere^41^. Immediately after equilibration at 60 °C, the cell was polarized without back pressure and a peak power density of 170 mW cm^−2^ was achieved. After increasing cell temperature to 70 °C, peak power density of 250 mW cm^−2^ was achieved, as shown in Fig. 7a. It must be remarked that AEMFC tests cannot typically be operated at temperatures > 60 °C, indicating the enhanced thermal stability of the membranes studied in this work. PAImEE(**12**) and PAImBB(**14**) membranes were incorporated into a water electrolysis cell operated in 6 M KOH liquid electrolyte between 60, 70, and 80 °C. A commercial FAA-3 (Fumatech) AEM operated in 1 M KOH was used for comparison. As shown in Fig. 7b, both PAImEE(**12**) and PAImBB(**14**) membranes were functional as an ionic separator in the water EL. At a current density of 400 mA cm^−2^, the voltage remained below 2.5 V. Higher performance can be achieved by increasing the operating temperature. Figure 7c shows a 0.2 V potential drop from 60 to 80 °C for PAImEE(**12**). Stable operation of membranes at temperatures >60 °C is atypical and again demonstrates enhanced stability of the poly(bis-imidazoliums). A PAImEE(**12**), 25 μm-thick membrane cell was operated at 60 °C with 6 M KOH under 400 mA cm^−2^ for 48 h (Fig. 7d). The break in the cell represents a reconditioning protocol, as trace impurities have been shown to strongly affect the rate of hydrogen evolution^22,42,43^; after reconditioning, the potential was significantly lowered, followed by a gradual increase due to commencement of poisoning of the electrodes. Comparatively, commercial FAA run using the same setup under much milder caustic conditions (1 M KOH) and much lower current density (20 mA cm^−2^) failed after 9.5 h. The PAImEE(**12**) cell was eventually shut down, not because of membrane degradation but due to increased resistance of the uncoated titanium bipolar plate, which had undergone severe corrosion under contact with hot caustic solution. Despite this, the potential remained less than 2.6 V after 48 h, showing promising chemical durability of the PAImEE(**12**) membranes.

## Discussion

Novel polymers have been designed and synthesized based on a molecular design using DFT calculations, investigation of model compounds via crystal structure analysis, and state-of-the-art degradation analysis under low water content conditions. These bis-imidazolium polymers and membranes offer a balanced alkaline stability and high IEC that provides exceptional chemical stability and hydroxide conductivity with low water content. Moreover, a novel versatile synthetic route is presented, which facilitates further investigations of numerous derivations and compositions of these poly(arylimidazolium) hydroxide. Future emphasis should be placed on improving mechanical properties and polymer engineering, such as reinforcement, in order to facilitate their integration in electrochemical energy conversion devices.

## Methods

### Solution degradation test in 3 M NaOD/D_2_O/CD_3_OD

The stability of model compounds were evaluated using a degradation test decribed in the literature^21,24^. A solution of the model compound (0.02 M) was prepared by dissolving the compound in its iodide form in 3 M NaOD with CD_3_OD/D_2_O (7:3 CD_3_OD:D_2_O). The mixture was heated to 80 °C in a closed polytetrafluoroethylene vial for up to 240 h. At specific times, samples were extracted for ^1^H NMR spectroscopic analysis. The degradation of compounds **10**–**14** was quantified using Equation 1 and chemical shift range shown in Table 2:

1 $${\mathrm{{Relative}}\;\mathrm {imidazolium}\;\mathrm {remaining}}\;\left( {\mathrm{\% }} \right) = 100\left( {\frac{{\frac{{x_t}}{{x_t + y_t + z_t}}}}{{\frac{{x_0}}{{x_0 + y_0 + z_0}}}}} \right)$$

where *x*_*t*_ is the integration value for the aromatic peak region, and *y*_*t*_ and *z*_*t*_ is the degradation product peak (*x*_0_, *y*_0_, and *z*_0_ are *x*_*t*_, *y*_*t*_, and *z*_*t*_ at time 0 h, respectively).

**Table 2:** Chemical shift range used in degradation calculations

Model compounds	Chemical shift range for *x*_*t*_	Chemical shift range for *y*_*t*_	Chemical shift range for *z*_*t*_
**10**	7.75–7.63 p.p.m.	7.63–7.42 p.p.m.	7.75–7.03 p.p.m.
**11**	7.64–7.46 p.p.m.	7.46–7.36 p.p.m.	7.24–7.11 p.p.m.
**12**	7.65–7.44 p.p.m.	7.44–7.32 p.p.m.	7.22–7.14 p.p.m.
**13**	7.65–7.42 p.p.m.	7.42–7.33 p.p.m.	7.24–7.13 p.p.m.
**14**	7.66–7.42 p.p.m.	7.42–7.37 p.p.m.	7.32–7.24 p.p.m.

### Solution degradation test in KOH/DMSO-*d*6

Preparation of water-free hydroxide solution. In a three-neck flask, 18-crown-6 (CE) (Sigma-Aldrich) was heated to 60 °C under argon environment. Potassium metal (Merck) was added to the flask to get a blue solution. A precise amount of mili-Q water was injected to the solution, which changed its color to brown, to produce ultra-dry potassium hydroxide, (CE-K^+^) OH^−^. The mixture was then cooled to room temperature and stored in a glove box under a dry inert atmosphere.

All stability tests were carried out with a hydration level of *λ* = 1 (1 water molecule per OH^−^). For each test, two solutions were prepared in a glove box: (1) 0.035 mmol of QA salt was dissolved in 91 μL DMSO-*d*6; (2) 0.5 mmol OH^−^ from the (CE-K^+^) OH^−^ mixture was dissolved in 500 μL DMSO-*d*6. Then, 1.5 μL of internal standard (mesitylene) and 9 μL of mili-Q water were injected to the NMR tube just before the measurement. The hydroxide concentration was measured by titration and confirmed to be 0.5 M.

### Casting procedure

The PAImXY(**#**) polymer in iodide form was dissolved in 3 mL of hot DMSO, filtered onto a flat casting table, and allowed to slowly dry at 86 °C for at least 4 h in air. The film was peeled off the glass as a transparent brown film and then washed with 2 M KOH three times (alternatively changed the solution per day) followed by 2 M HCl and 1 M NaCl to get a clean transparent chloride form membrane. The films produced by this method were transparent and were ~10 μm thick.

### Mechanical strength

The membranes were die-cut to a barbell shape using a standard ASTM D638-4 cutter. The mechanical properties of the membranes were measured under ambient conditions on a single column system (Instron 3344 Series) using a crosshead speed of 5 mm min^−1^. The determined tensile strength, Young’s moduli, and elongation at break were averaged over four samples. The error reported is the SD of four measurements.

### Ionic conductivity

Membrane pieces of in their stable chloride form were used for the conductivity measurements. The ionic resistance was measured in the in-plane direction using a two-point probe by electrochemical impedance spectroscopy. Specifically, an AC potential was applied over a frequency range of 100–10^7^ Hz with a Solartron SI 1260 impedance/gain-phase analyzer at room temperature and in water. For conductivity measurement at various temperature and RH, all membranes were equilibrated 40 min for every 10° rise in temperature 2 h for every 10% increase in RH. The membrane charge transfer resistance (*R*) determined from a best fit of a standard Randles circuit to the measured Nyquist plot was used to calculate the ionic conductivity (*σ*) with Equation 2:

2 $$\sigma = \frac{l}{{AR}}$$

where *l* is the distance between the two electrodes and *A* is the cross-sectional area of the membrane.

### Theoritical ion-exchange capacity

The IEC of PAImXY(**#**) was calculated from the number of ion pairs per repeat unit of the polymer per molecular weight of that repeat unit.

3 $${\mathrm{IEC}} = \frac{{{\mathrm{Number}}\;{\mathrm{of}}\;{\mathrm{ionic}}\;{\mathrm{pairs}}\;{\mathrm{per}}\;{\mathrm{repeat}}\;{\mathrm{unit}}}}{{{\mathrm{Molecular}}\;{\mathrm{weight}}\;{\mathrm{of}}\;{\mathrm{repeat}}\;{\mathrm{unit}}}}$$

### Water content *λ*

The membrane was exchanged to the chloride form as described in the previous section on ionic conductivity. The hydrated membrane was removed from deionized waterdabbed with Kimwipes^TM^, to remove any excess water on the surface, and weighed immediately (*W*_w_). The “wet” membrane was then dried under vacuum at 80 °C to obtain a constant dry weight (*W*_d_). Water uptake (*W*_u_) for three samples was calculated using Equation 4 and the SD of measurements is reported.

4 $$W_{\mathrm{u}} = \frac{{W_{\mathrm{w}} - W_{\mathrm{d}}}}{{W_{\mathrm{d}}}}$$

Lambda, i.e., the number of water molecules per mobile anion, was calculated using the following equation.

5 $$\lambda = 10\frac{{W_{\mathrm{u}}}}{{{{\mathrm{Mw}}_{\mathrm{water}}} \times {\mathrm{IEC}}}}$$

### Transmission electron microscopy

The morphologies of the membranes in their as-cast iodide form were observed using a transmission electron microscope (JEOL JEM-3010 h). The dry membrane was embedded into epoxy resin cut into 100 nm-thick sections using a diamond knife ultramicrotome.

### Membrane degradation test

PAImXY(**#**) membranes were converted to their chloride form by soaking in 1 M NaCl_aq_ for 48 h (exchanging the solution with fresh 1 M NaCl after 12 h) and then washed with deionized water over 48 h with multiple exchanges of water. The membranes were immersed 10 M NaOH_aq_ in closed FEP containers inside an 80 °C oven for up to 240 h. The membranes were removed and converted back to their chloride form by repeating the 1 M NaCl_aq_ soak (48 h) and water wash (48 h). After drying, the membranes were dissolved in DMSO-*d*6 for ^1^H NMR spectroscopic analysis.

### Small angle X-ray scattering

X-ray scattering measurements were performed using a SAXSLab Ganesha 300XL equipped with a mobile PILATUS3 R 300 K photon counting detector. The instrument utilizes a copper anode source, operating at 50 kV and 0.6 mA, which produces radiation with a wavelength of 1.54 Å. Four different configurations were utilized to provide a usable *q*-range of ~0.05 to 3 nm^−1^. Measurements were performed in vacuum and after hydration in deionized water. Samples measured in vacuum were allowed to equilibrate within the evacuated instrument chamber for 1 h. Hydrated samples were immersed in deionized water for 1 h, patted dry with tissue, and measured in sealed cells. Two model functions were fit to the data. The Correlation Length Model was fit to the mid-*q* feature visible in the wet data to extract a characteristic length scale from the data. This function is given by

6 $$I\left( q \right) = \frac{A}{{q^n}} + \frac{C}{{1 + \left( {q\xi } \right)^m}} + B$$

where *A* and *C* are scale factors, *ξ* is the correlation length, *n* is the low-*q* Porod exponent, *m* is the mid-*q* Porod exponent, and *B* is the *q*-independent background. As it tended to become extremely large in measurements where the feature was weak, *m* was fixed at 4, the best-fit value in measurements where the feature was very clear.

A series of pseudo-Voigt functions, which provide a flexible peak shape [ref], was fit to the measurements of PAImBB in vacuum, in order to estimate the *q*-independent scattering and produce a background-subtracted plot. This fitting function is given by

7 $$I\left( q \right) = \frac{A}{{q^n}} + \mathop {\sum}\nolimits_i {\left\{ {\begin{array}{*{20}{c}} {S_i\;A_{i}{\mathrm{exp}}( - \left( {\frac{{2\left( {q - b_i} \right)}}{{c_i}}} \right)^2\log (2)) + } \\ {A_i\frac{{1 - S_i}}{{1 + \left( {2\left( {\frac{{q - b_i}}{{c_i}}} \right)} \right)}}} \end{array}} \right\} + B}$$

where *A* and *A*_*i*_ are scale parameters, *S*_*i*_ are peak shape parameter, *b*_*i*_ are peak positions, and *c*_*i*_ are peak widths.

### Single-crystal XRD

X-ray data were collected at ambient conditions on a Bruker Smart instrument equipped with an APEX II CCD area detector fixed at a distance of 5.0 cm from the crystal and a Cu Kα fine focus sealed tube (1.54178 Å) operated at 1.5 kW (50 kV, 30 mA), filtered with a graphite monochromator. Diffraction data were processed with Bruker Apex II software suite. The structures were solved with direct methods (SIR92) and subsequent refinements were performed using ShelXle34. MeIm was crystallized from water in its iodide form. Crystallographic information for compounds **10**–**14** were shown in Supplementary Tables 2 and 3. The crystallographic data (CCDC 1852658–1852662) can be obtained free of charge from The Cambridge Crystallographic Data Centre via www.ccdc.cam.ac.uk/getstructures.

### DFT calculations

Structures of compound **8**–**14** and ESP were performed using B3LYP DFT under Gaussian G0933. The Polarizable Continuum Model implemented in G09 used an Integral Equation Formalism with water as solvent (*ε* = 78.36). All structures were pre-optimized using 6–31 G(d) basis set and refined with 6-311 + + G(2d,2p) basis set, tight convergence criteria. The following contains the coordinates of the model compounds (Supplementary Tables 4–10).

### FC analyses

Catalyst loadings of 0.5 mg_Pt_ cm^−2^ were used in both anode (PtRu) and cathode (Pt) electrodes. The electrodes, coated onto Toray 60 gas diffusion layers, were placed on opposite sides of the PAImBB(**14**) AAEM and loaded into FC hardware with a 5 cm^2^ active area. The cell was tested using a Scribner 850e FC test station. After the AEMFC was assembled, the cell was initially brought to 60 °C and humidified.

### Water electrolysis

Catalyst-coated membranes were prepared using the poly(arylimidazoliums) as the membrane and as ionomer in the catalyst layer. Catalyst inks contained 1% solid, 15–20% ionomer, 85–80% Pt on C (46.4% Pt, Tanaka). The electrolysis cell consisted of a membrane electrode assembly compressed between two titanium bipolar plates having serpentine flow fields. Titanium porous transport layers separated the catalyst layers from the flow fields and provided electrical contact, and allowed transport of gasses and liquid. The bipolar plates were housed in titanium hardware equipped with gold current collectors. Liquid was fed to the anode and cathode using peristaltic pumps at a rate of 10.0 mL min^−1^ and recirculated using liquid gas separators.

## Supplementary information

Supplementary Information

## Data Availability

The data that support the plots within this paper and other findings of this study are available from the corresponding authors upon reasonable request.
