# Supplementary material for: Poly(bis-arylimidazoliums) possessing high hydroxide ion exchange capacity and high alkaline stability
Source: Nat Commun. 2019 May 24;10:2306. doi: 10.1038/s41467-019-10292-z (PMC6534565; doi:10.1038/s41467-019-10292-z)
Supplement: Supplementary file 1 — Supplementary Information [file 41467_2019_10292_MOESM1_ESM.pdf]

Supplementary information

Poly(bis-arylimidazoliums) Possessing High Hydroxide Ion Exchange Capacity and High Alkaline Stability

Fan et al.

Supplementary Figures

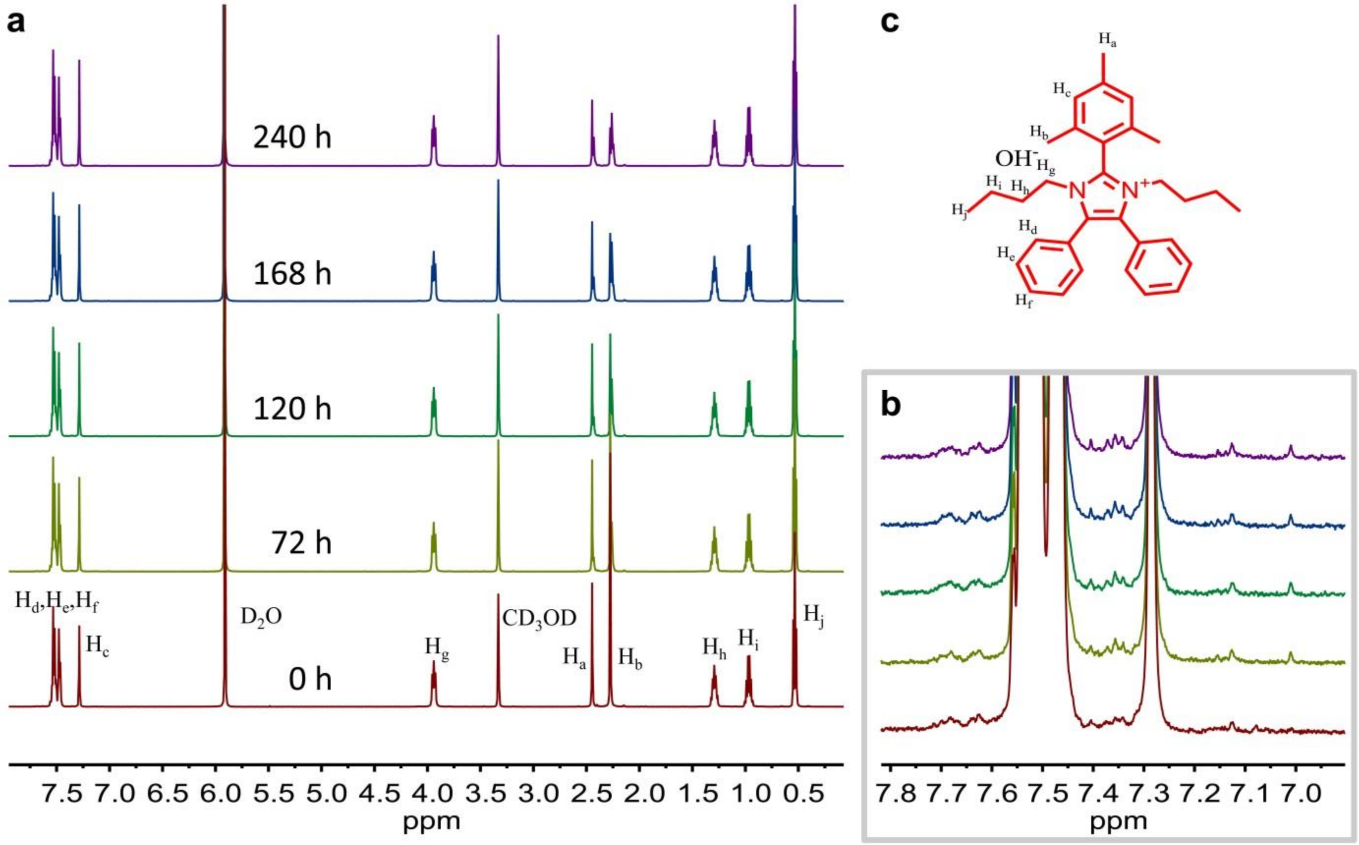

Supplementary Figure 1. **Alkaline stability of the model compound 9 in solution.** a, b, Selected regions of the ^{1}H NMR spectra of **9** (0.02 M) in 3 M NaOD/CD_3OD/D_2O (7:3 wt. CD_3OD:D_2O) after being heated at 80 °C for a given amount of time. The observable decrease in the H_b signal (**a**) is mainly due to deuterium-exchange and not degradation. **c**, the chemical structure of **9**.

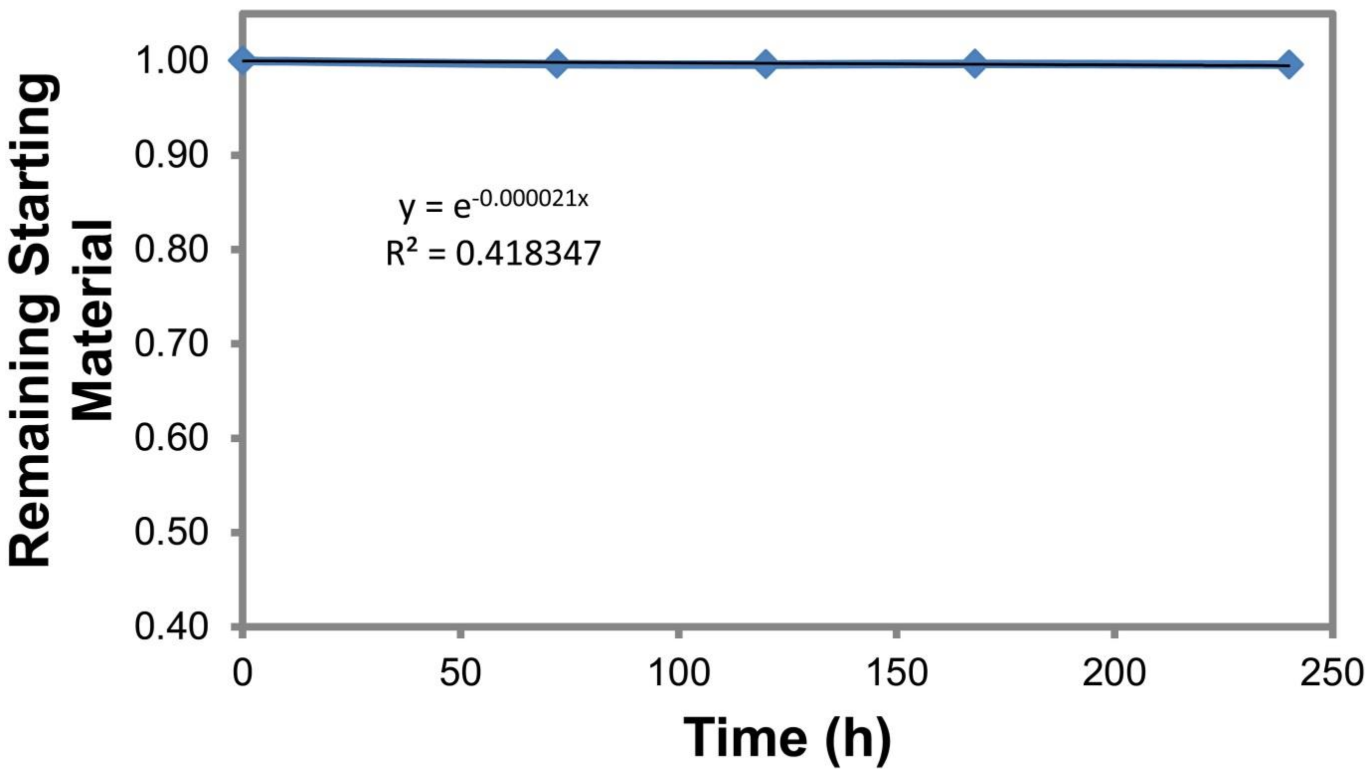

Supplementary Figure 2. Percent **9** remaining and fitting curve at a concentration of 0.02 M in a 3 M NaOD solution containing 7:3 wt CD_3OD/D_2O (i.e., 70% wt CD_3OD) at 80 °C over time (as determined by ^1H NMR spectroscopy).

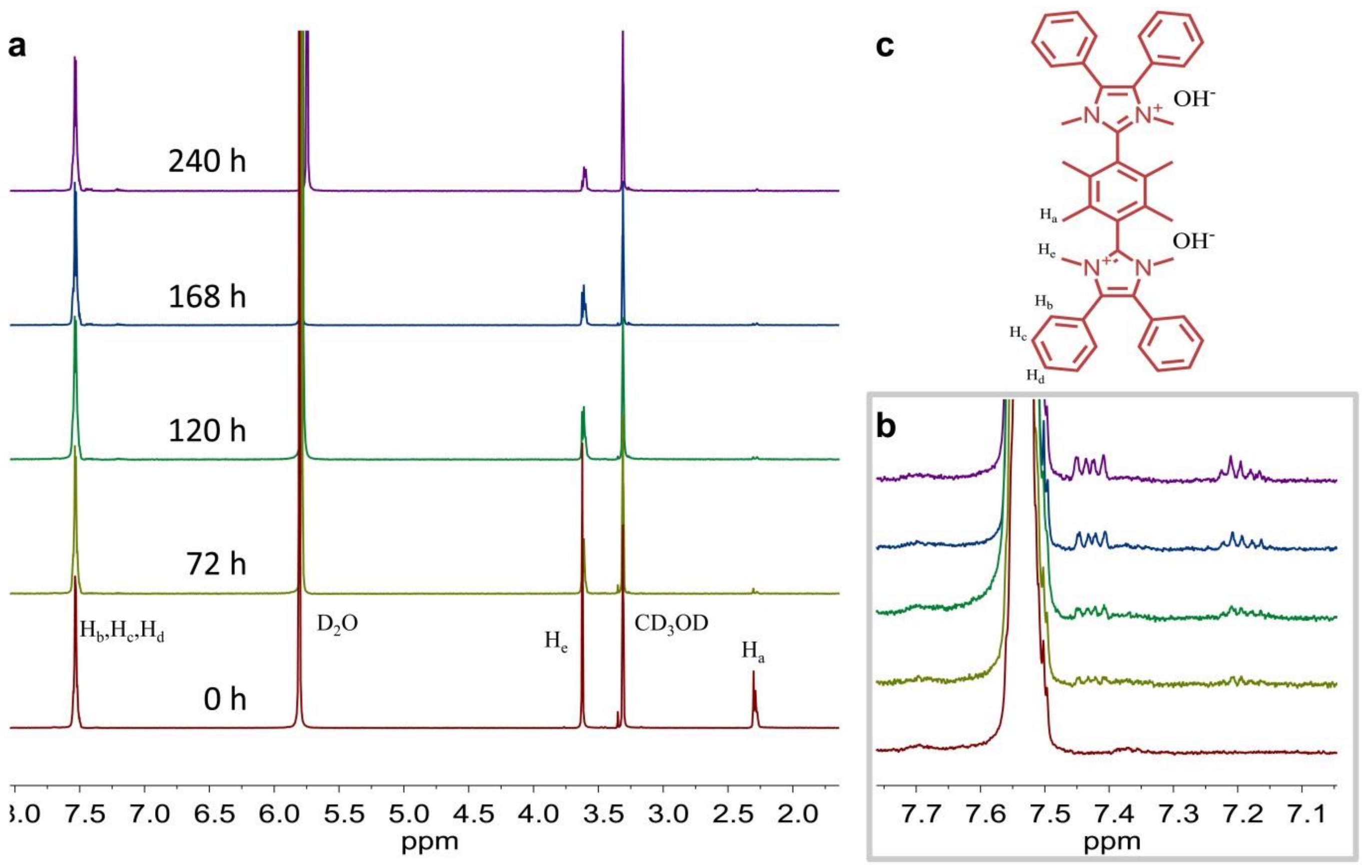

Supplementary Figure 3. **Alkaline stability of the model compound 10 in solution.** a, b, Selected regions of the ^{1}H NMR spectra of **10** (0.02 M) in 3 M NaOD/CD_3OD/D_2O (7:3 wt. CD_3OD:D_2O) after being heated at 80 °C for a given amount of time. The observable decrease in the H_a and H_e signal (**a**) is mainly due to deuterium-exchange and not degradation. **c**, the chemical structure of **10**.

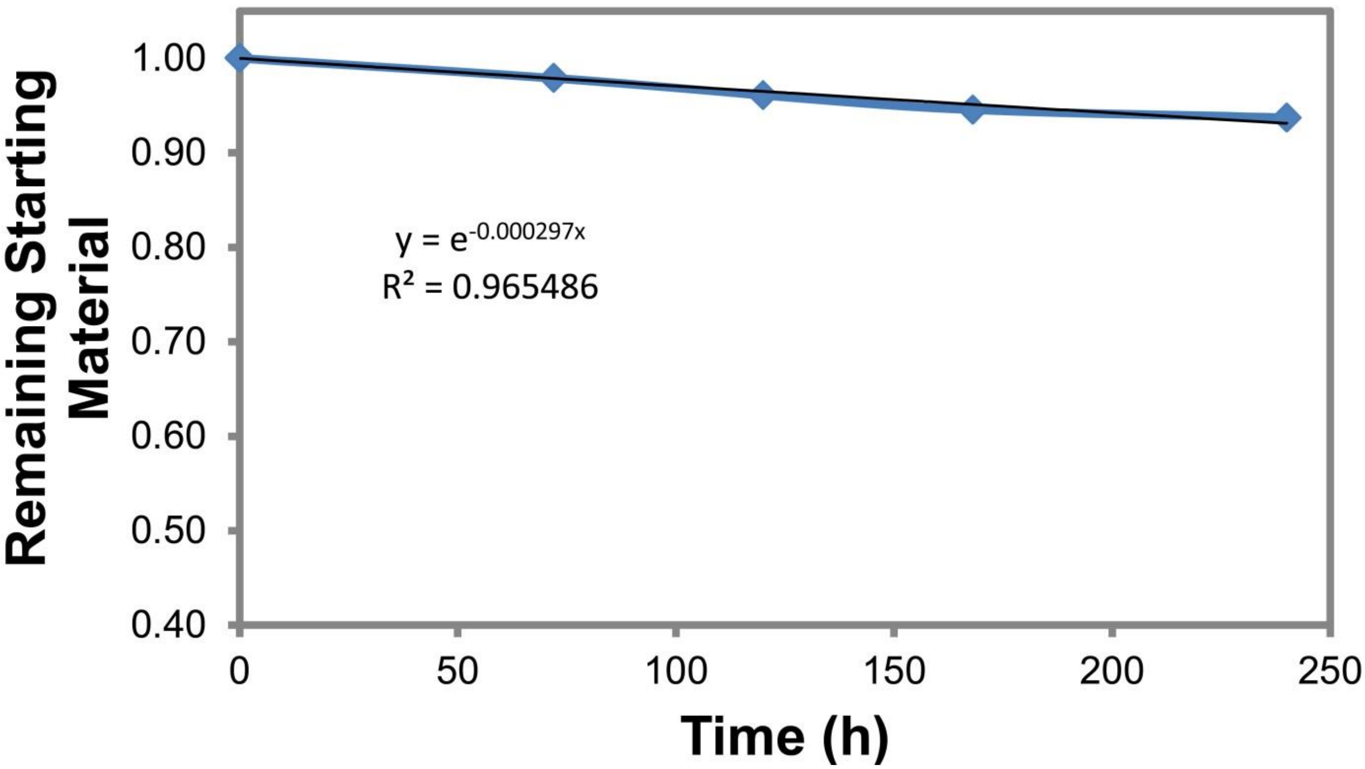

Supplementary Figure 4. Percent **10** remaining and fitting curve at a concentration of 0.02 M in a 3 M NaOD solution containing 7:3 wt CD_3OD/D_2O (i.e., 70% wt CD_3OD) at 80 °C over time (as determined by ^1H NMR spectroscopy).

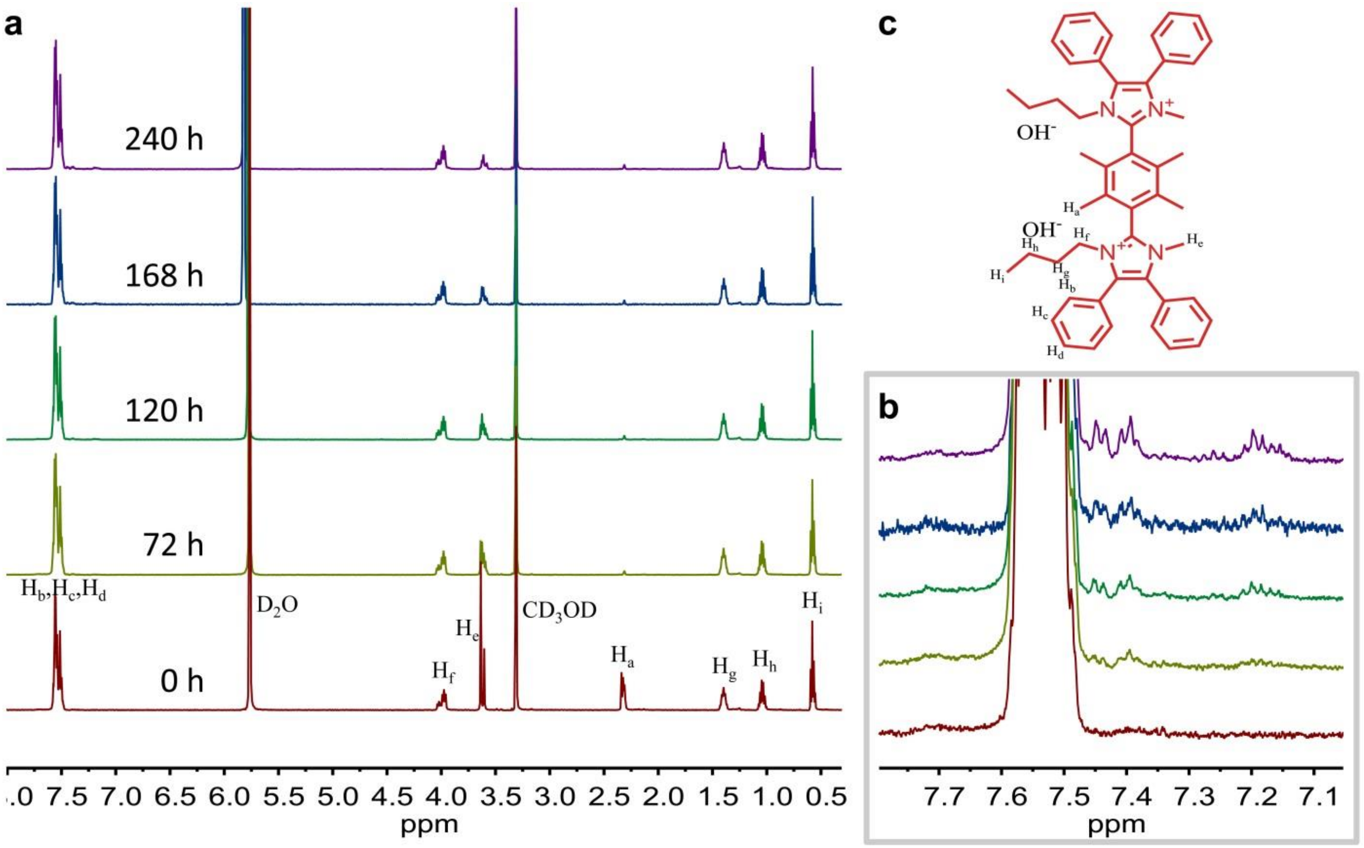

Supplementary Figure 5. **Alkaline stability of the model compound 11 in solution.** a, b, Selected regions of the ^{1}H NMR spectra of **11** (0.02 M) in 3 M $NaOD/CD_3OD/D_2O$ (7:3 wt. $CD_3OD:D_2O$) after being heated at 80 °C for a given amount of time. The observable decrease in the H_a signal (**a**) is mainly due to deuterium-exchange and not degradation. **c**, the chemical structure of **11**.

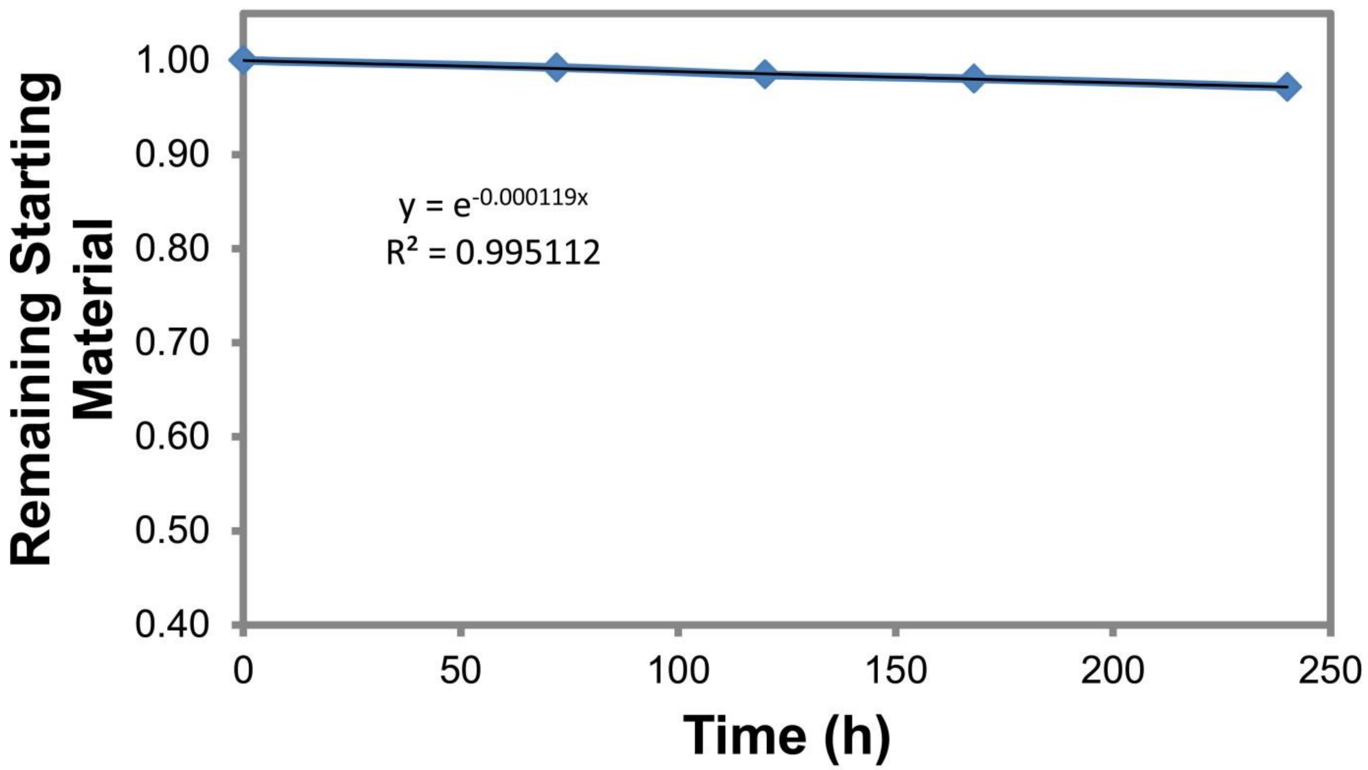

Supplementary Figure 6. Percent **11** remaining and fitting curve at a concentration of 0.02 M in a 3 M NaOD solution containing 7:3 wt CD_3OD/D_2O (i.e., 70% wt CD_3OD) at 80 °C over time (as determined by ^{1}H NMR spectroscopy).

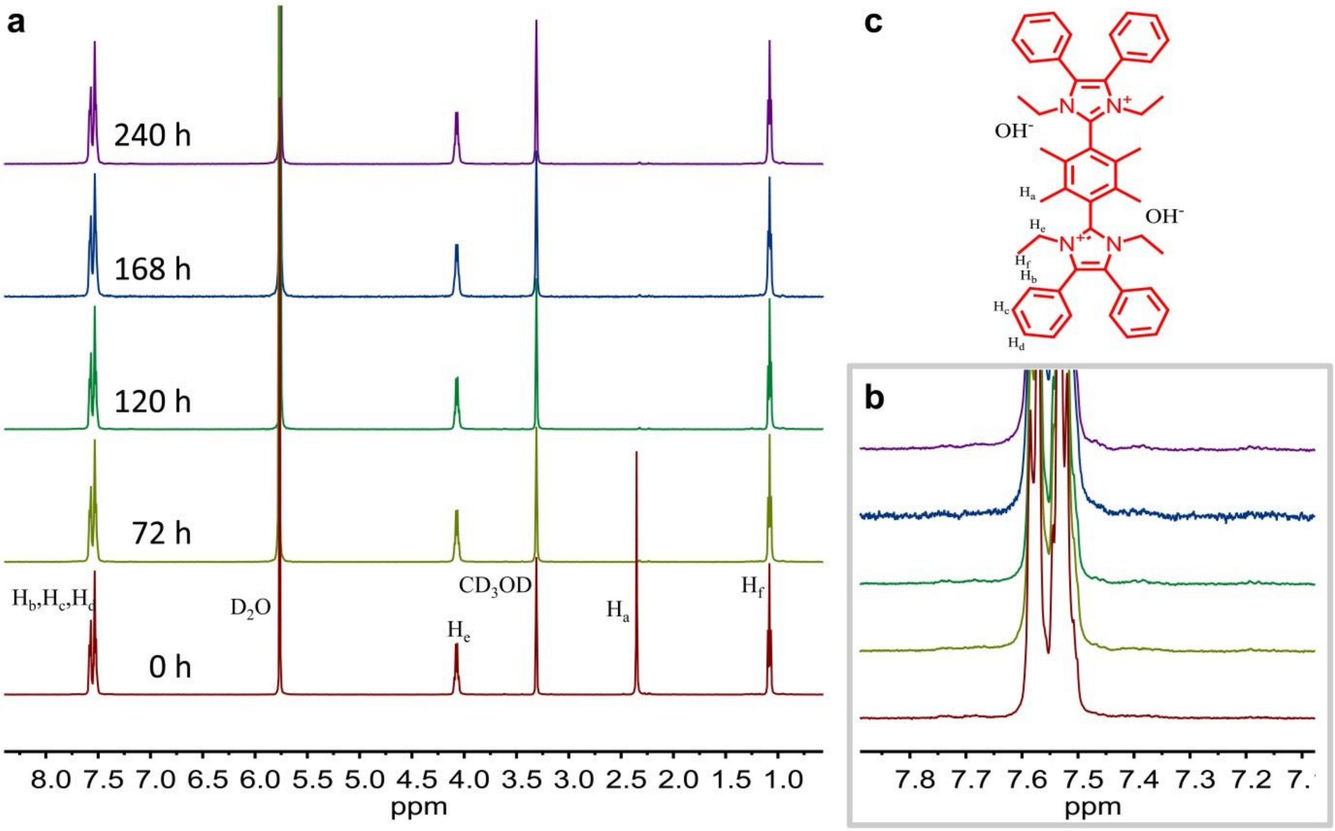

Supplementary Figure 7. Alkaline stability of the model compound 12 in solution. a, b, Selected regions of the ^{1}H NMR spectra of **12** (0.02 M) in 3 M NaOD/CD_3OD/D_2O (7:3 wt. $CD_3OD:D_2O$) after being heated at 80 °C for a given amount of time. The observable decrease in the H_a signal (**a**) is mainly due to deuterium-exchange and not degradation. **c**, the chemical structure of **12**.

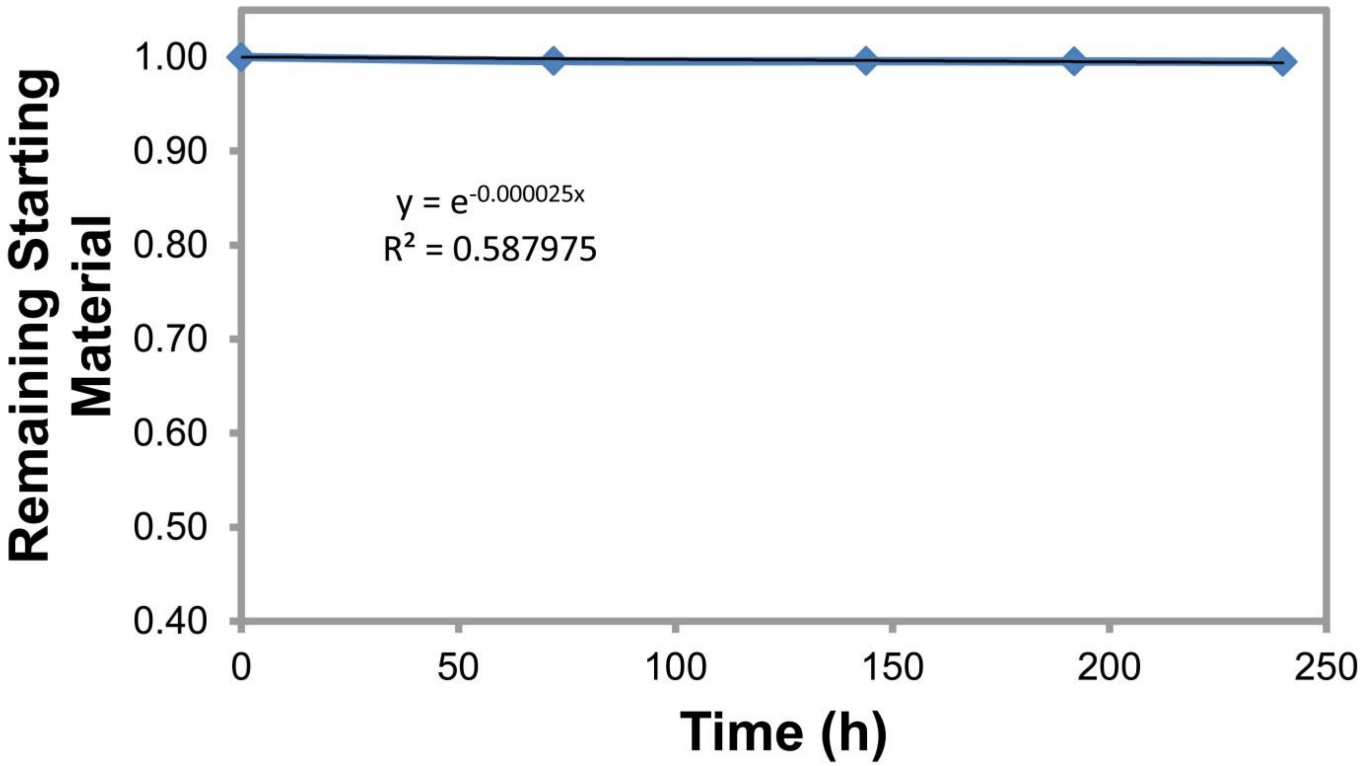

Supplementary Figure 8. Percent **12** remaining and fitting curve at a concentration of 0.02 M in a 3 M NaOD solution containing 7:3 wt CD_3OD/D_2O (i.e., 70% wt CD_3OD) at 80 °C over time (as determined by ^1H NMR spectroscopy).

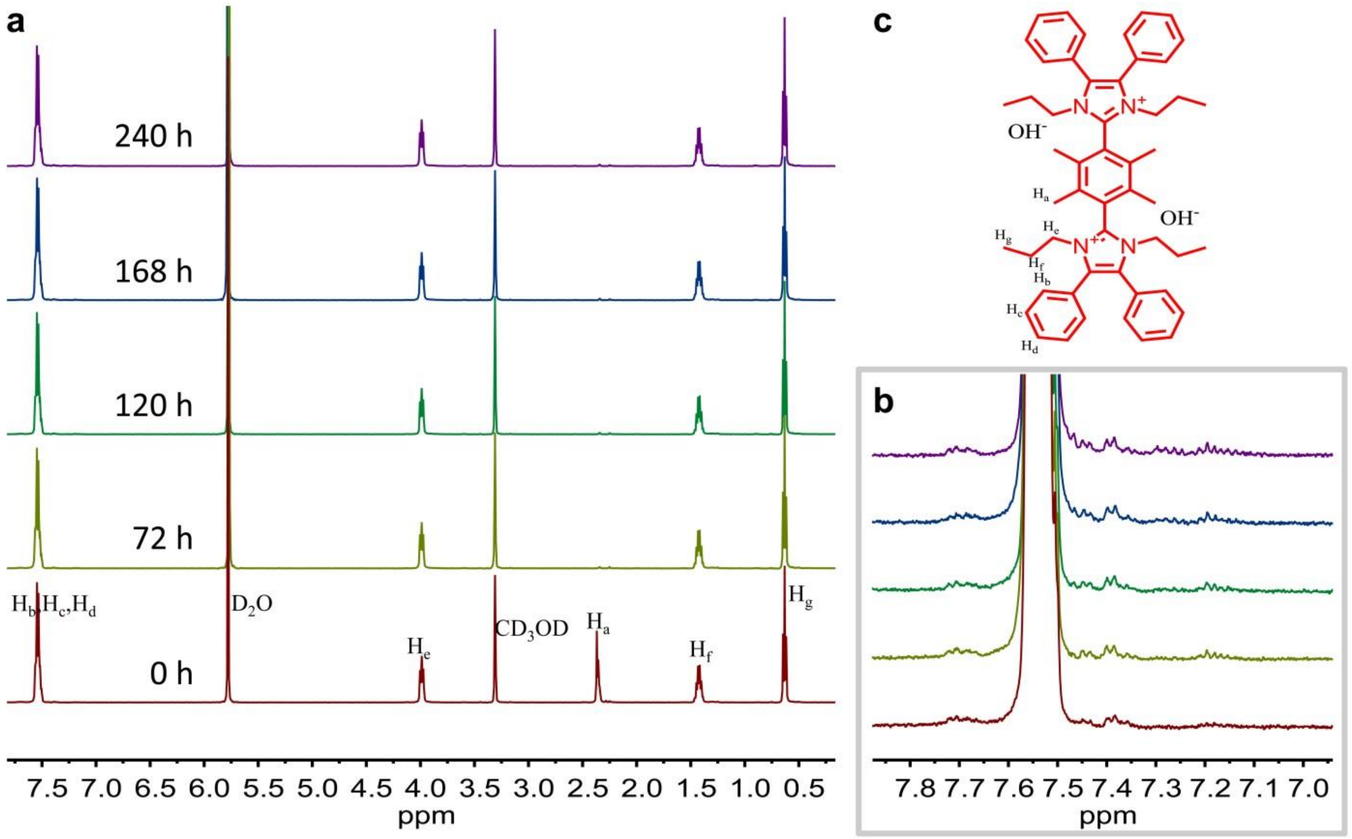

Supplementary Figure 9. **Alkaline stability of the model compound 13 in solution.** a, b, Selected regions of the ^{1}H NMR spectra of **13** (0.02 M) in 3 M NaOD/CD_3OD/D_2O (7:3 wt. CD_3OD:D_2O) after being heated at 80 °C for a given amount of time. The observable decrease in the H_a signal (**a**) is mainly due to deuterium-exchange and not degradation. **c**, the chemical structure of **13**.

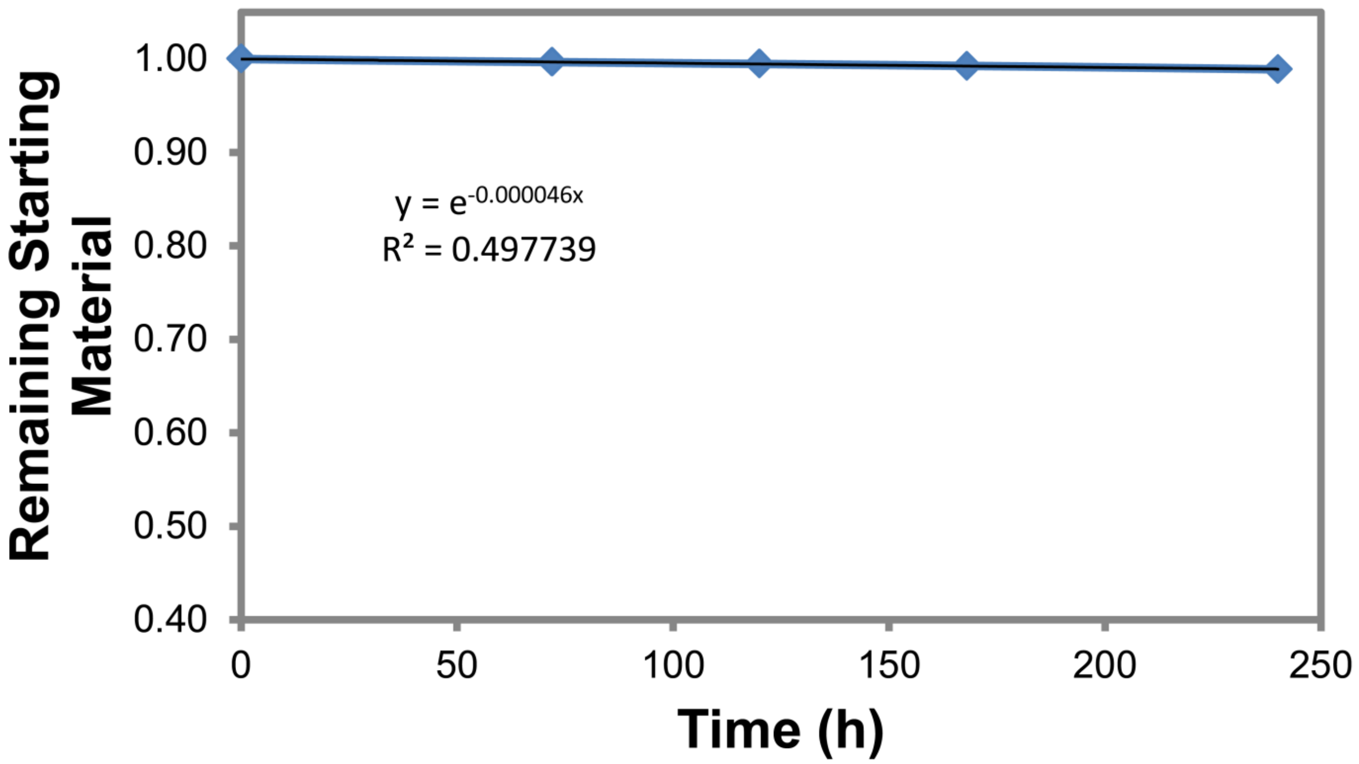

Supplementary Figure 10. Percent **13** remaining and fitting curve at a concentration of 0.02 M in a 3 M NaOD solution containing 7:3 wt CD_3OD/D_2O (i.e., 70% wt CD_3OD) at 80 °C over time (as determined by ^{1}H NMR spectroscopy).

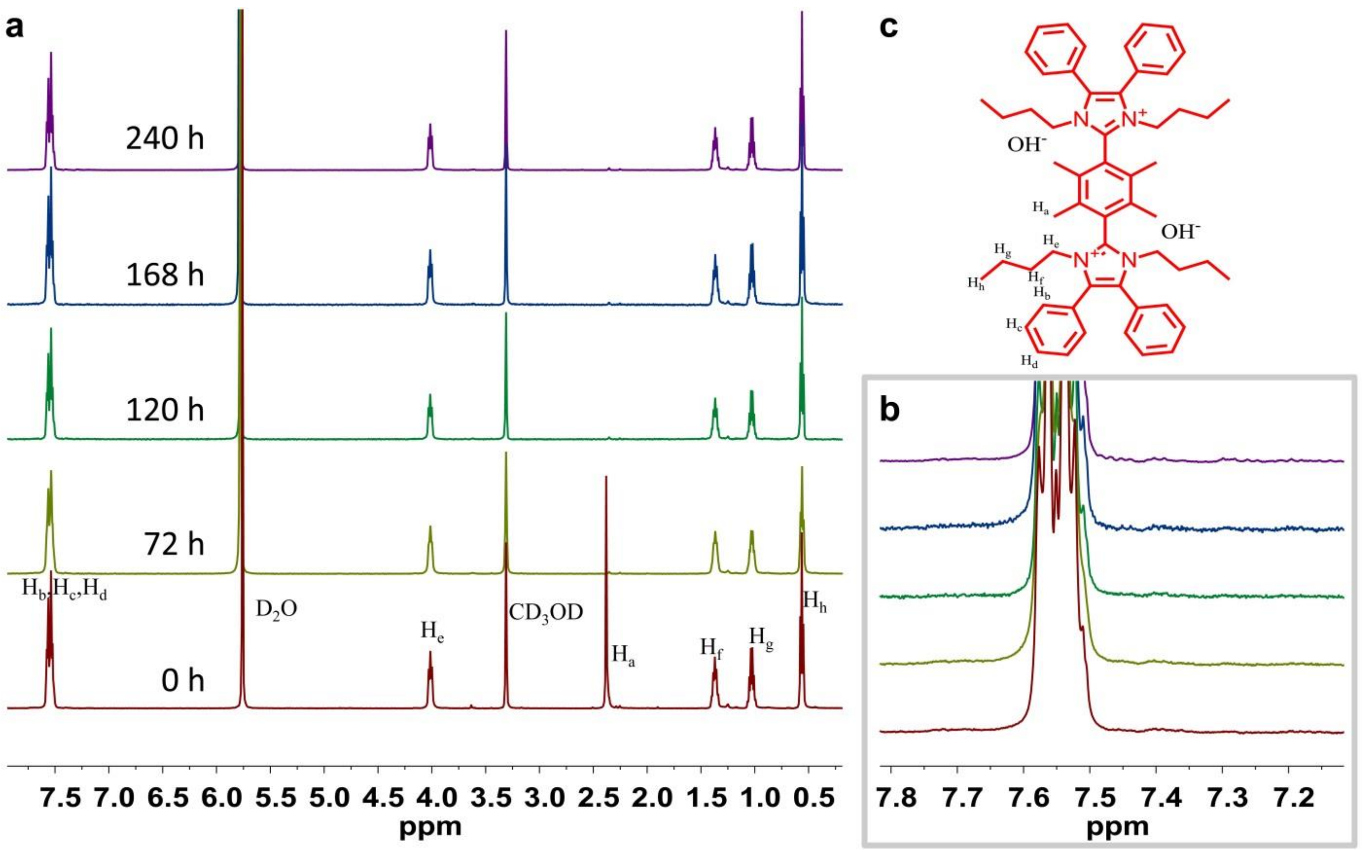

Supplementary Figure 11. **Alkaline stability of the model compound 14 in solution.** a, b, Selected regions of the ^{1}H NMR spectra of **14** (0.02 M) in 3 M NaOD/CD_3OD/D_2O (7:3 wt. CD_3OD:D_2O) after being heated at 80 °C for a given amount of time. The observable decrease in the H_a signal (**a**) is mainly due to deuterium-exchange and not degradation. **c**, the chemical structure of **14**.

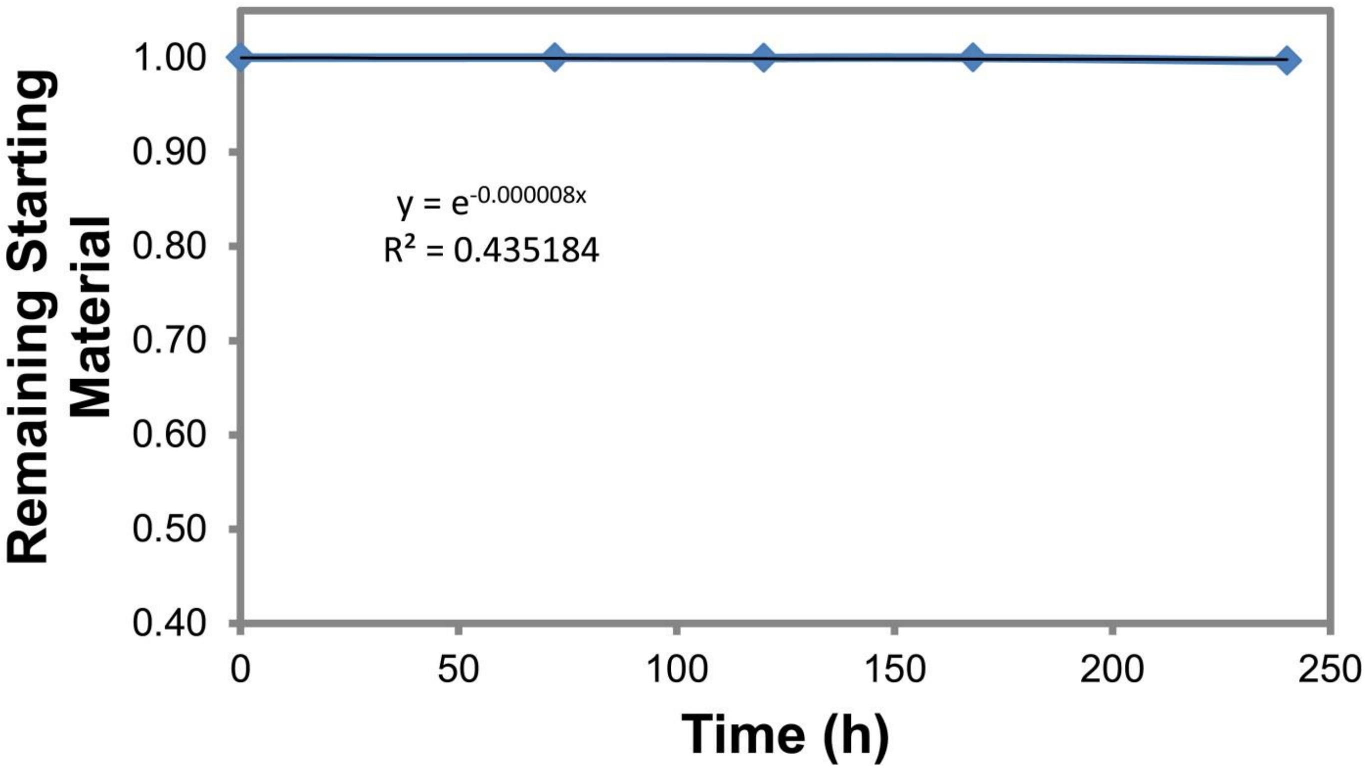

Supplementary Figure 12. Percent **14** remaining and fitting curve at a concentration of 0.02 M in a 3 M NaOD solution containing 7:3 wt CD_3OD/D_2O (i.e., 70% wt CD_3OD) at 80 °C over time (as determined by ^1H NMR spectroscopy).

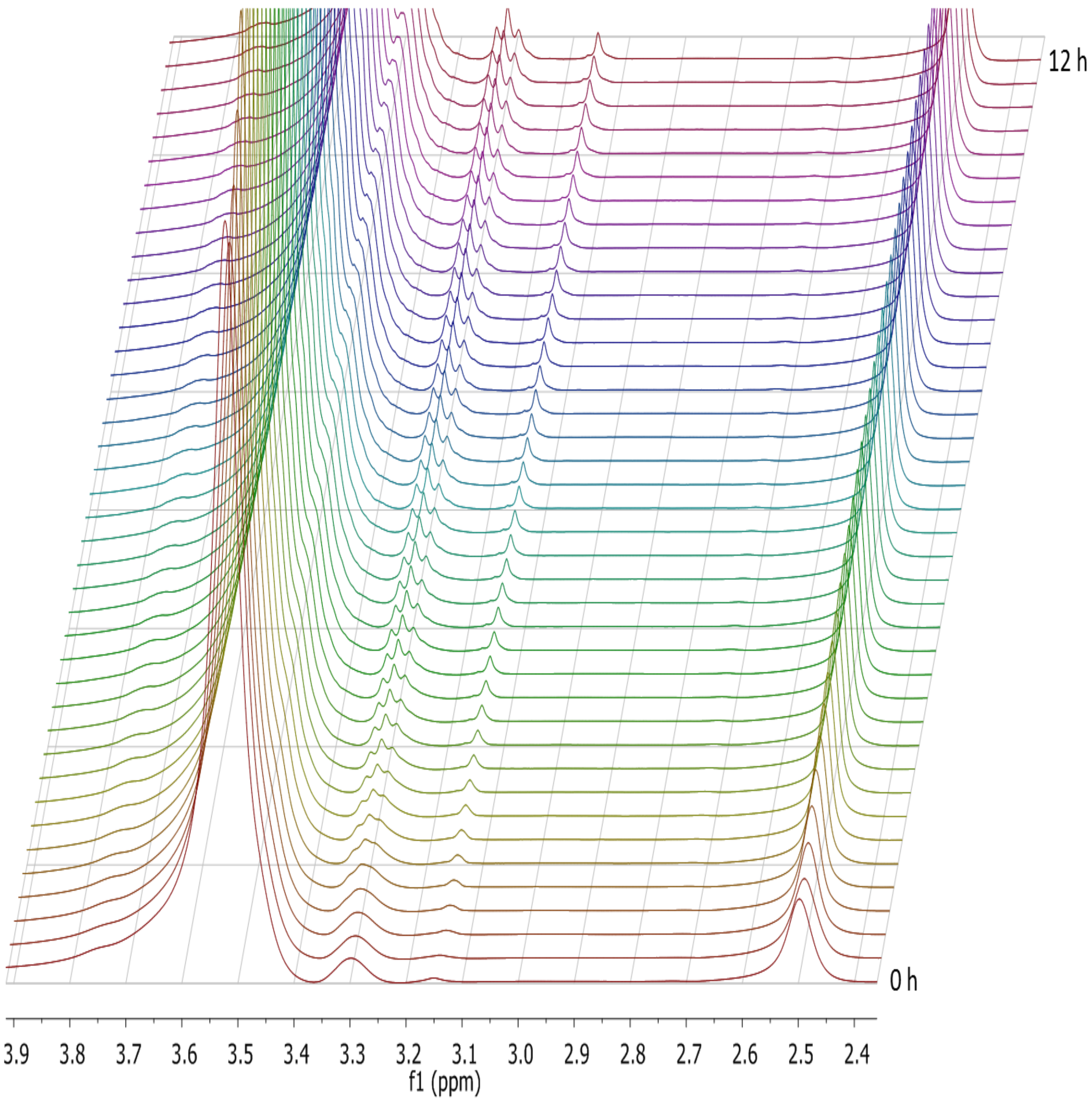

Supplementary Figure 13. Selected region of ^{1}H-NMR kinetics spectra of compound **3** in 0.5 M OH^{-} and λ=1 in DMSO-d_6 at room temperature. The peaks at 2.5, 3.3 and 3.5 ppm correspond to DMSO-d5, water and $(CE\text{-}K)^{+}OH$, respectively. The position of the methyl peak of the compound at 3.1 ppm does not change.

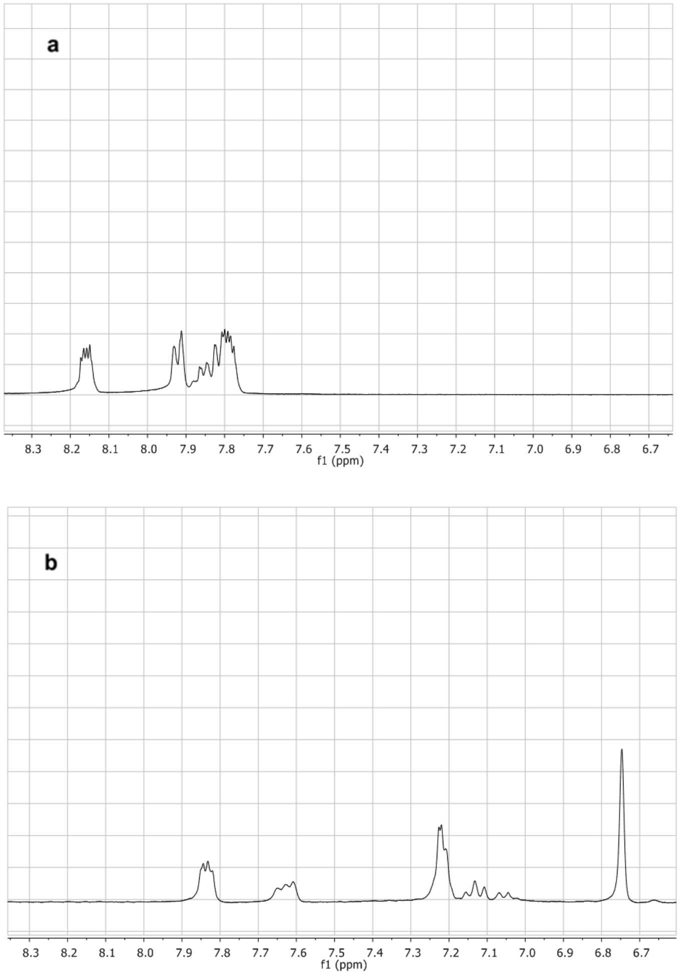

Supplementary Figure 14. The aromatic area of ^{1}H-NMR spectra of compound **4** (top) and after decomposition (bottom) in 0.5 M OH^{-} and λ=1 in DMSO-d_6 at room temperature. The aromatic peak of the internal standard mesitylene group appears at 6.7 ppm.

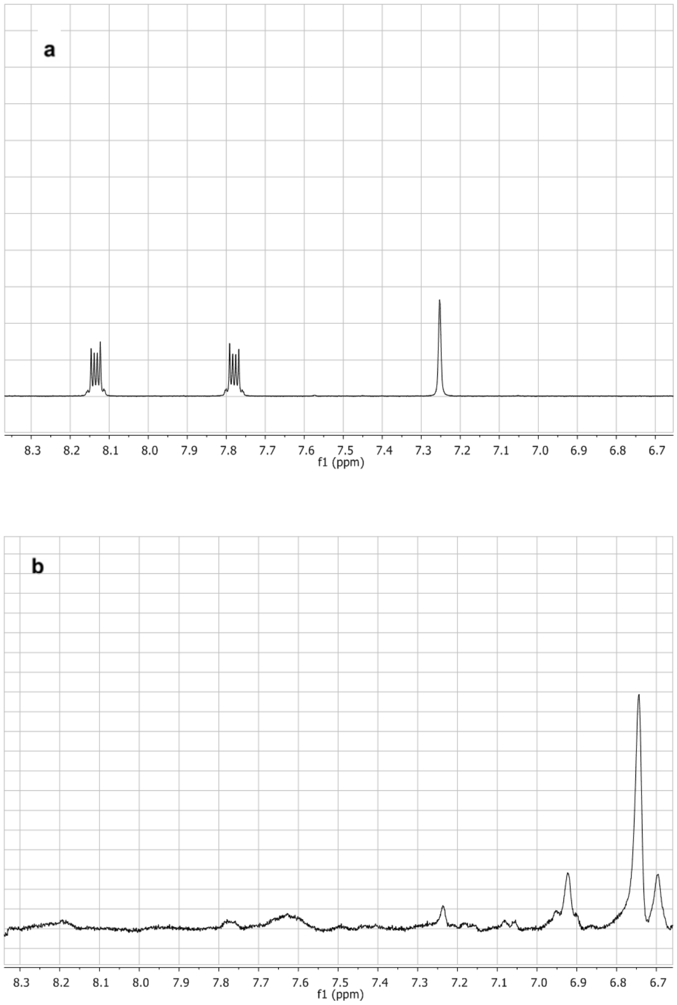

Supplementary Figure 15. The aromatic area of ^{1}H-NMR spectra of compound **5** (top) and after decomposition (bottom) in 0.5 M OH^- and λ=1 in DMSO-d_6 at room temperature. The aromatic peak of the internal standard mesitylene group appears at 6.7 ppm.

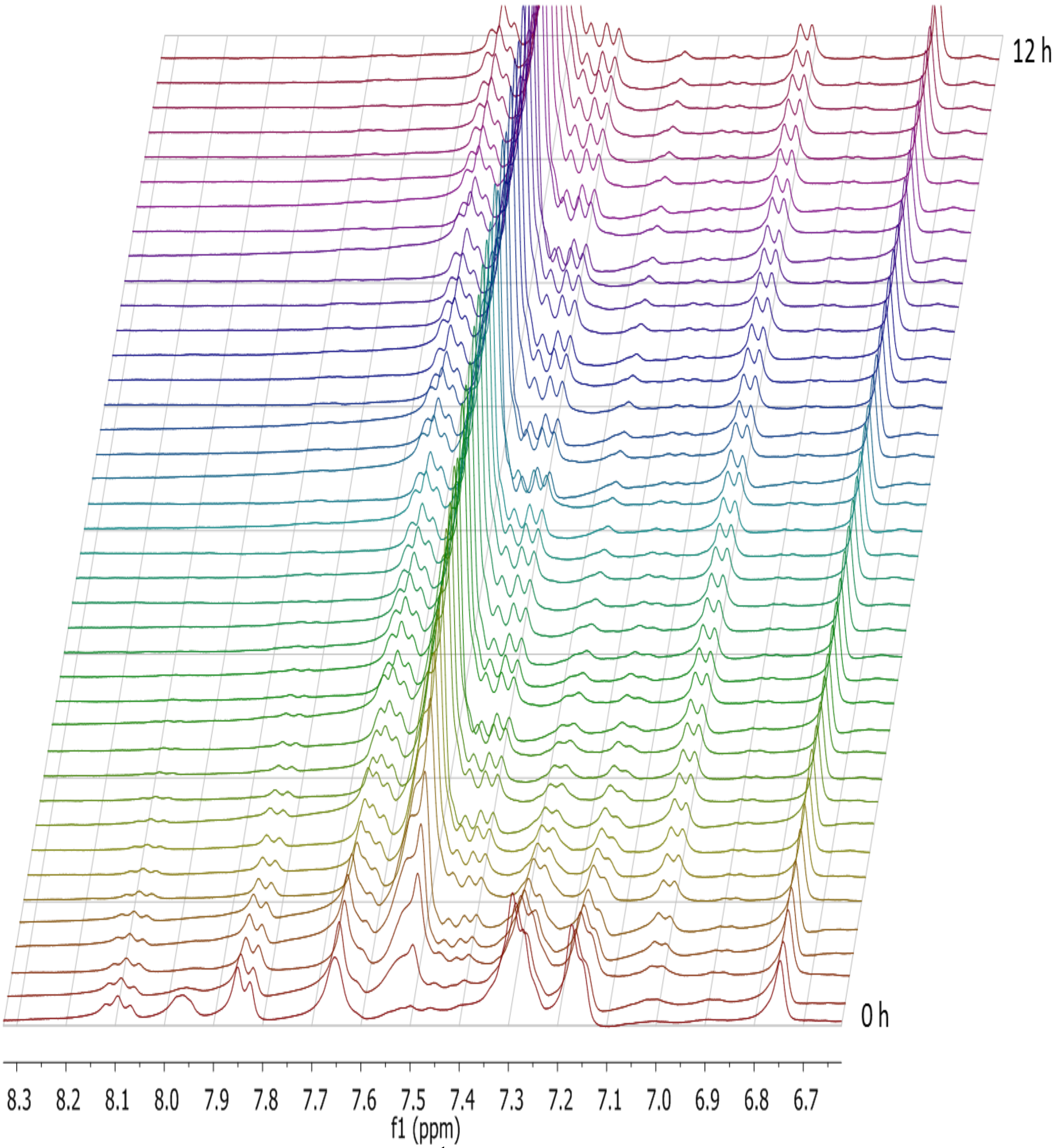

Supplementary Figure 16. Selected region of ^{1}H-NMR kinetics spectra of compound **6** in 0.5 M OH^{-} and λ=1 in DMSO-d_6 at room temperature. The aromatic peak of the internal standard mesitylene group appears at 6.7 ppm. The aromatic peaks of the compound at 7.15, 7.30, 7.65, 7.85 and 8.10 ppm decrease while the degradation product peaks at 7.0, 7.4 and 7.7 ppm increase.

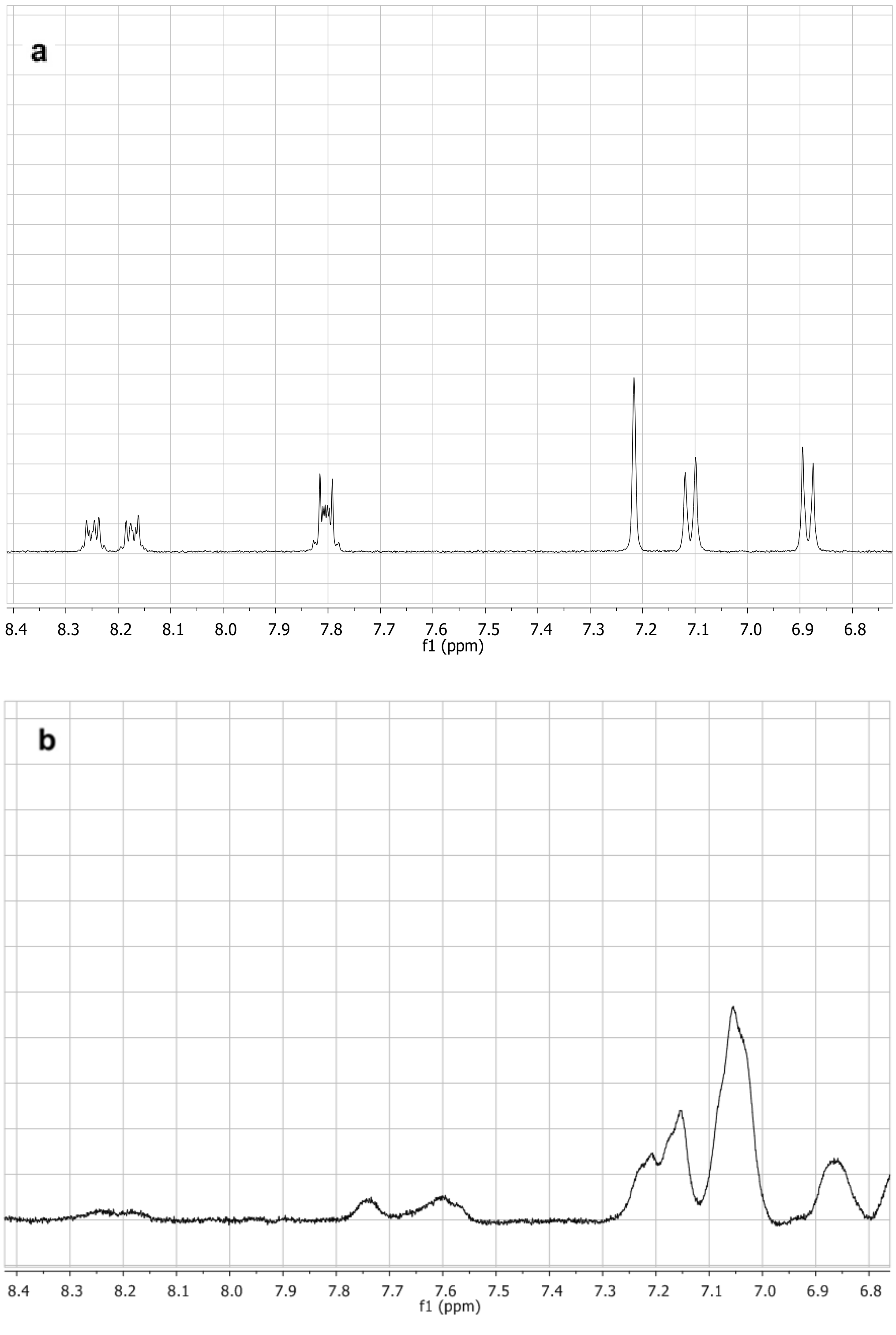

Supplementary Figure 17. The aromatic area of ^{1}H-NMR spectra of compound **7** (top) and after decomposition(bottom) in 0.5 M OH^{-} and λ=1 in DMSO-d_6 at room temperature.

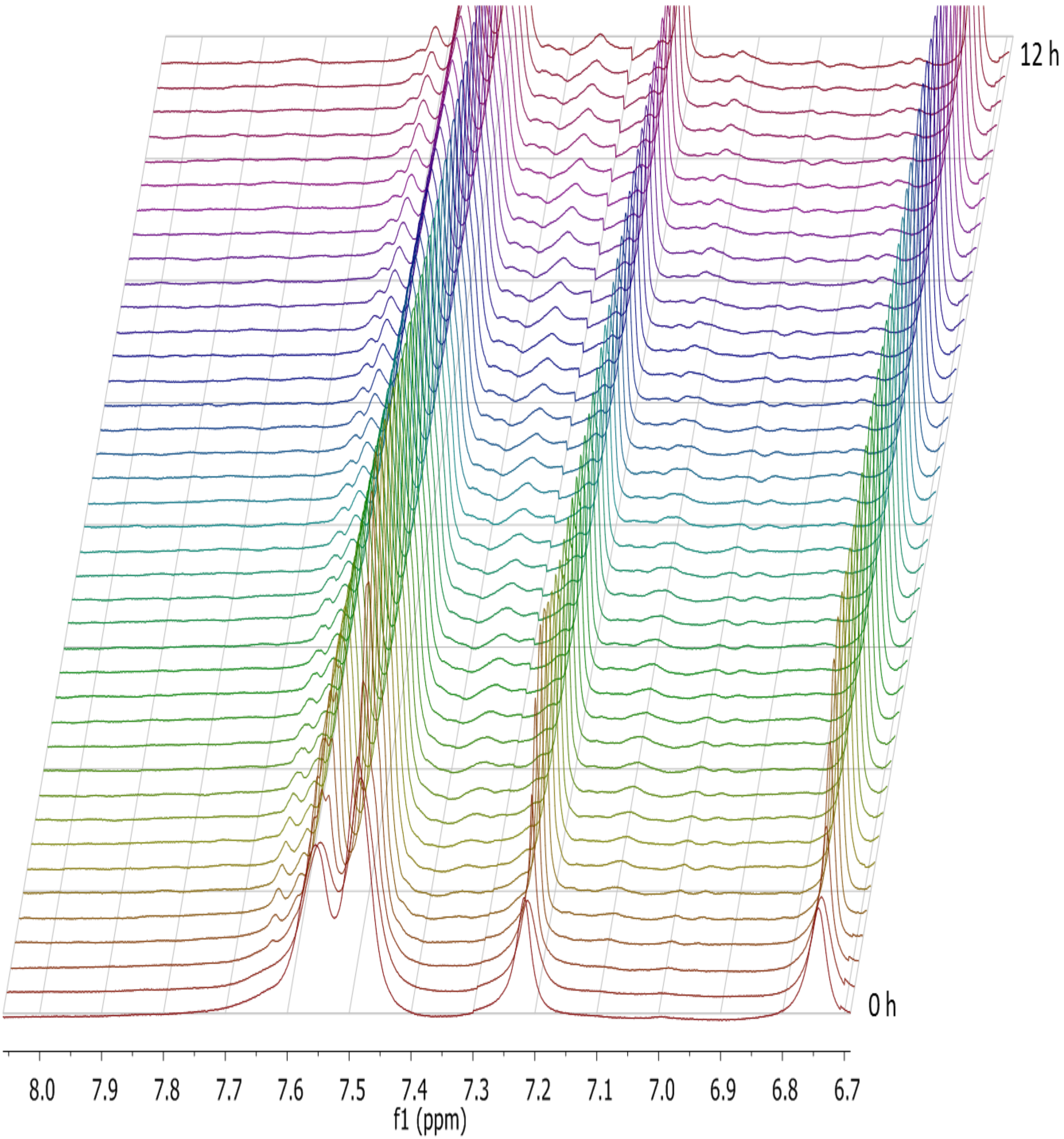

Supplementary Figure 18. Selected region of ^{1}H-NMR kinetics spectra of compound **8** in 0.5 M OH^- and λ=1 in DMSO-d_6 at room temperature. The aromatic peak of the internal standard mesitylene group appears at 6.7 ppm. The aromatic peaks of the compound at 7.2 and 7.4-7.6 ppm decrease while the degradation product peaks at 7.35 ppm increase.

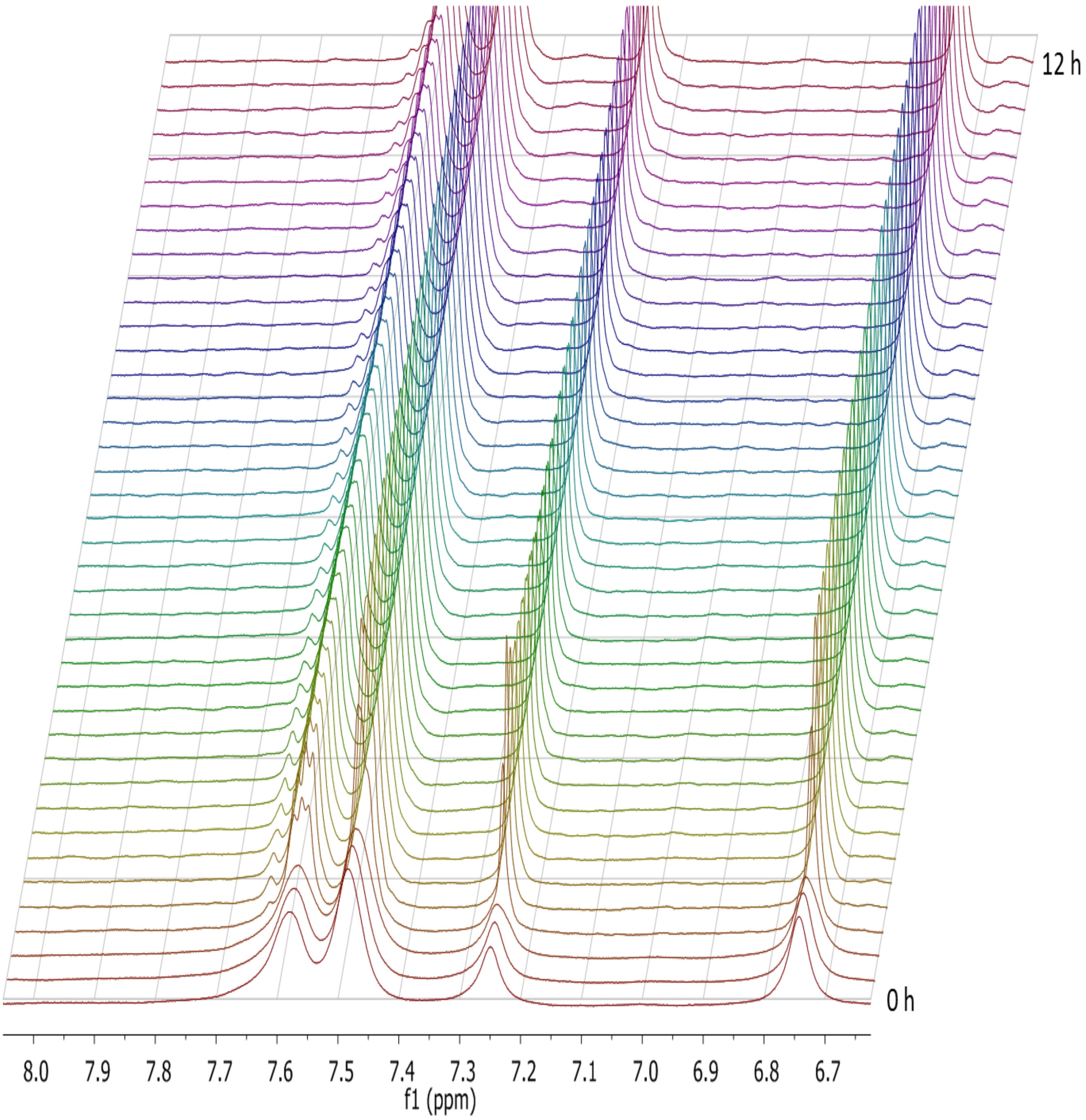

Supplementary Figure 19. Selected region of ^{1}H-NMR kinetics spectra of compound **9** in 0.5 M OH^- and λ=1 in DMSO-d_6 at room temperature. The aromatic peak of the internal standard mesitylene group appears at 6.7 ppm. The aromatic peaks of the compound at 7.2 and 7.4-7.6 ppm decrease while the degradation product peaks at 7.35 ppm increase.

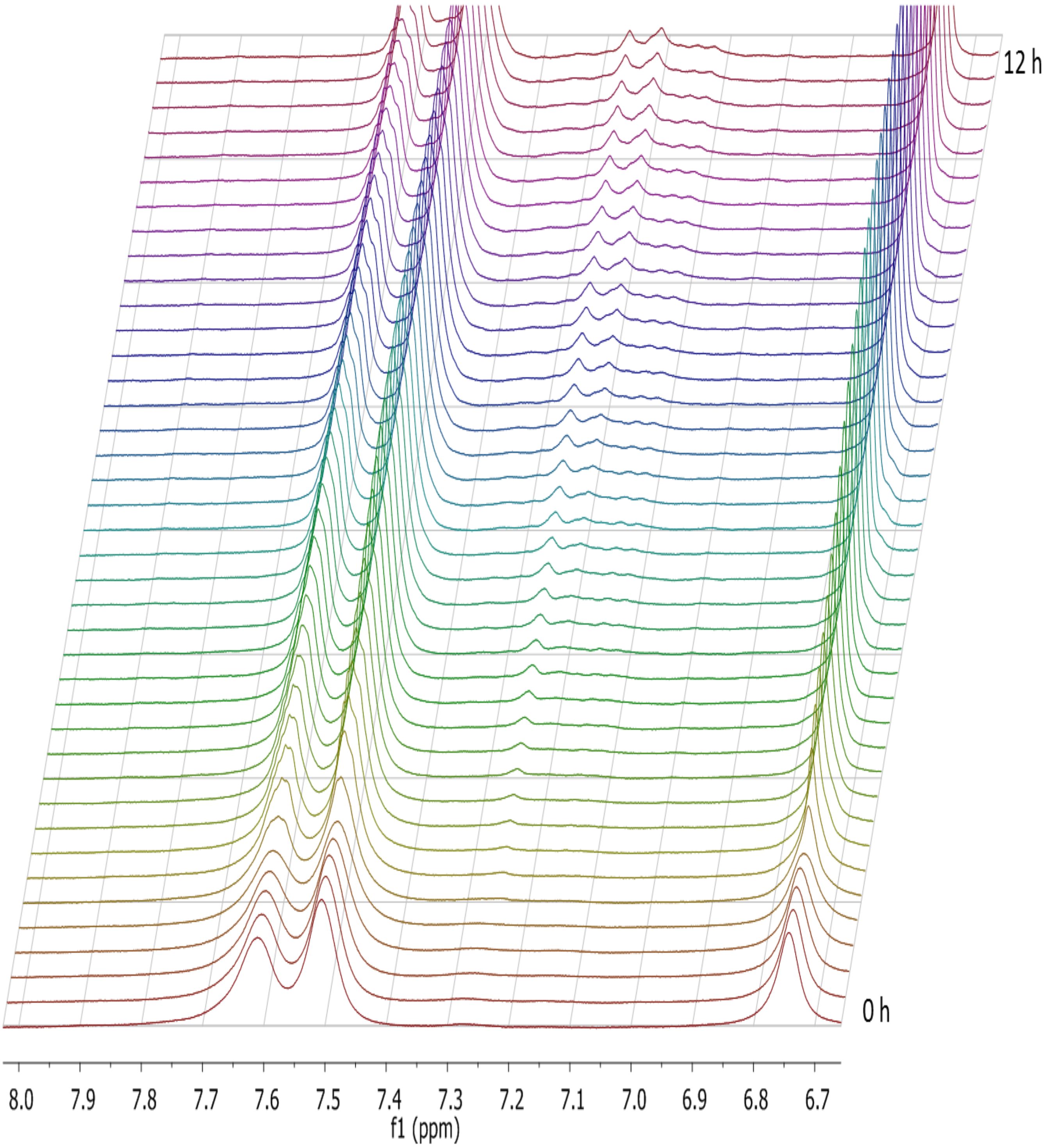

Supplementary Figure 20. Selected region of ^{1}H-NMR kinetics spectra of compound **10** in 0.5 M OH^{-} and λ=1 in DMSO-d_6 at room temperature. The aromatic peak of the internal standard mesitylene group appears at 6.7 ppm. The aromatic peaks of the compound at 7.5-7.6 ppm decrease while the degradation product peaks at 7.1 -7.2 ppm increase.

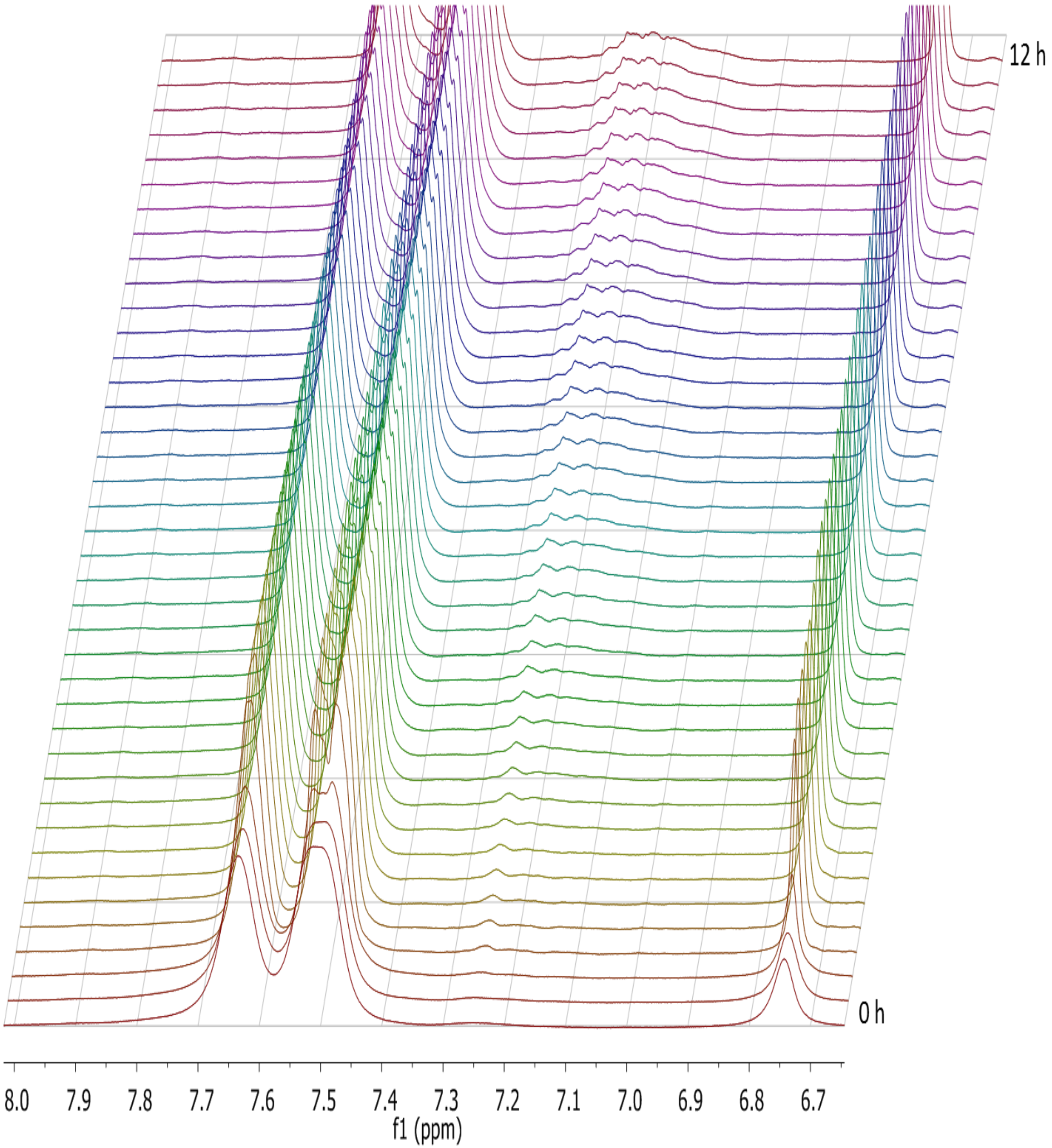

Supplementary Figure 21. Selected region of ^{1}H-NMR kinetics spectra of compound **11** in 0.5 M OH^{-} and λ=1 in DMSO-d_6 at room temperature. The aromatic peak of the internal standard mesitylene group appears at 6.7 ppm. The aromatic peaks of the compound at 7.5-7.6 ppm decrease while the degradation product peaks at 7.1 -7.2 ppm increase.

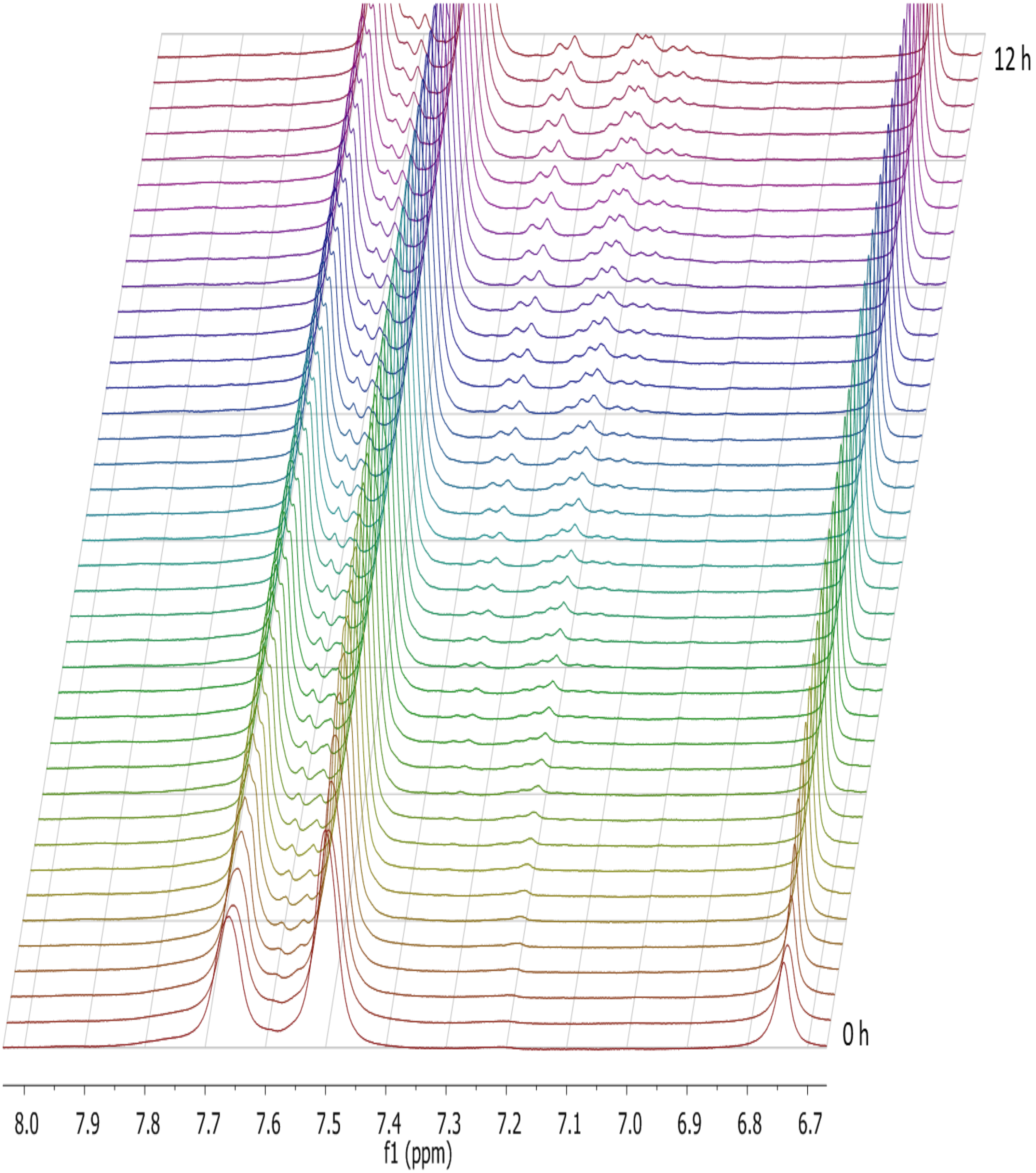

Supplementary Figure 22. Selected region of ^{1}H-NMR kinetics spectra of compound **12** in 0.5 M OH^- and λ=1 in DMSO-d_6 at room temperature. The aromatic peak of the internal standard mesitylene group appears at 6.7 ppm. The aromatic peaks of the compound at 7.5-7.6 ppm decrease while the degradation product peaks at 7.1 -7.2, 7.3-7.4 ppm increase.

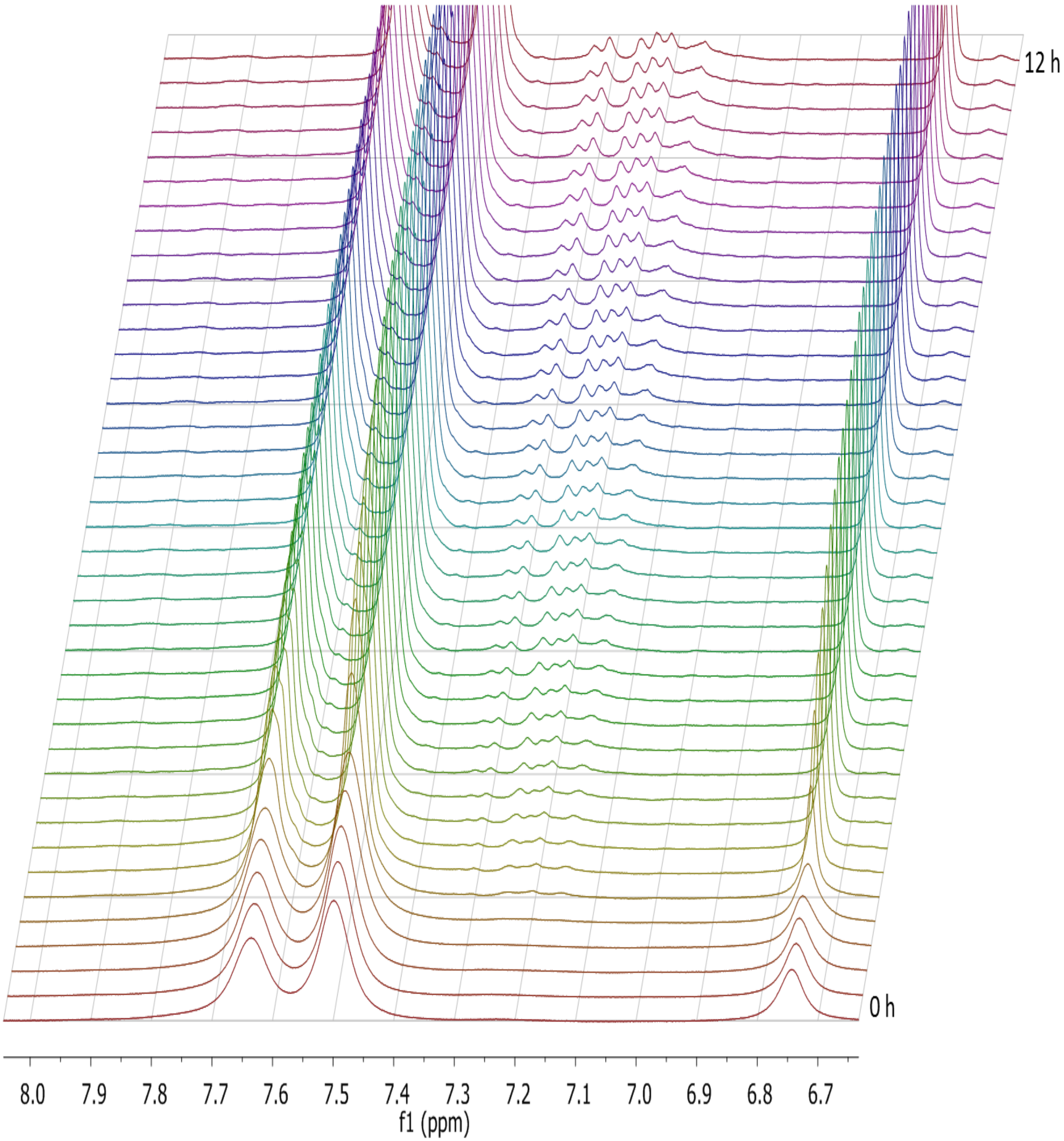

Supplementary Figure 23. Selected region of ^{1}H-NMR kinetics spectra of compound **13** in 0.5 M OH^{-} and λ=1 in DMSO-d_6 at room temperature. The aromatic peak of the internal standard mesitylene group appears at 6.7 ppm. The aromatic peaks of the compound at 7.5-7.6 ppm decrease while the degradation product peaks at 7.1 -7.3 ppm increase.

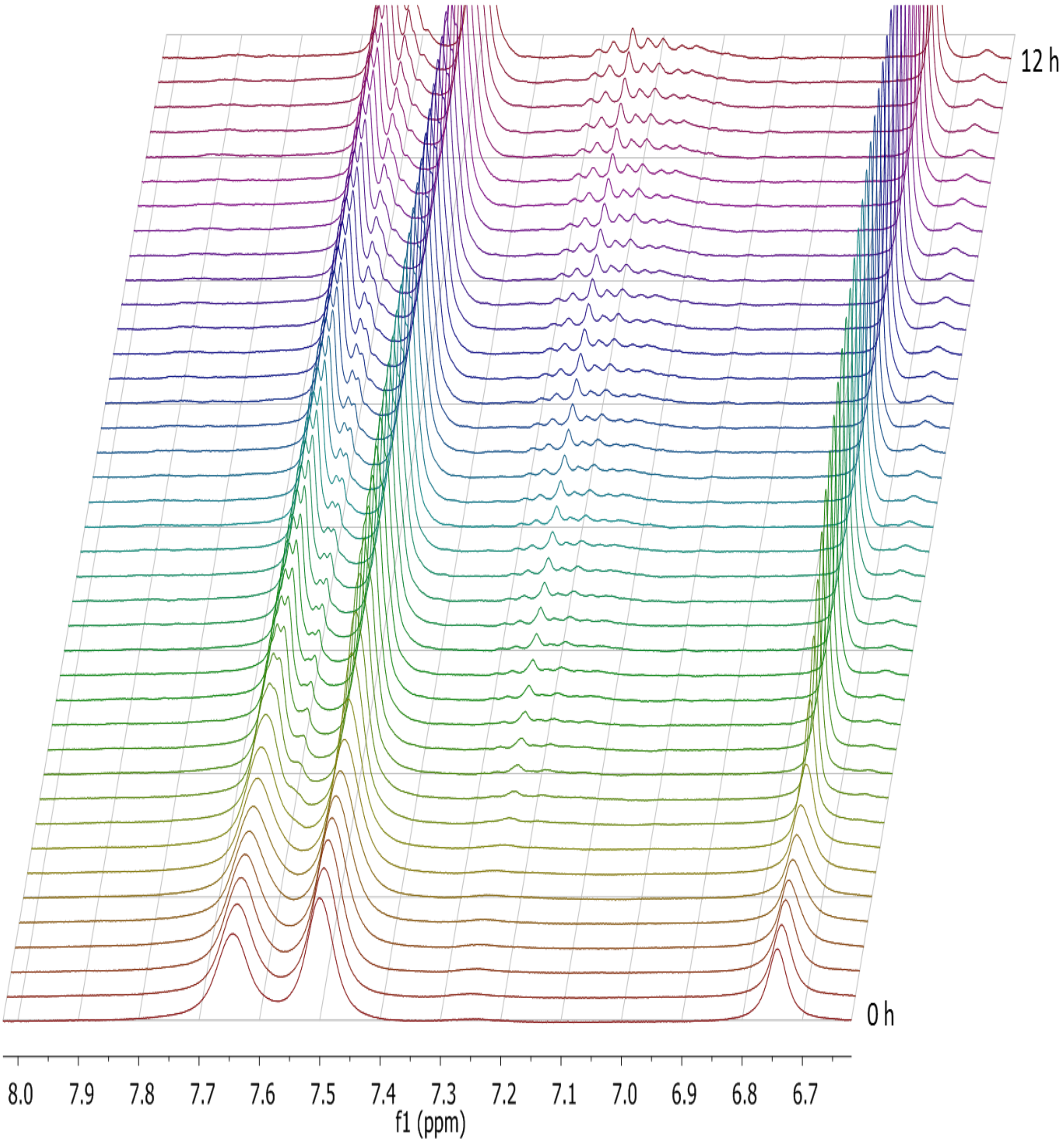

Supplementary Figure 24. Selected region of ^{1}H-NMR kinetics spectra of compound **14** in 0.5 M OH^- and λ=1 in DMSO-d_6 at room temperature. The aromatic peak of the internal standard mesitylene group appears at 6.7 ppm. The aromatic peaks of the compound at 7.5-7.6 ppm decrease while the degradation product peaks at 7.1 -7.3 ppm increase.

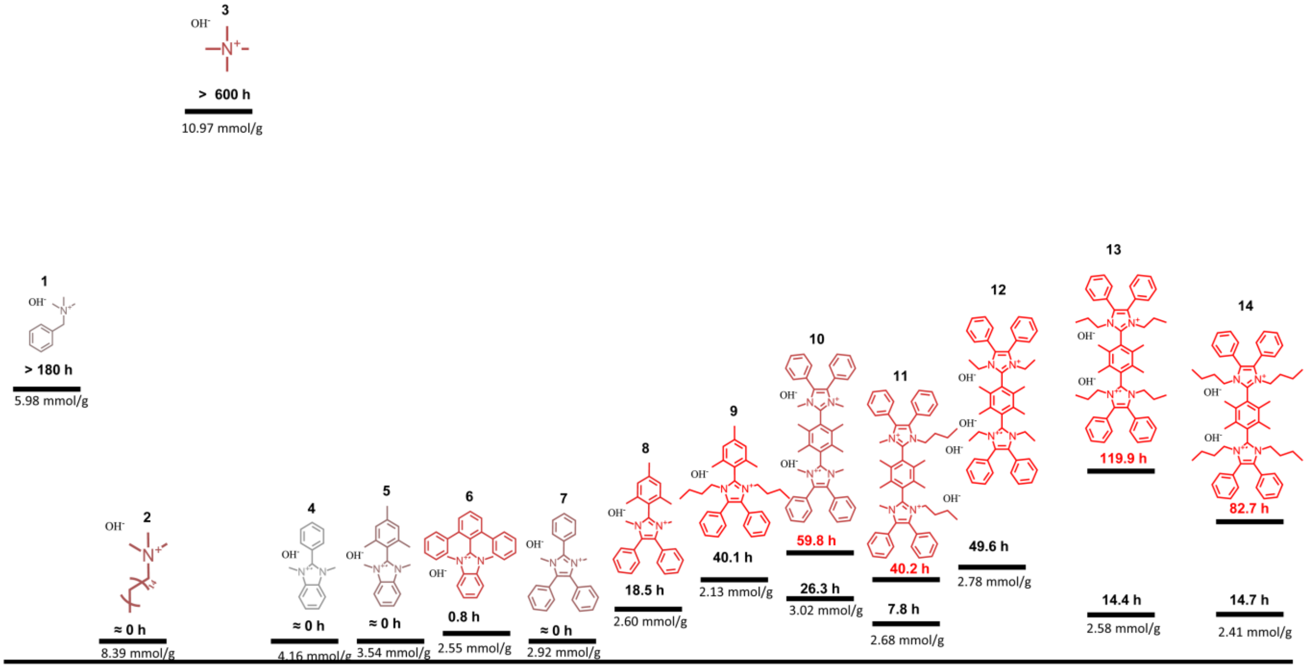

Supplementary Figure 25. Half life of cationic compounds in their hydroxide ion form under λ = 1 conditions. Half-life of bis-imidazoliums, which exhibit two stage degradation, are colored red.

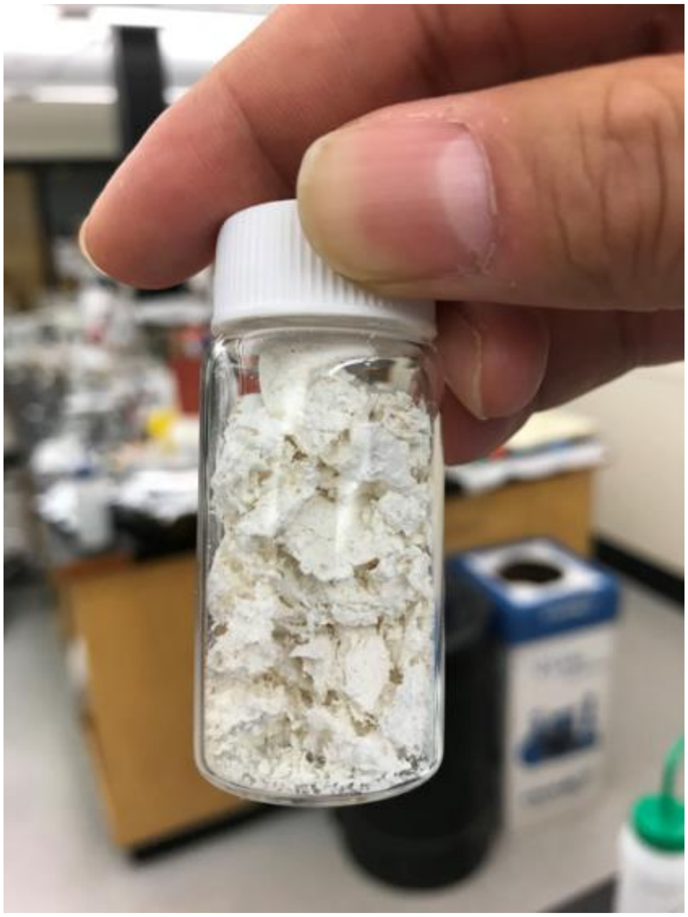

Supplementary Figure 26. Photograph of poly(bis-arylimidazole), precursor of PAImBB(**14**).

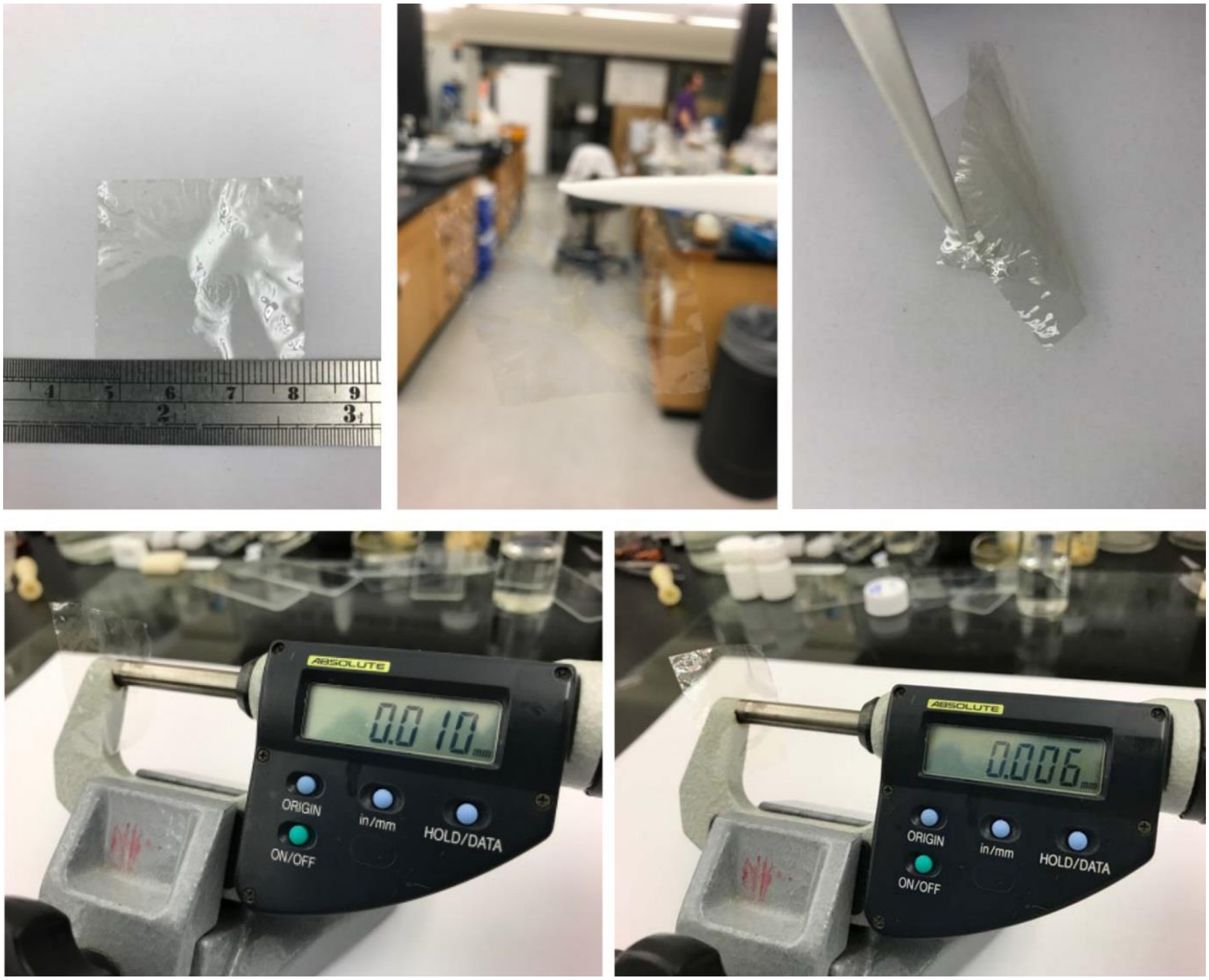

Supplementary Figure 27. Photographs of membrane of PAImBB(**14**).

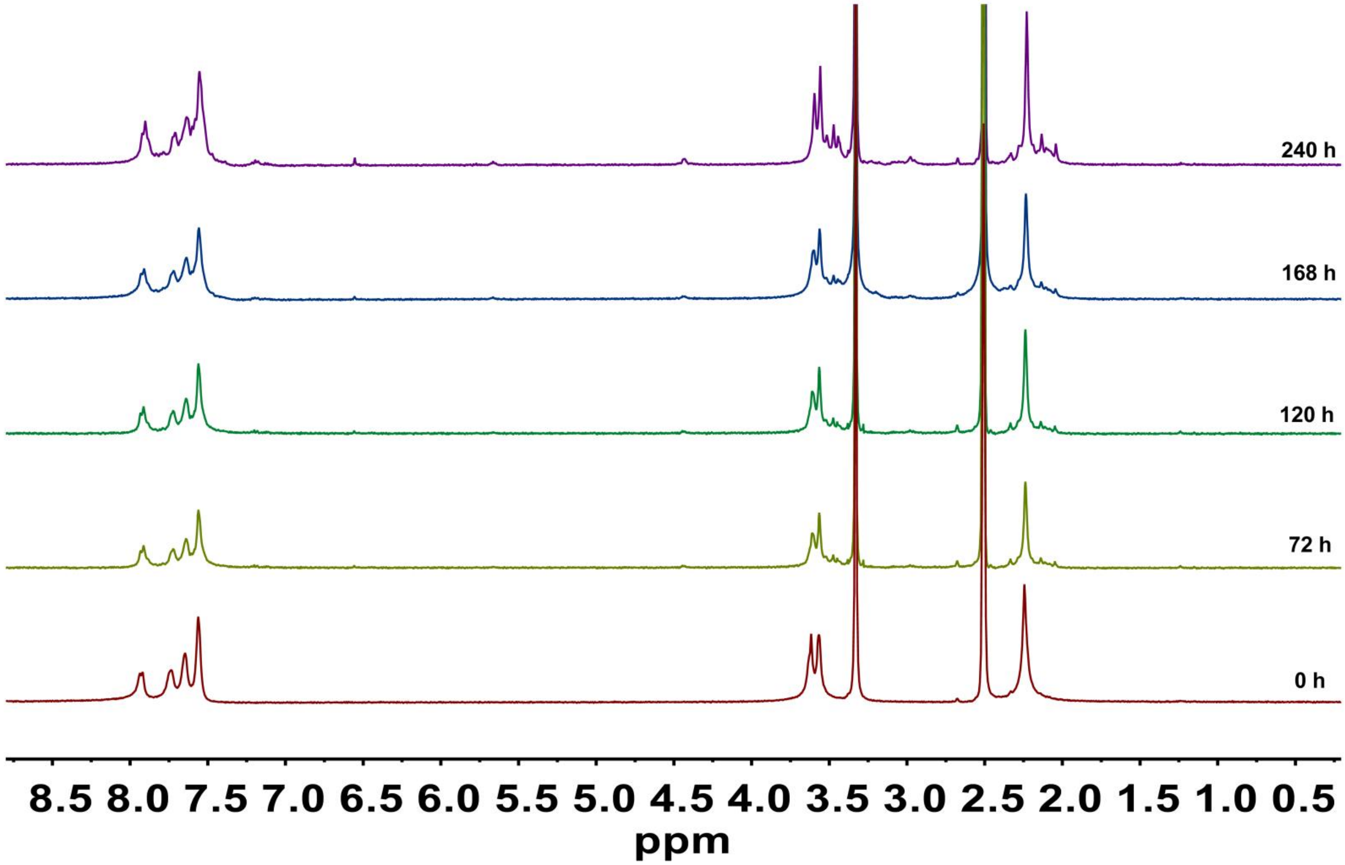

Supplementary Figure 28. The corresponding stacked solution ^{1}H NMR spectra (400 MHz, DMSO-d6) of PAImMM(**10**) in chloride form after membranes were soaked in 10 M KOH for various times (as labeled).

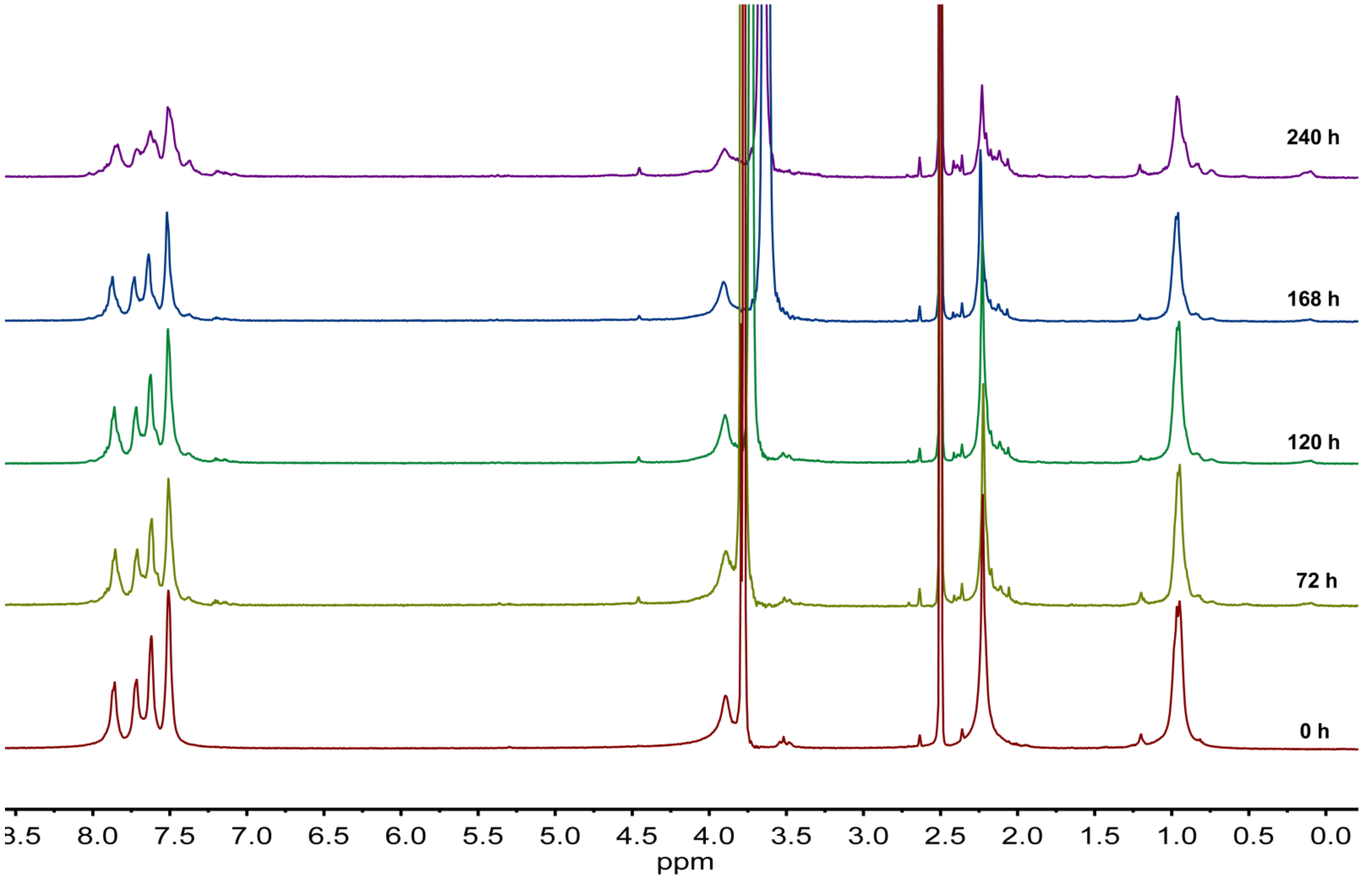

Supplementary Figure 29. The corresponding stacked solution ^{1}H NMR spectra (400 MHz, DMSO-d6) of PAImEE(**12**) in chloride form after membranes were soaked in 10 M KOH for various times (as labeled).

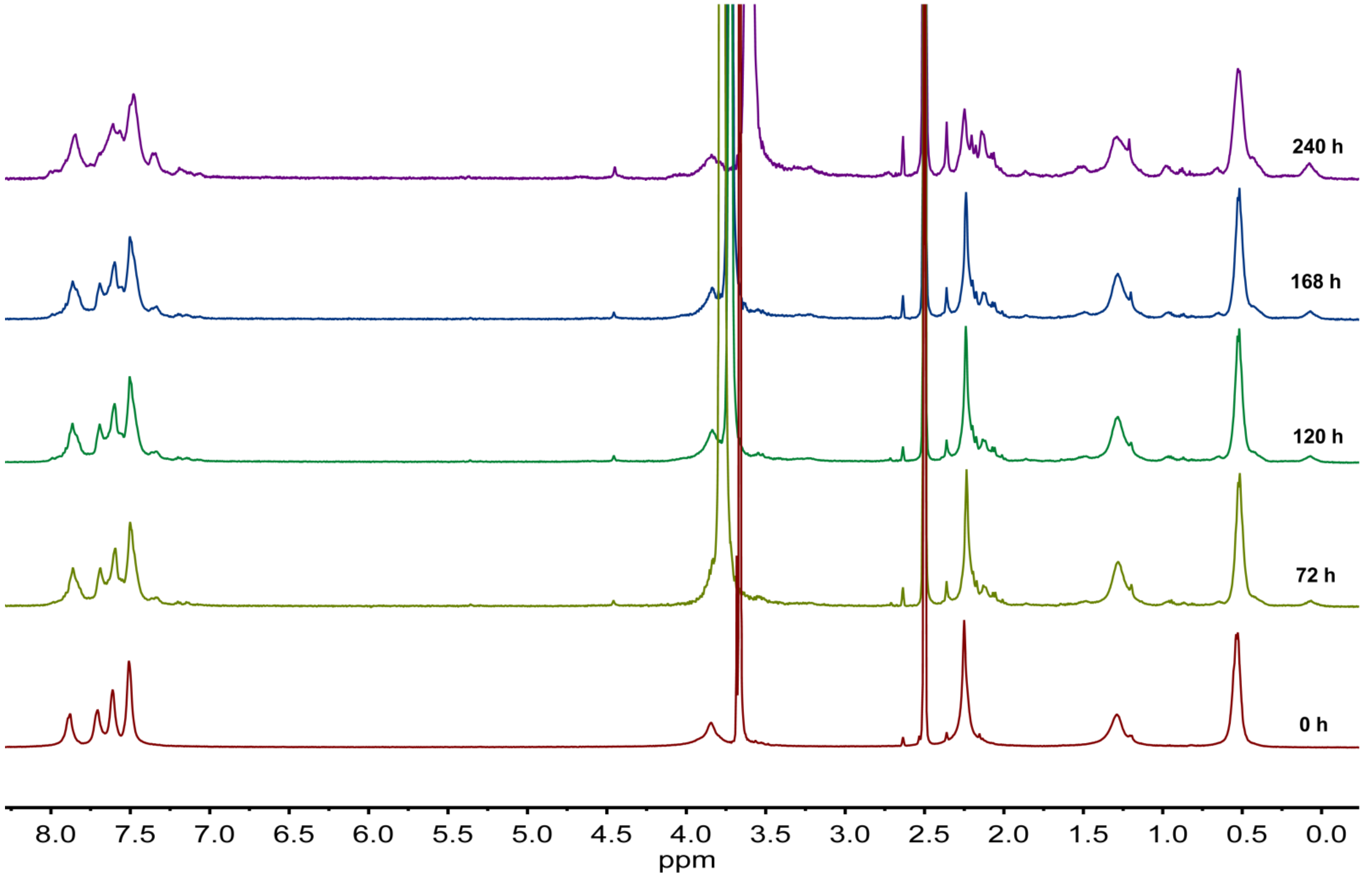

Supplementary Figure 30. The corresponding stacked solution ^{1}H NMR spectra (400 MHz, DMSO-d6) of PAImPP(**13**) in chloride form after membranes were soaked in 10 M KOH for various times (as labeled).

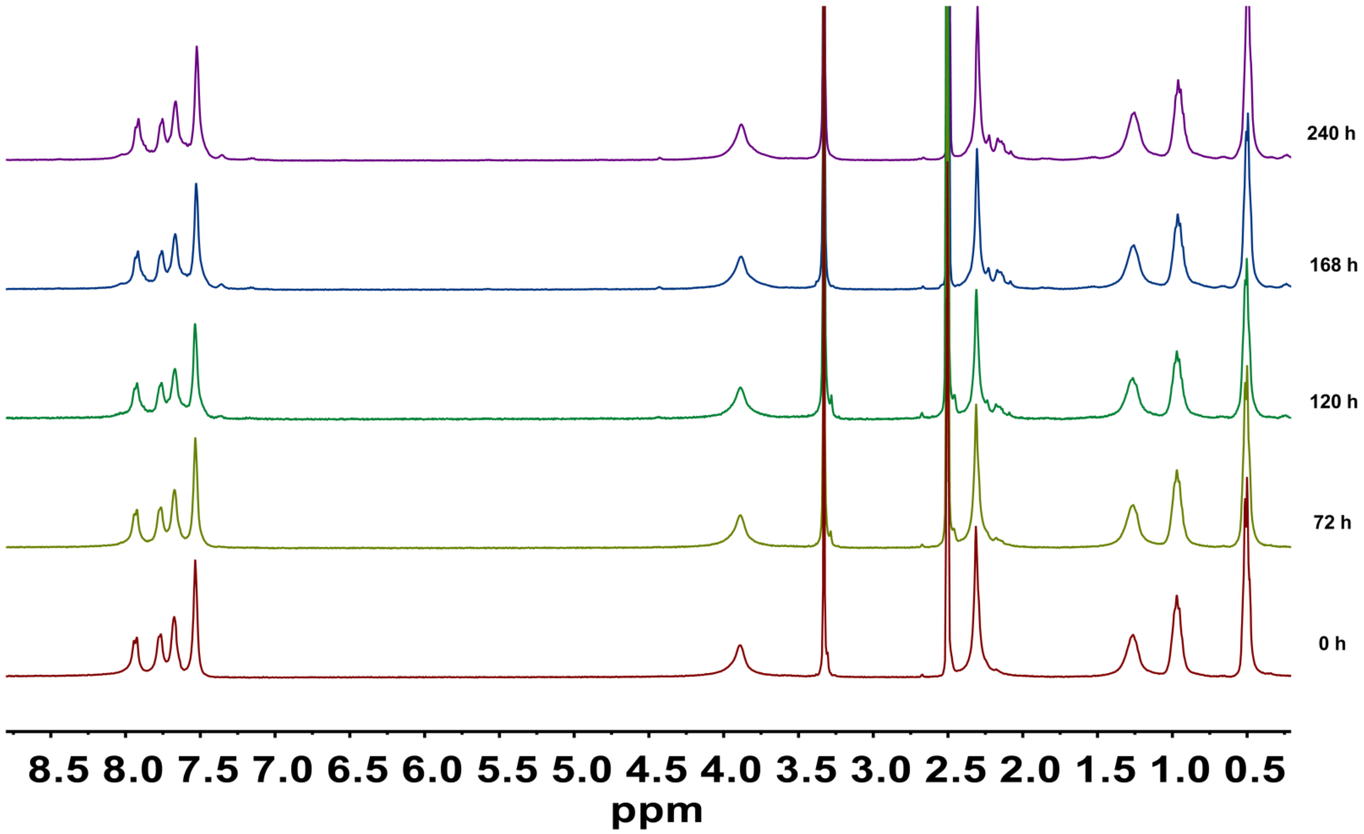

Supplementary Figure 31. The corresponding stacked solution ^{1}H NMR spectra (400 MHz, DMSO-d6) of PAImBB(**14**) in chloride form after membranes were soaked in 10 M KOH for various times (as labeled).

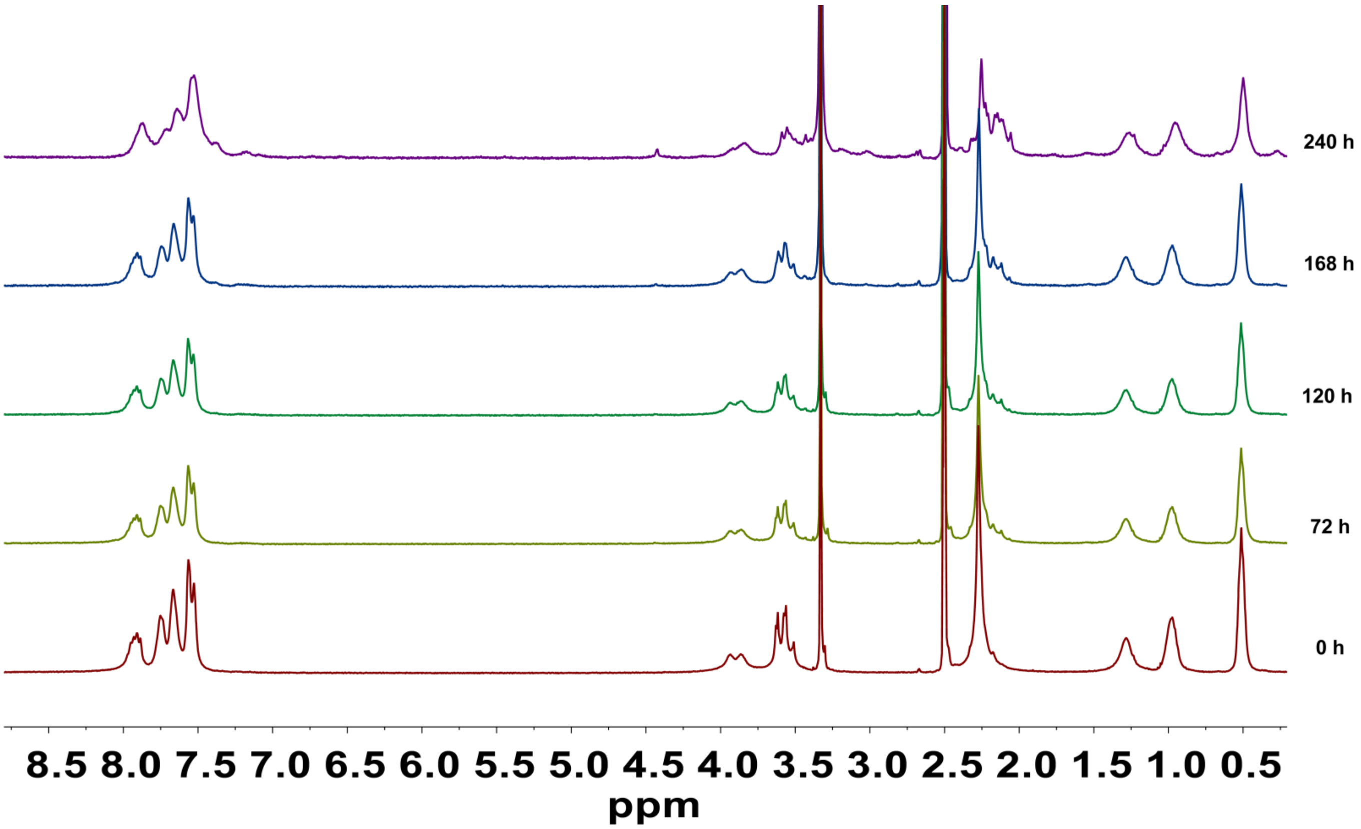

Supplementary Figure 32. The corresponding stacked solution ^{1}H NMR spectra (400 MHz, DMSO-d6) of PAImMB(**11**) in chloride form after membranes were soaked in 10 M KOH for various times (as labeled).

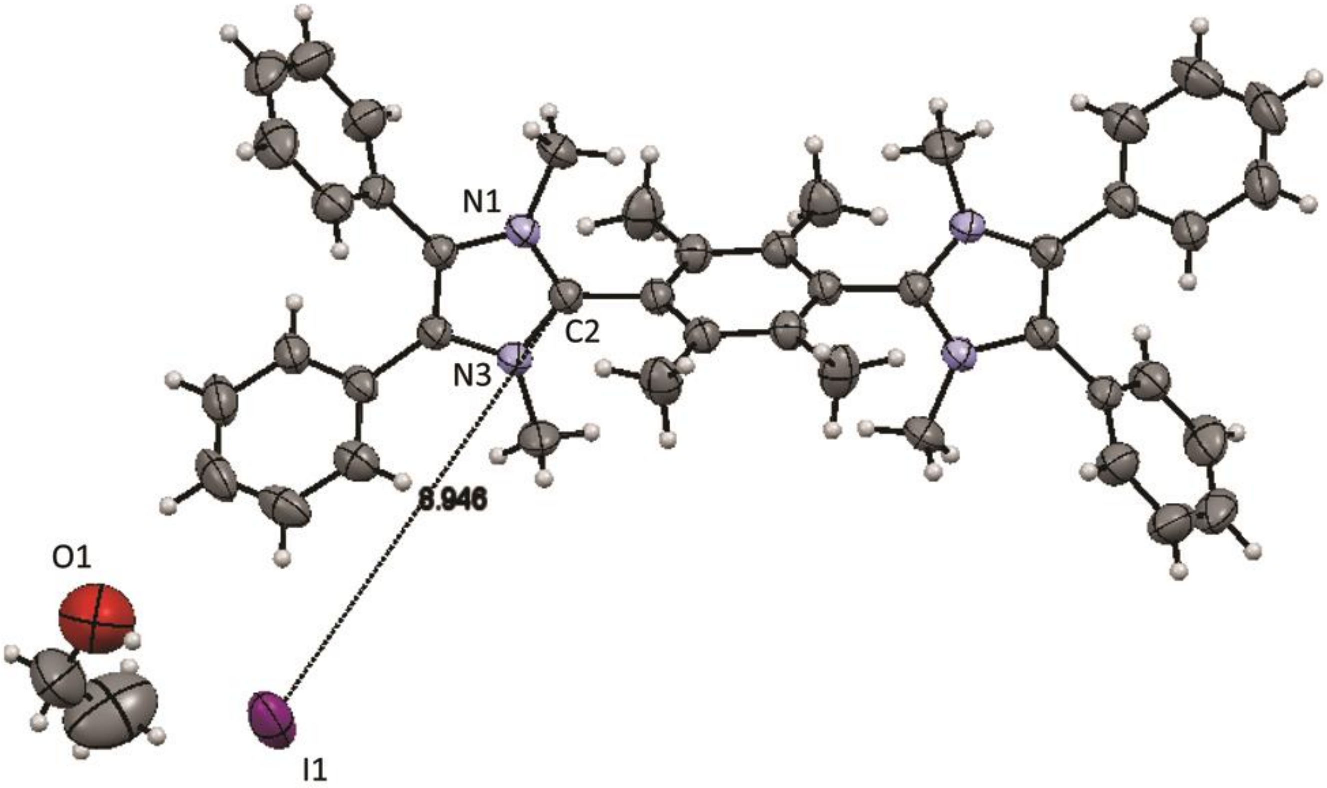

Supplementary Figure 33. X-ray crystal structure of **10** in its iodide form (ellipsoid set at 50% probability) with distance from iodide to C2, crystallized from ethanol.

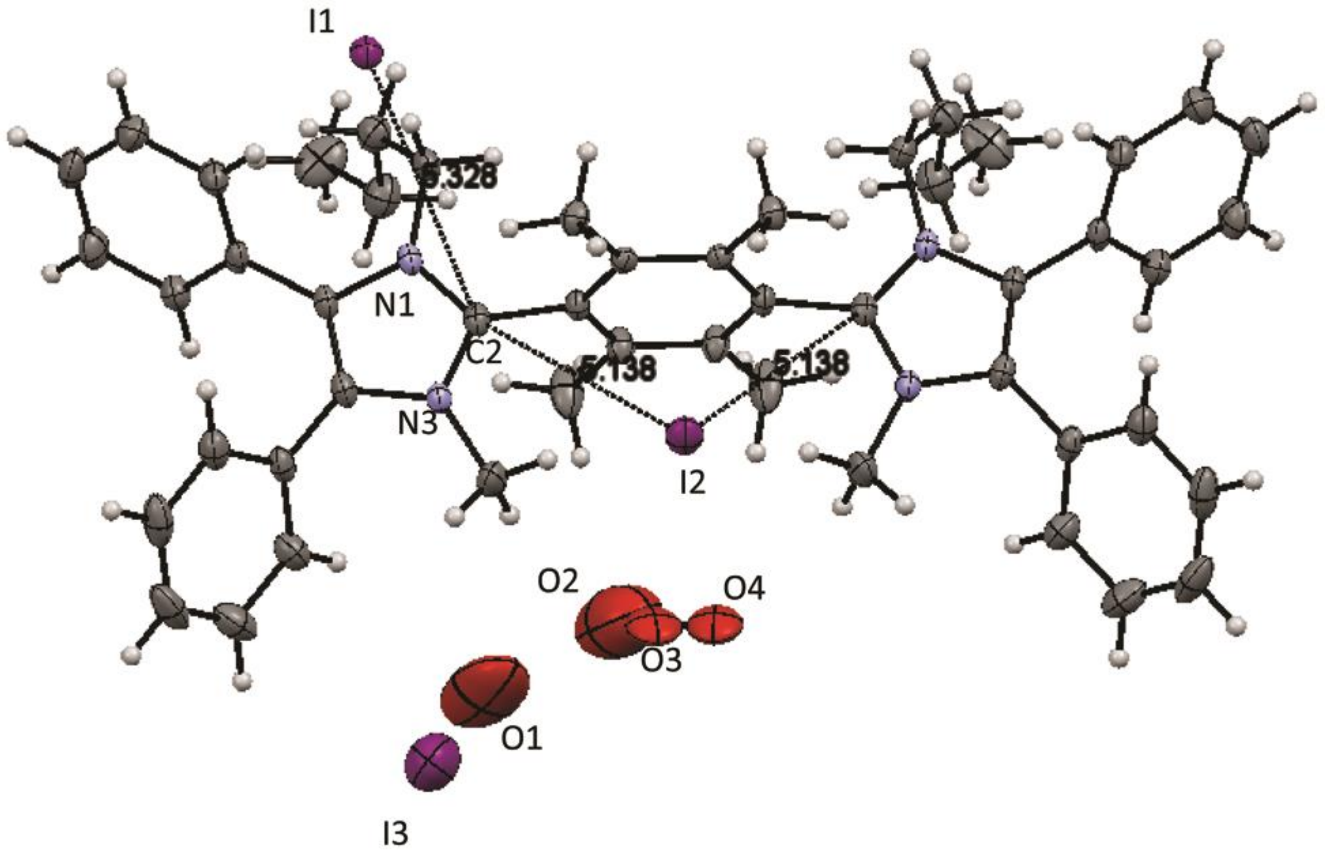

Supplementary Figure 34. X-ray crystal structure of **11** in its iodide form (ellipsoid set at 50% probability) with distance from iodide to C2, crystallized from IPA and water

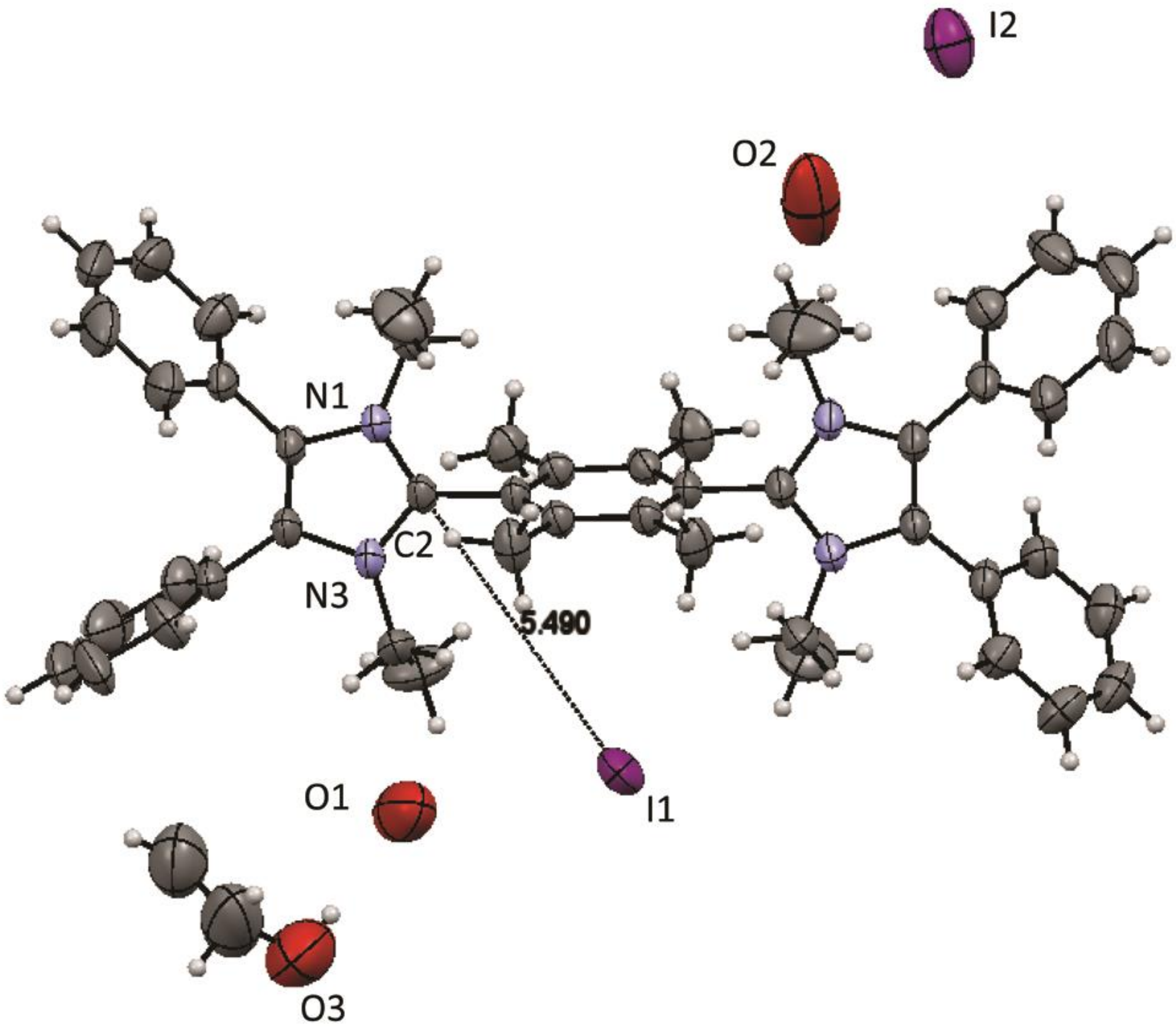

Supplementary Figure 35. X-ray crystal structure of model compound **12** in its iodide form (ellipsoid set at 50% probability) with distance from iodide to C2, crystallized from ethanol and water.

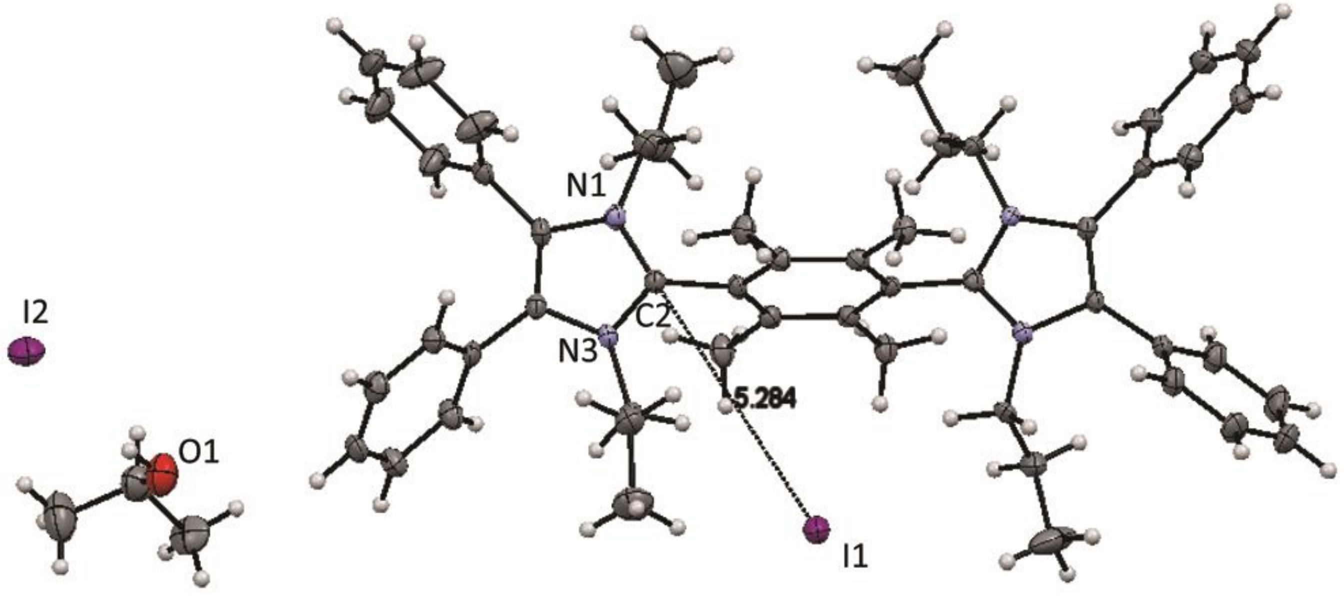

Supplementary Figure 36. X-ray crystal structure of model compound **13** in its iodide form (ellipsoid set at 50% probability) with distance from iodide to C2, crystallized from IPA.

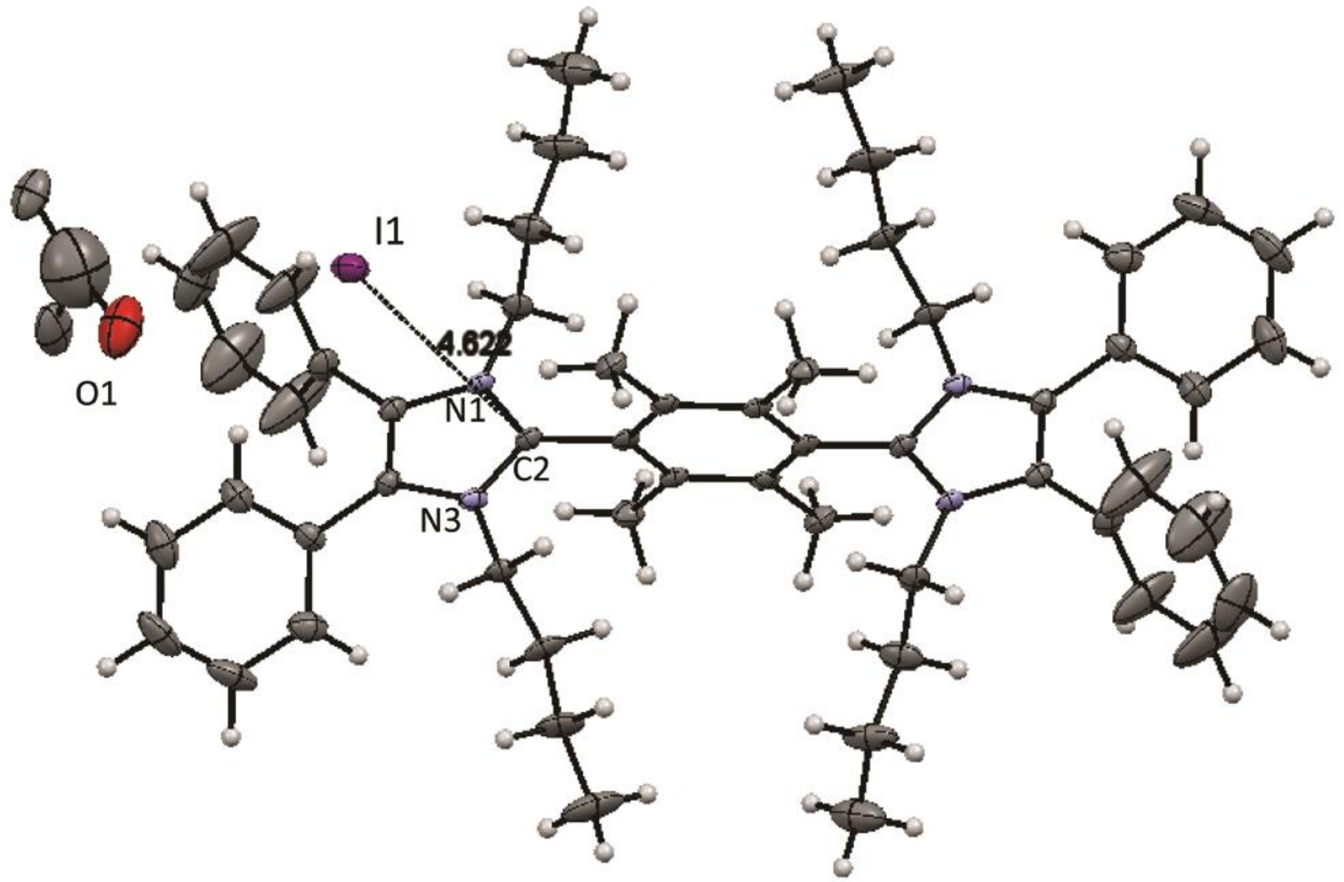

Supplementary Figure 37. X-ray crystal structure of model compound **14** in its iodide form (ellipsoid set at 50% probability) with distance from iodide to C2, crystallized from IPA.

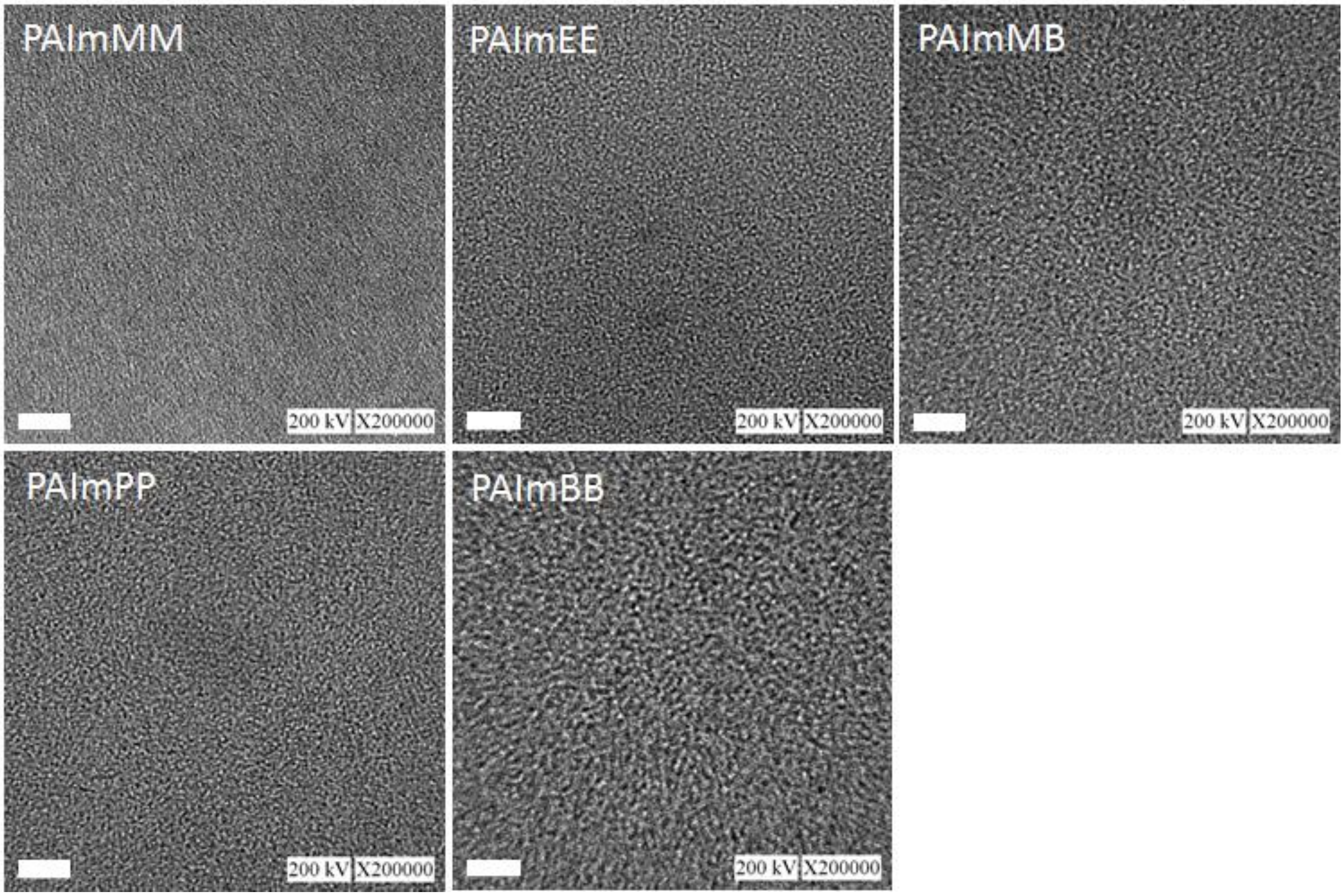

Supplementary Figure 38. Transmission electron micrographs of PAImXY(#) in their as-cast iodide form, scale bar represent 10 nm.

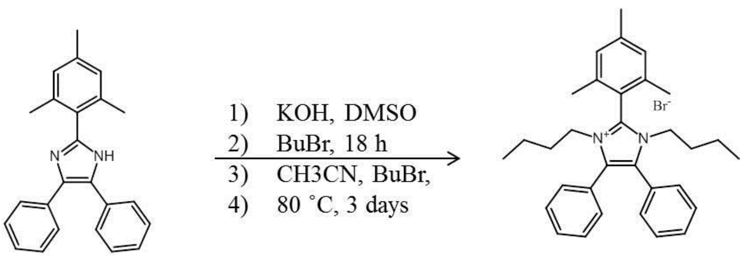

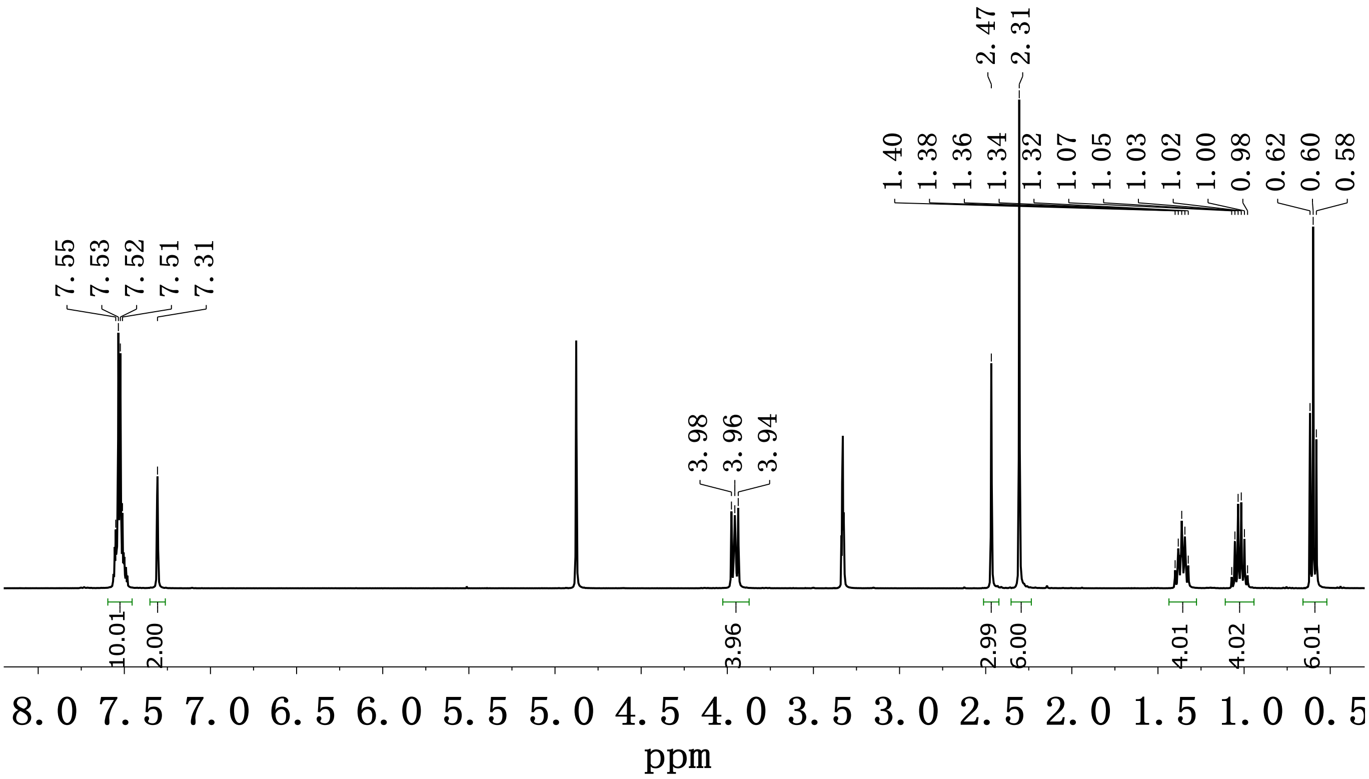

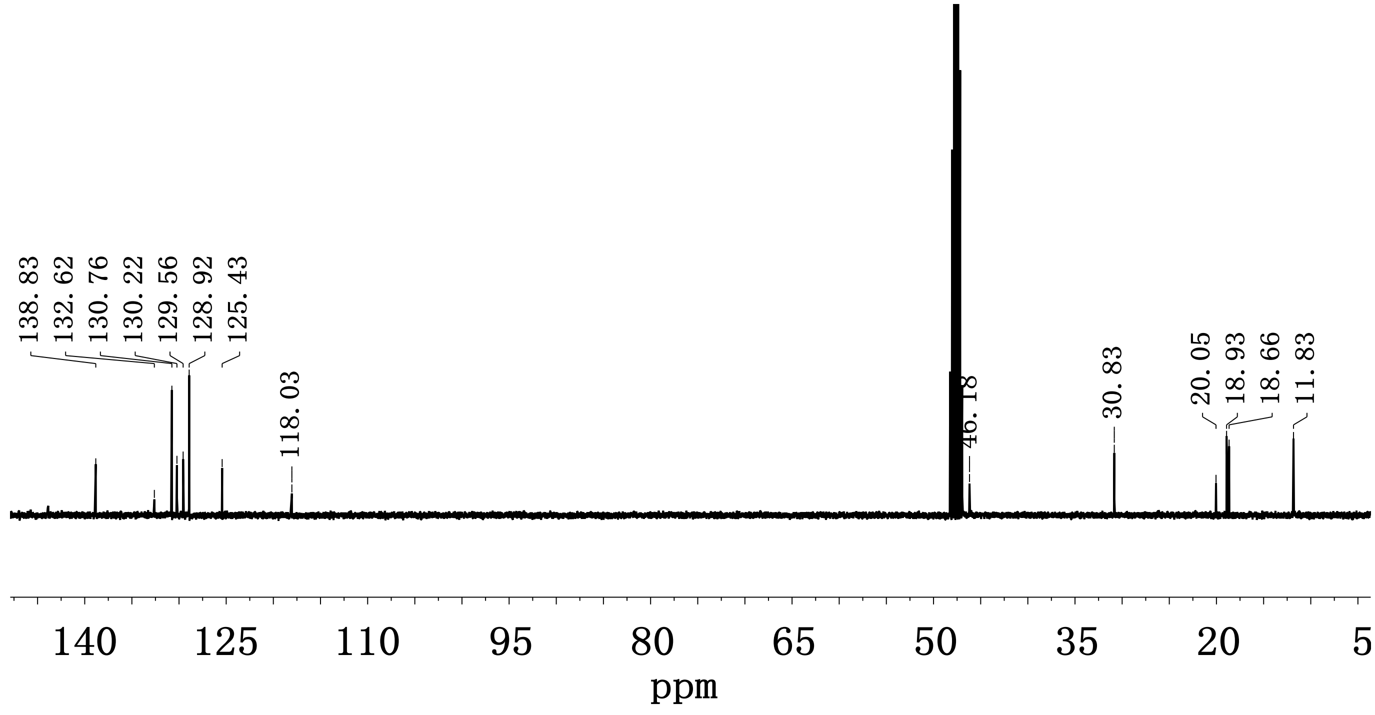

Supplementary Figure 39. Synthesis of compound **9** with ^{1}H and ^{13}C NMR of compound **9** in methanol-*d*4.

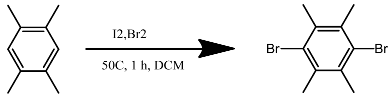

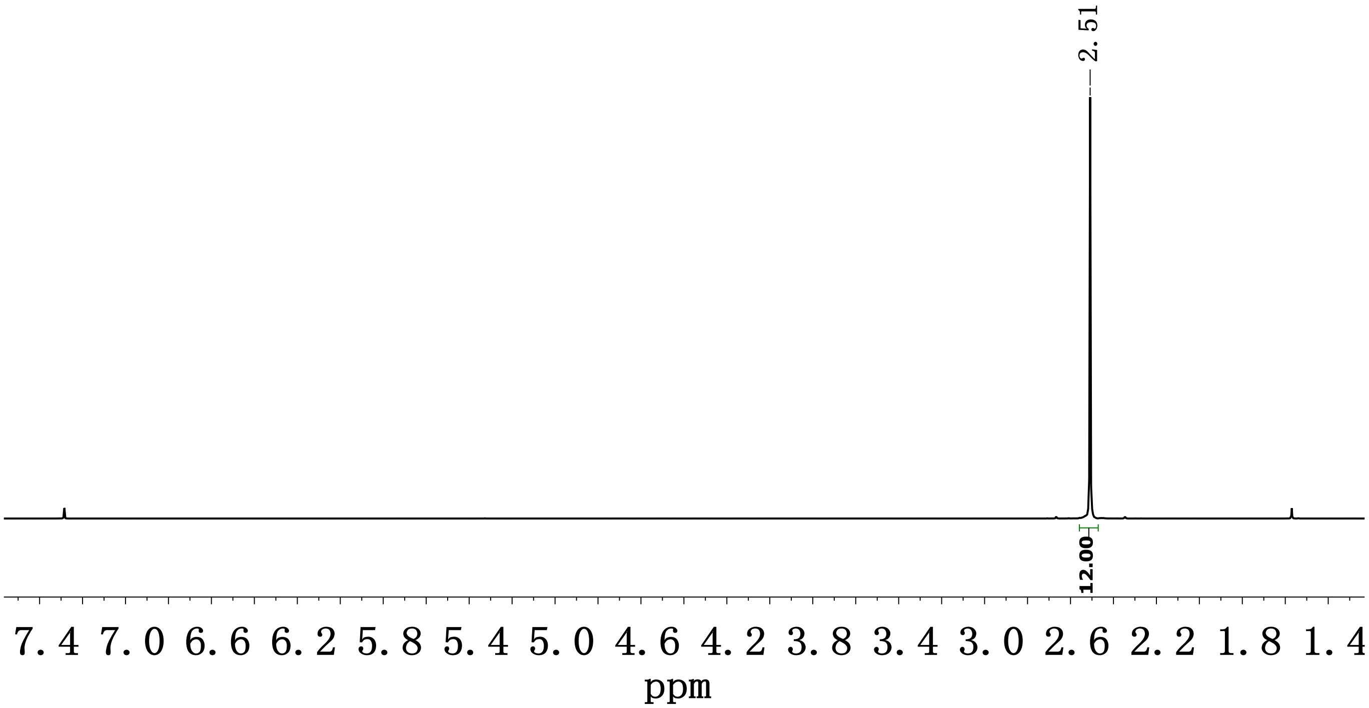

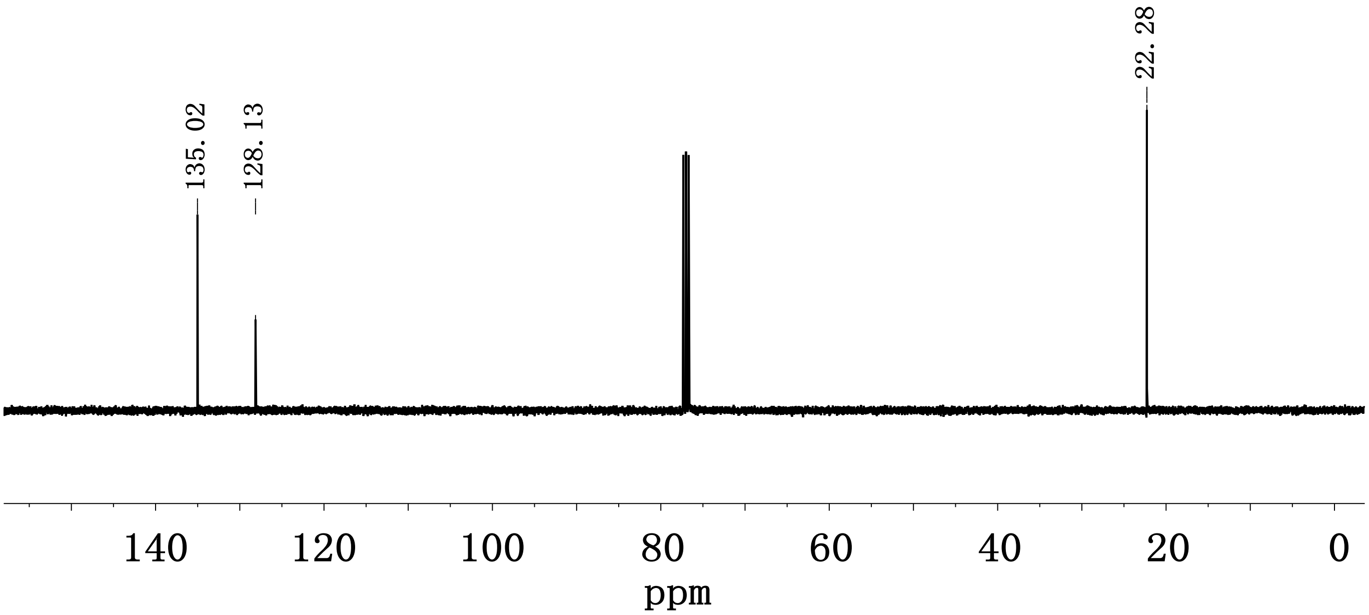

Supplementary Figure 40. Synthesis of 1,4-dibromo-2,3,5,6-tetramethylbenzene with ^{1}H and ^{13}C NMR of it in CD_2Cl_2.

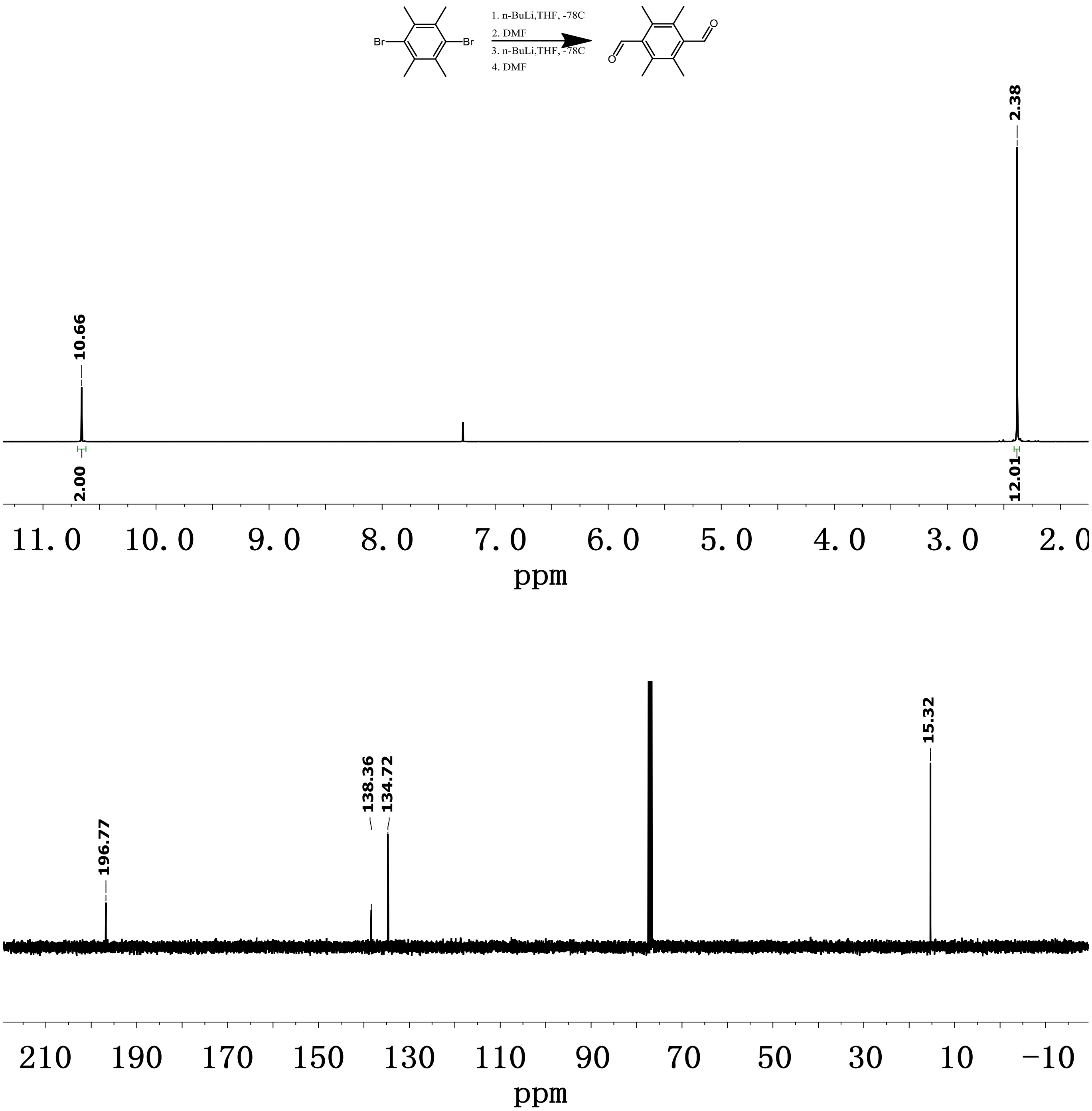

Supplementary Figure 41. Synthesis of 1,4-dialdehyde-2,3,5,6-tetramethylbenzene with ^{1}H and ^{13}C NMR of it in CD_3Cl.

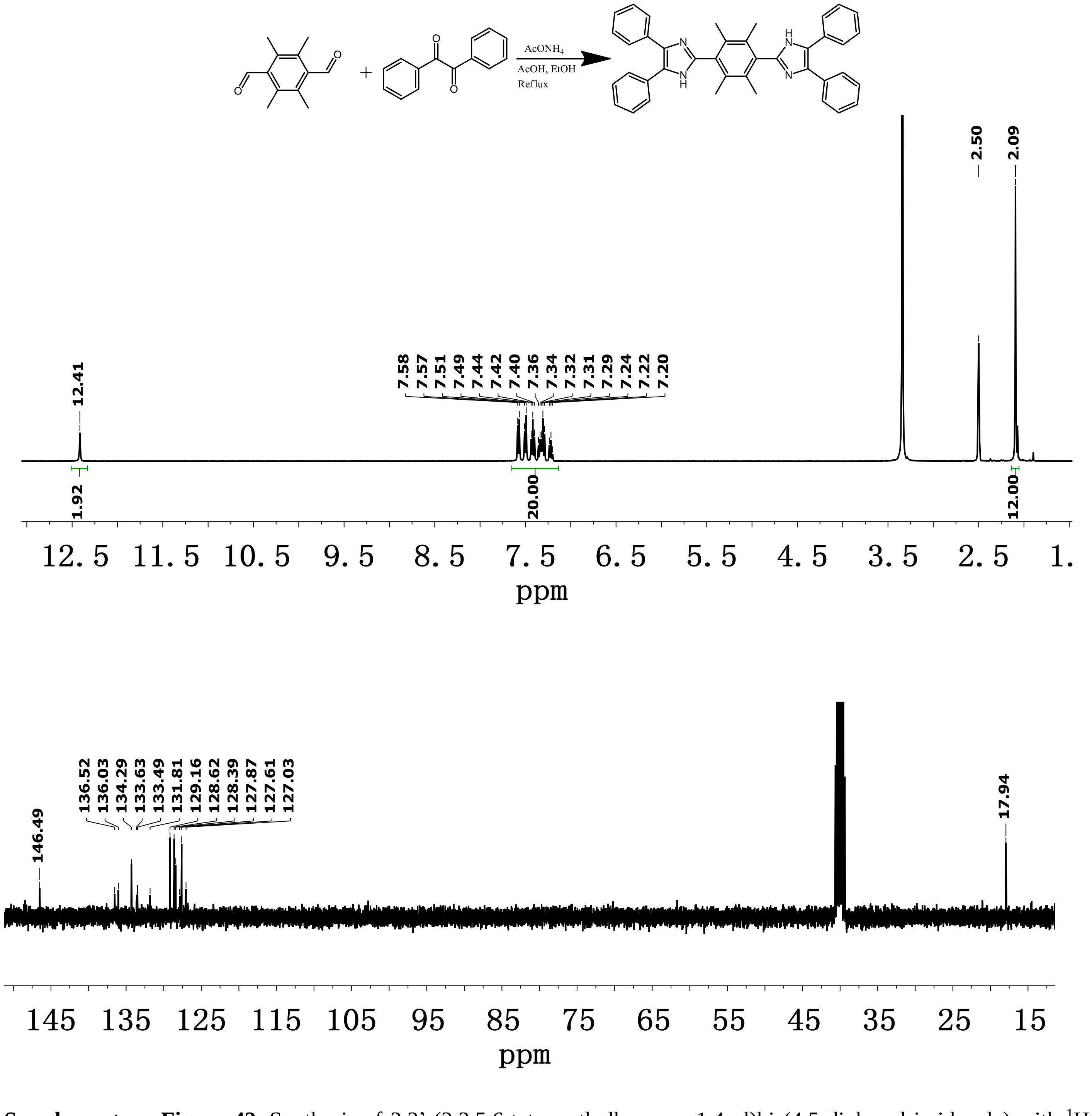

Supplementary Figure 42. Synthesis of 2,2'-(2,3,5,6-tetramethylbenzene-1,4-yl)bis(4,5-diphenyl-imidazole) with ^{1}H and ^{13}C NMR of it in DMSO-*d*6.

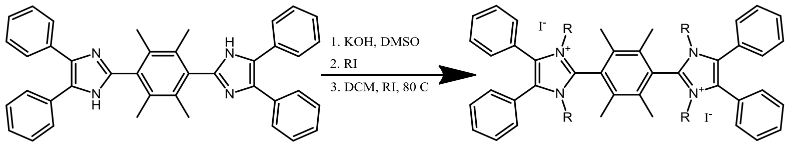

Supplementary Figure 43. Synthesis of compounds **10-14**.

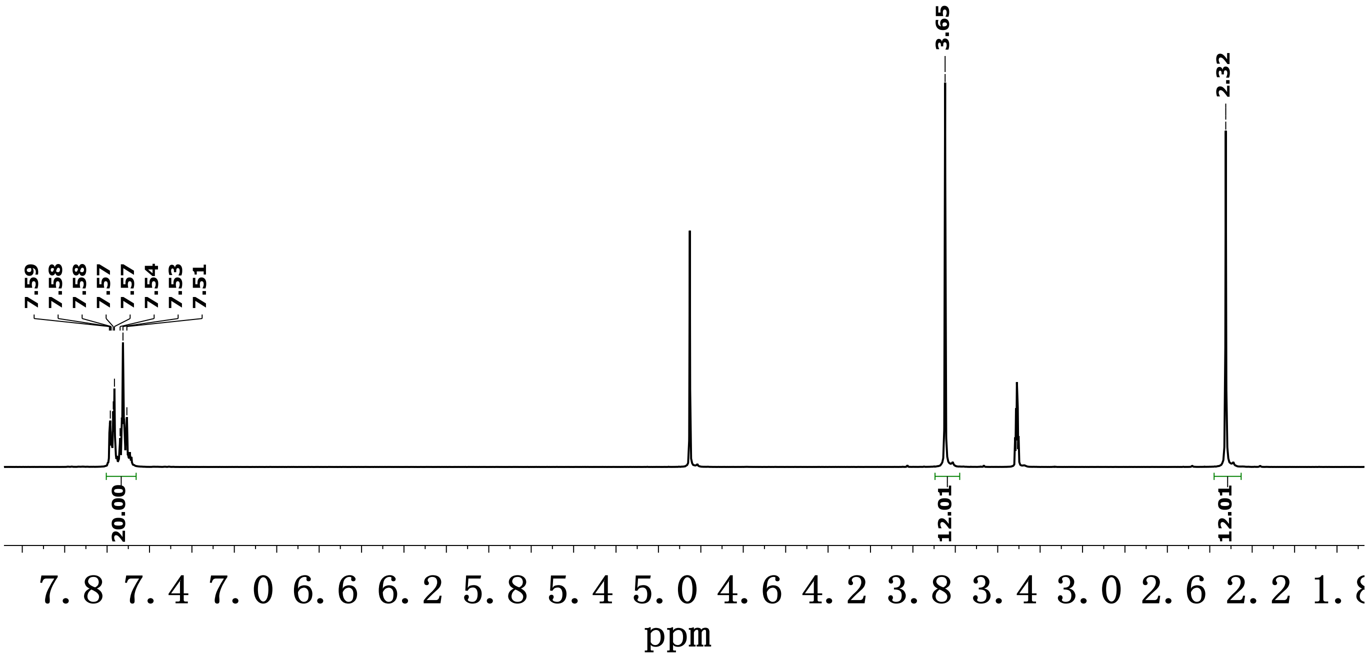

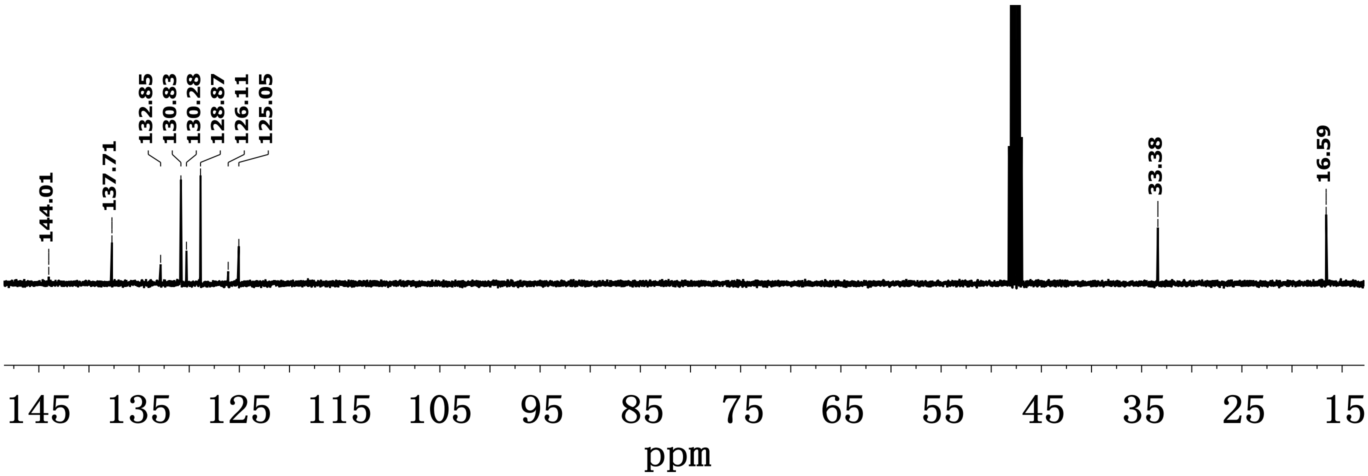

Supplementary Figure 44.^{1}H and ^{13}C NMR of **10** in methanol-*d*4.

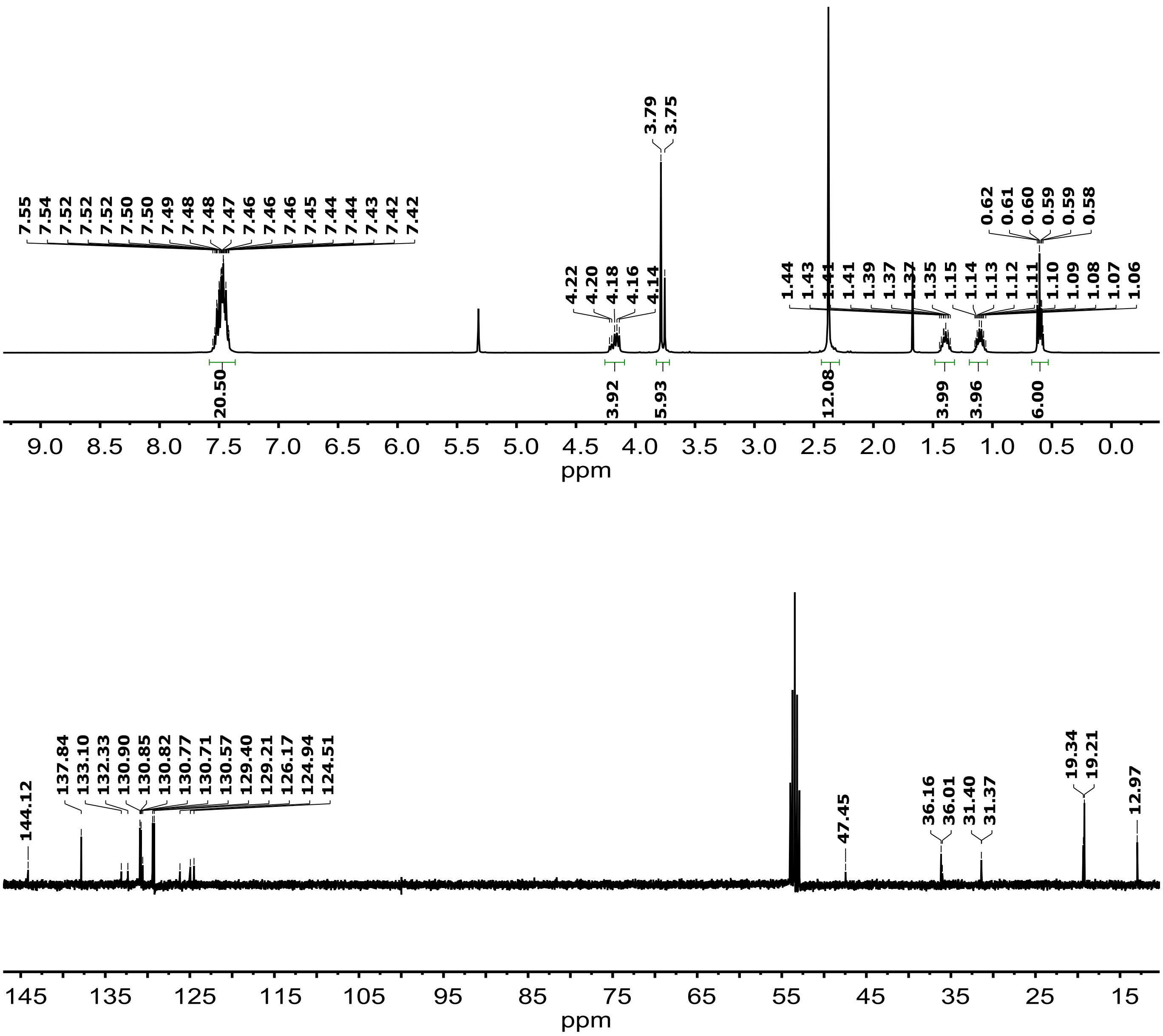

Supplementary Figure 45. ^{1}H and ^{13}C NMR of **11** in CD_2Cl_2.

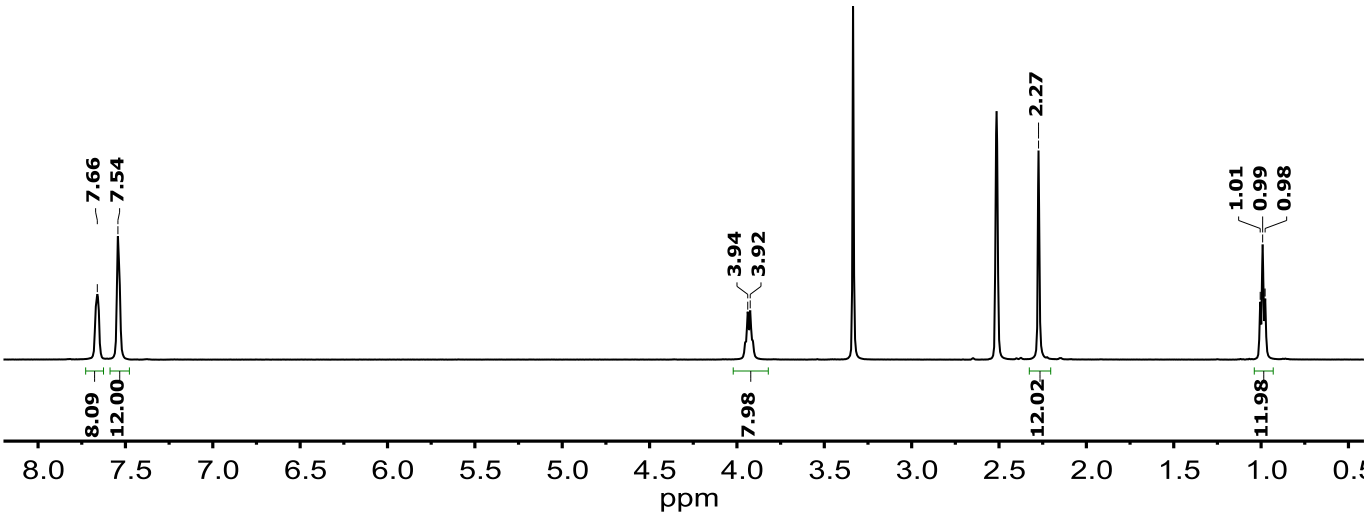

Supplementary Figure 46. ^{1}H NMR of **12** in DMSO-*d6*.

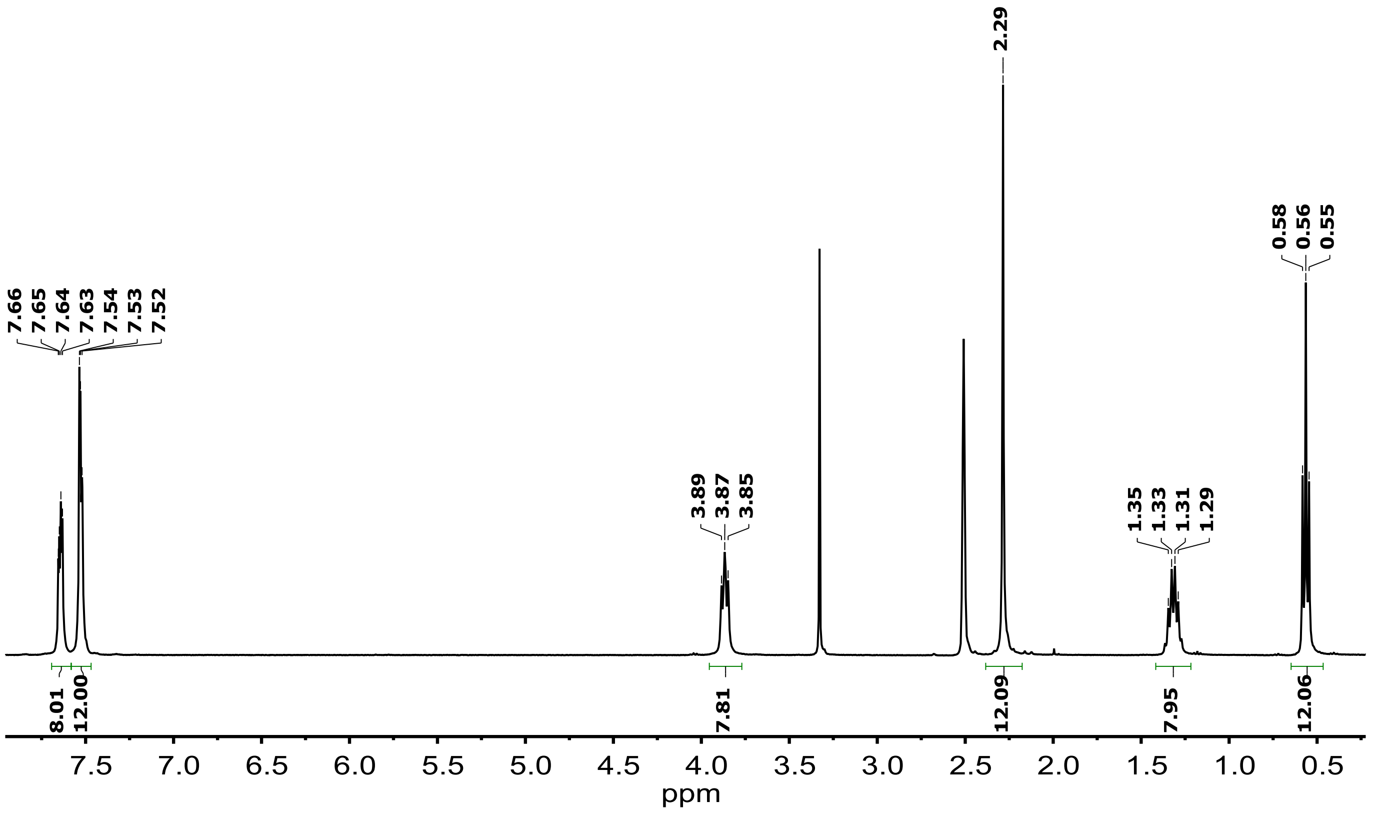

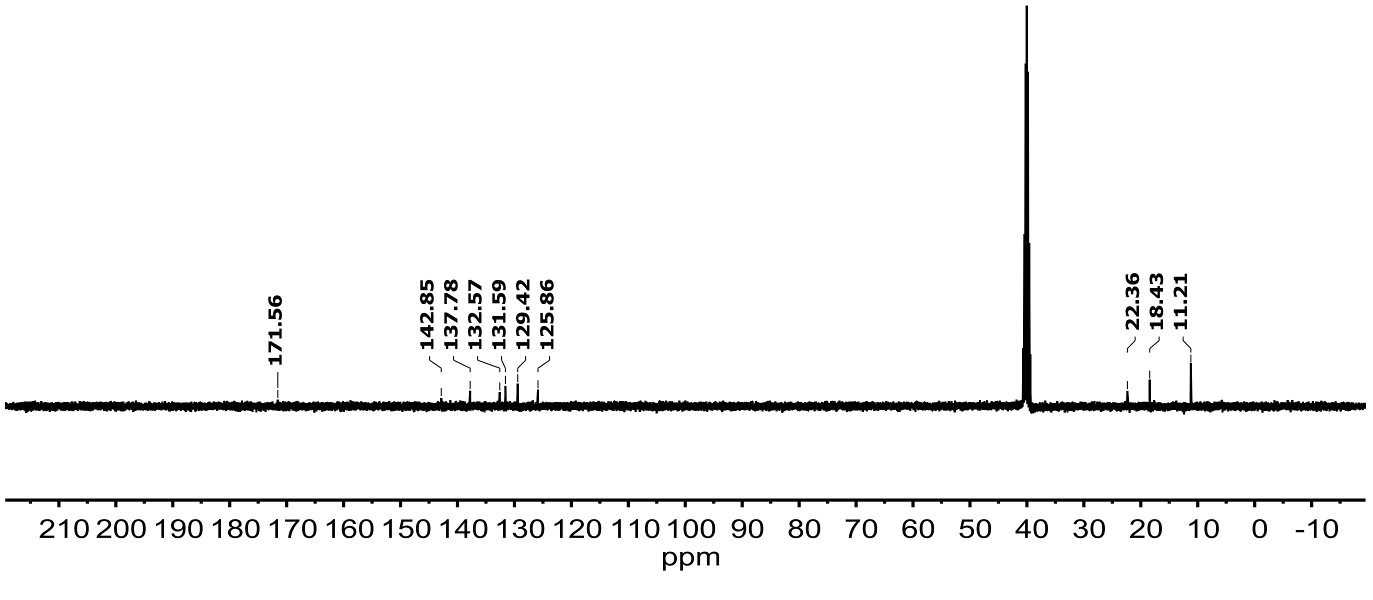

Supplementary Figure 47. ^{1}H and ^{13}C NMR of **13** in DMSO-*d6*.

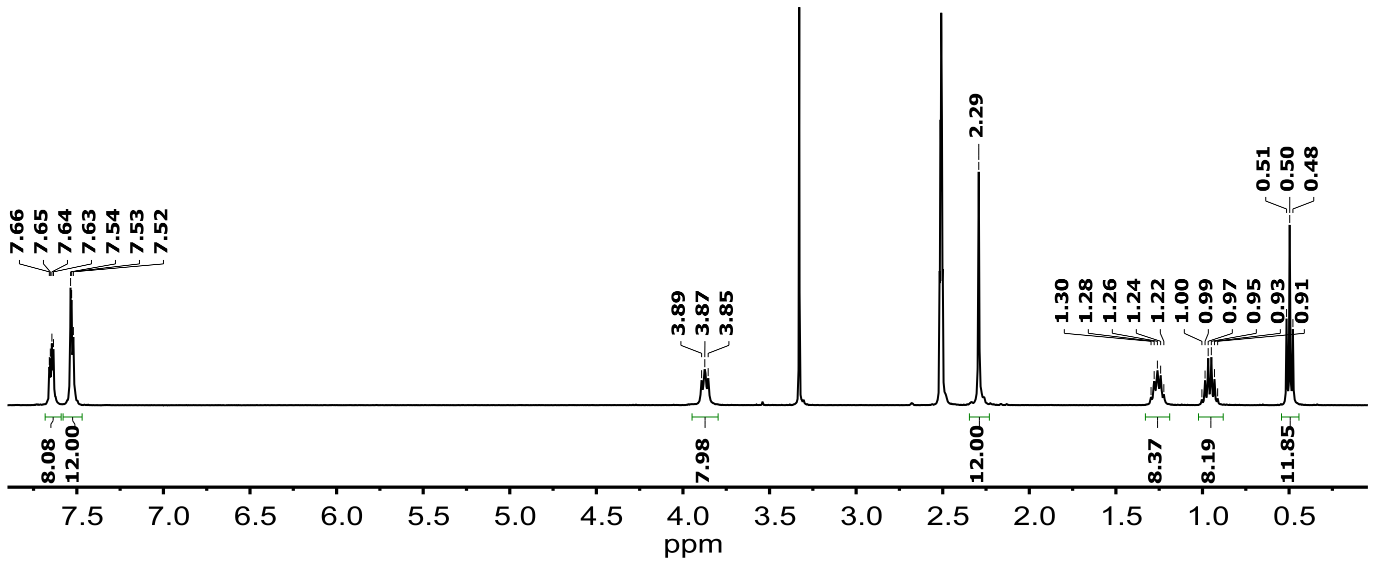

Supplementary Figure 48. ^{1}H NMR of **14** in DMSO-*d6*.

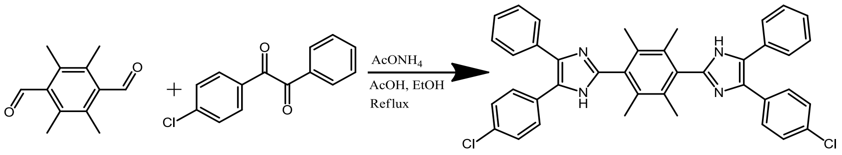

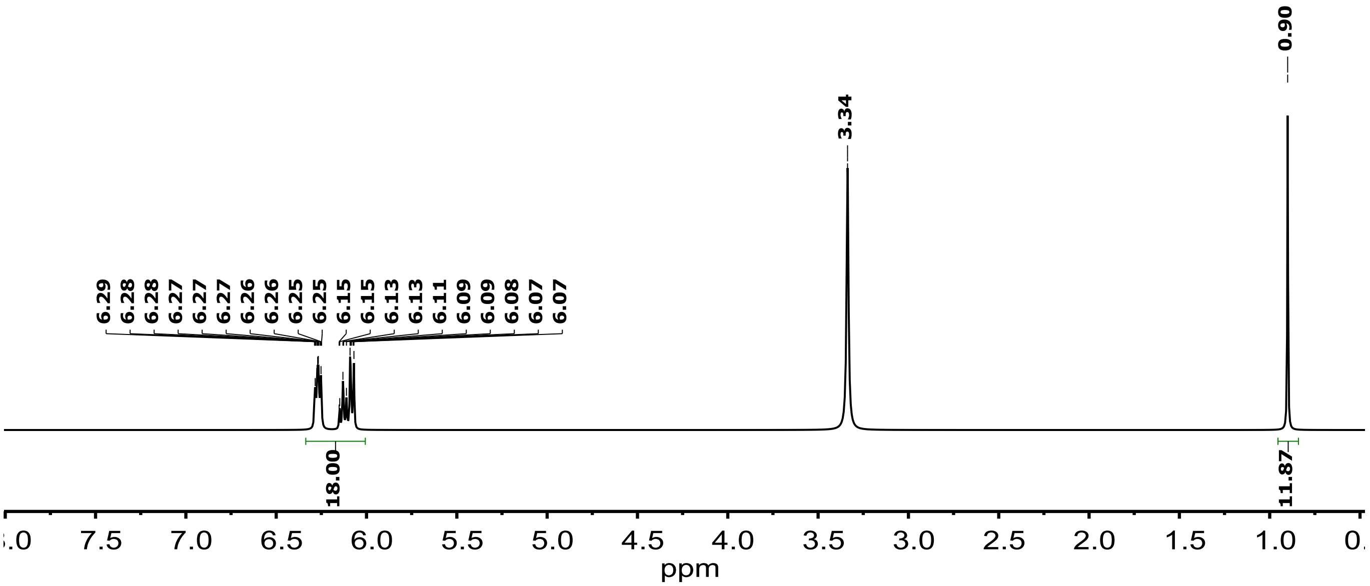

Supplementary Figure 49. Synthesis of 2,2'-(2,3,5,6-tetramethyl-2-yl)bis(4-chlorophenyl-5-diphenyl-imidazole) and ^{1}H NMR of it in CD_2Cl_2.

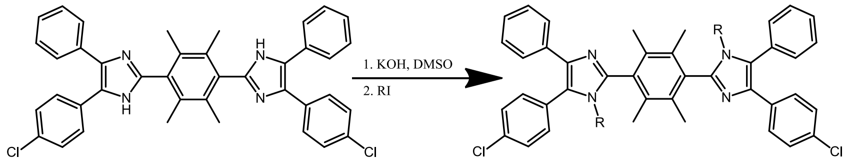

Supplementary Figure 50. Synthesis of monomers.

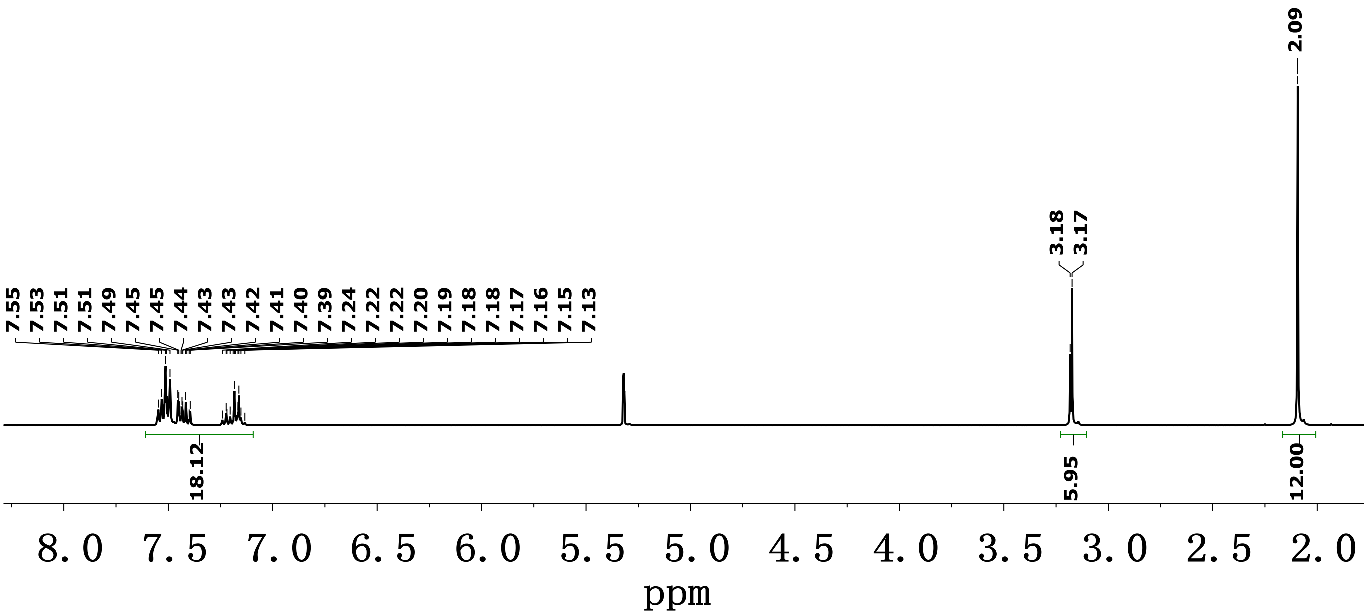

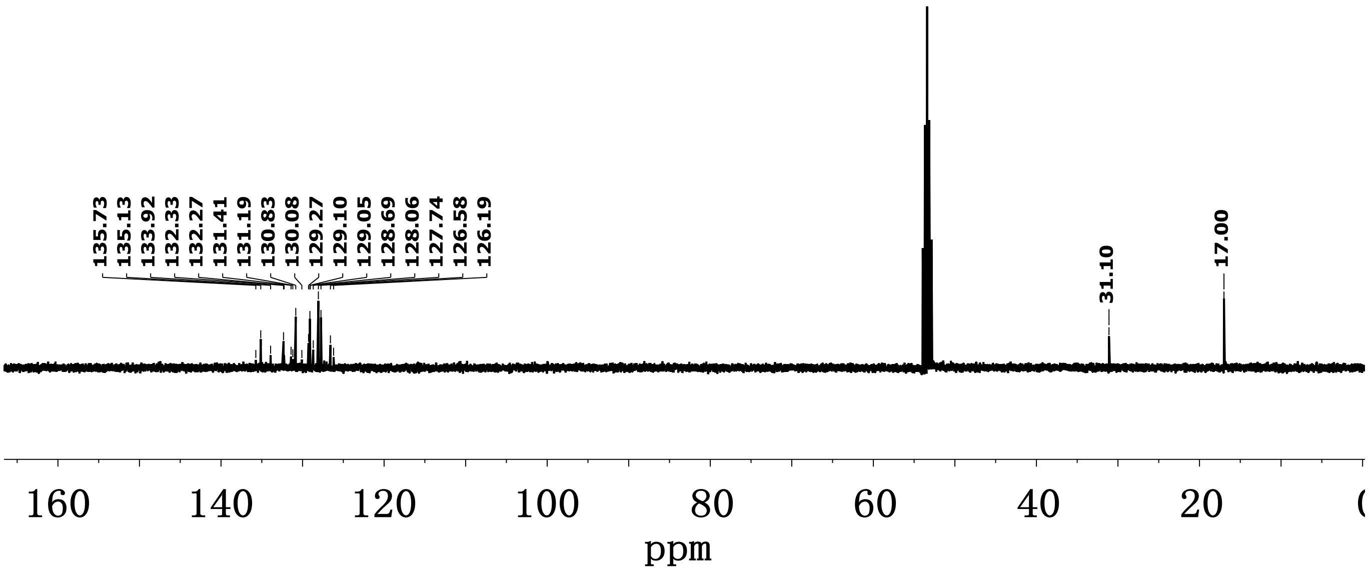

Supplementary Figure 51. ^{1}H and ^{13}C NMR of **18** in CD_2Cl_2.

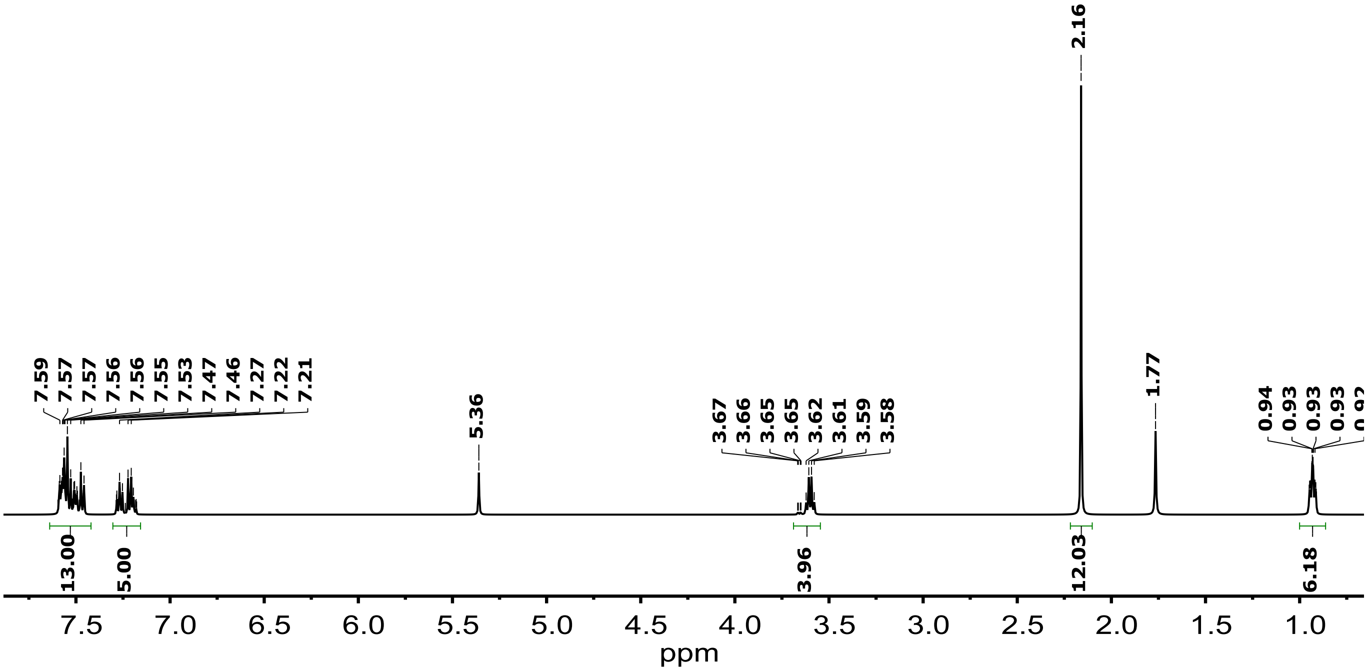

Supplementary Figure 52. ^{1}H NMR of **19** in CD_2Cl_2.

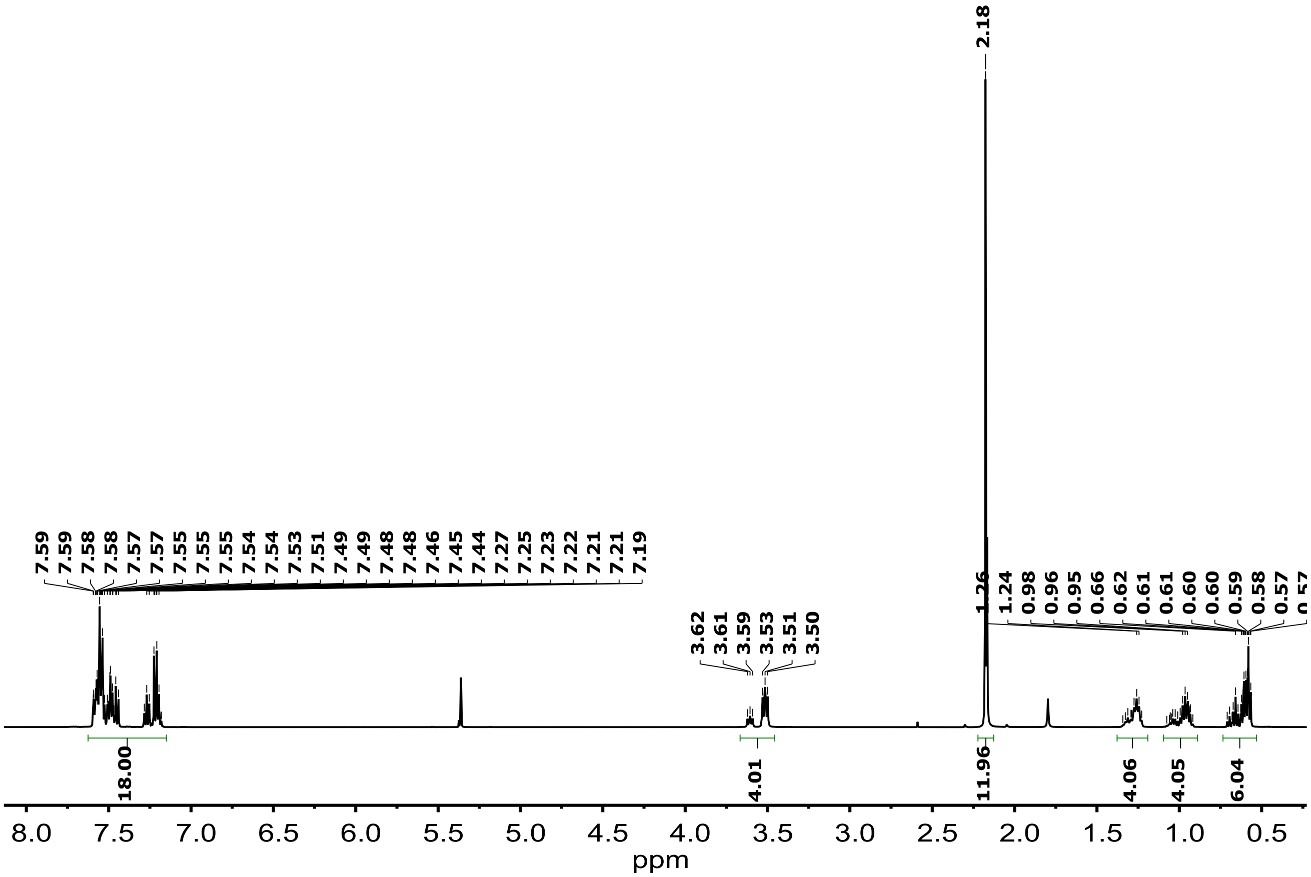

Supplementary Figure 53. ^{1}H NMR of **20** in CD_2Cl_2.

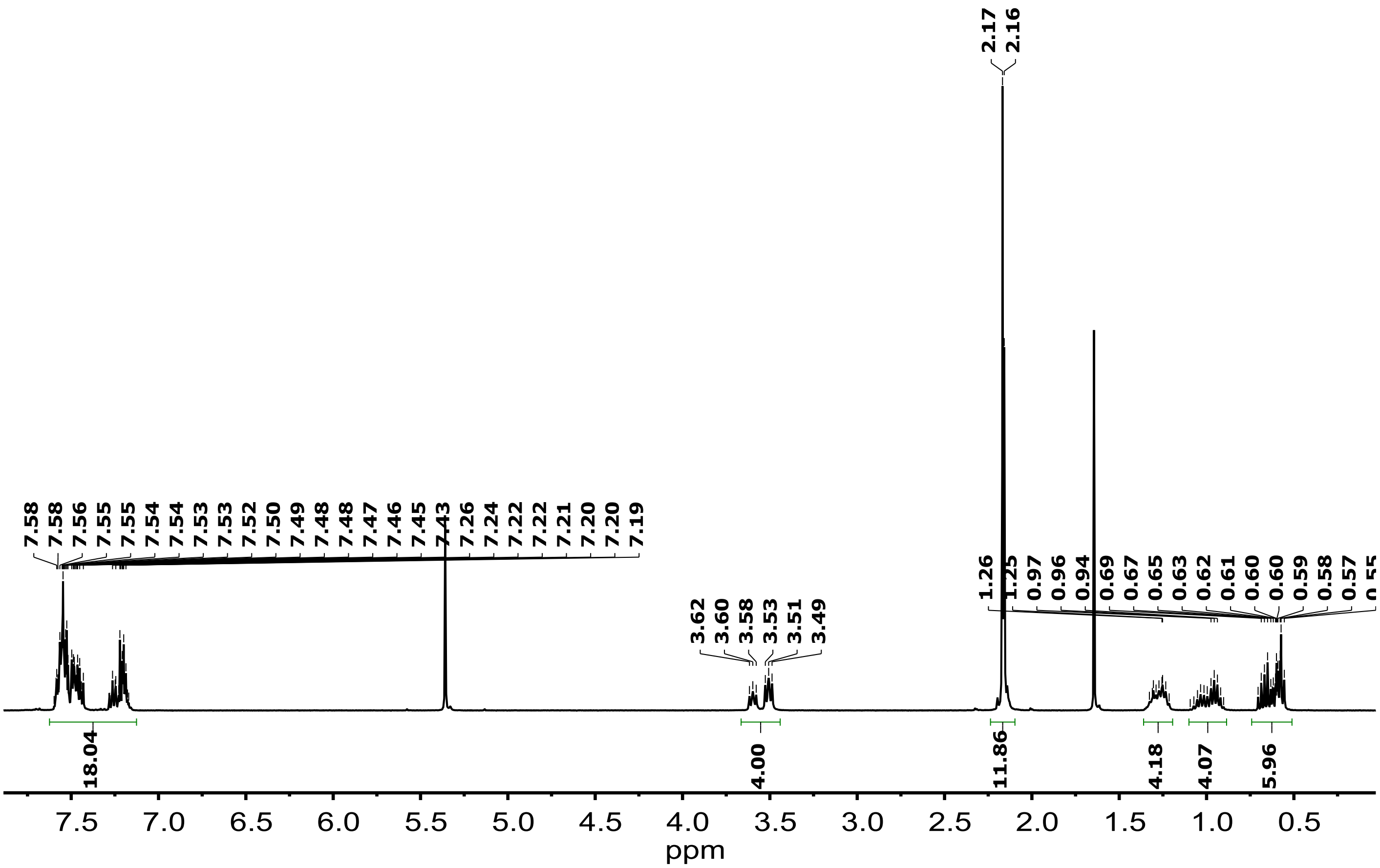

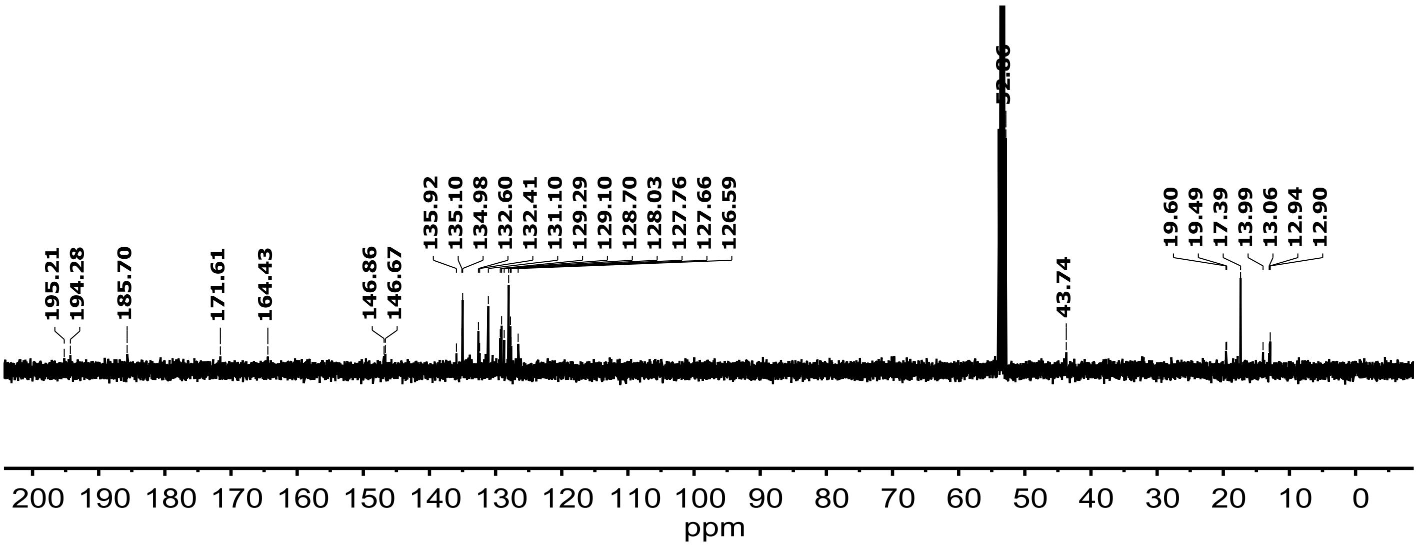

Supplementary Figure 54. ^{1}H and ^{13}C NMR of **21** in CD_2Cl_2.

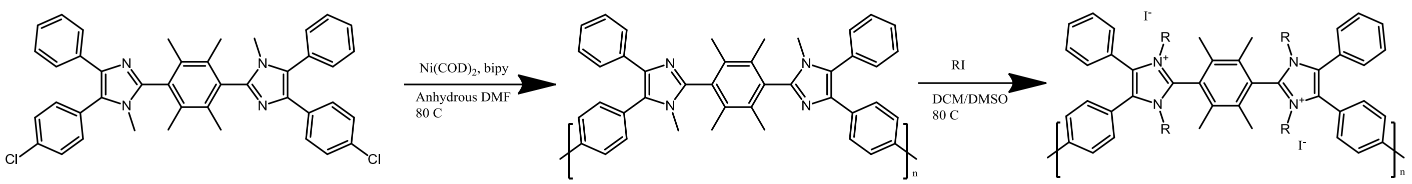

Supplementary Figure 55. Synthesis of PAImXY(#).

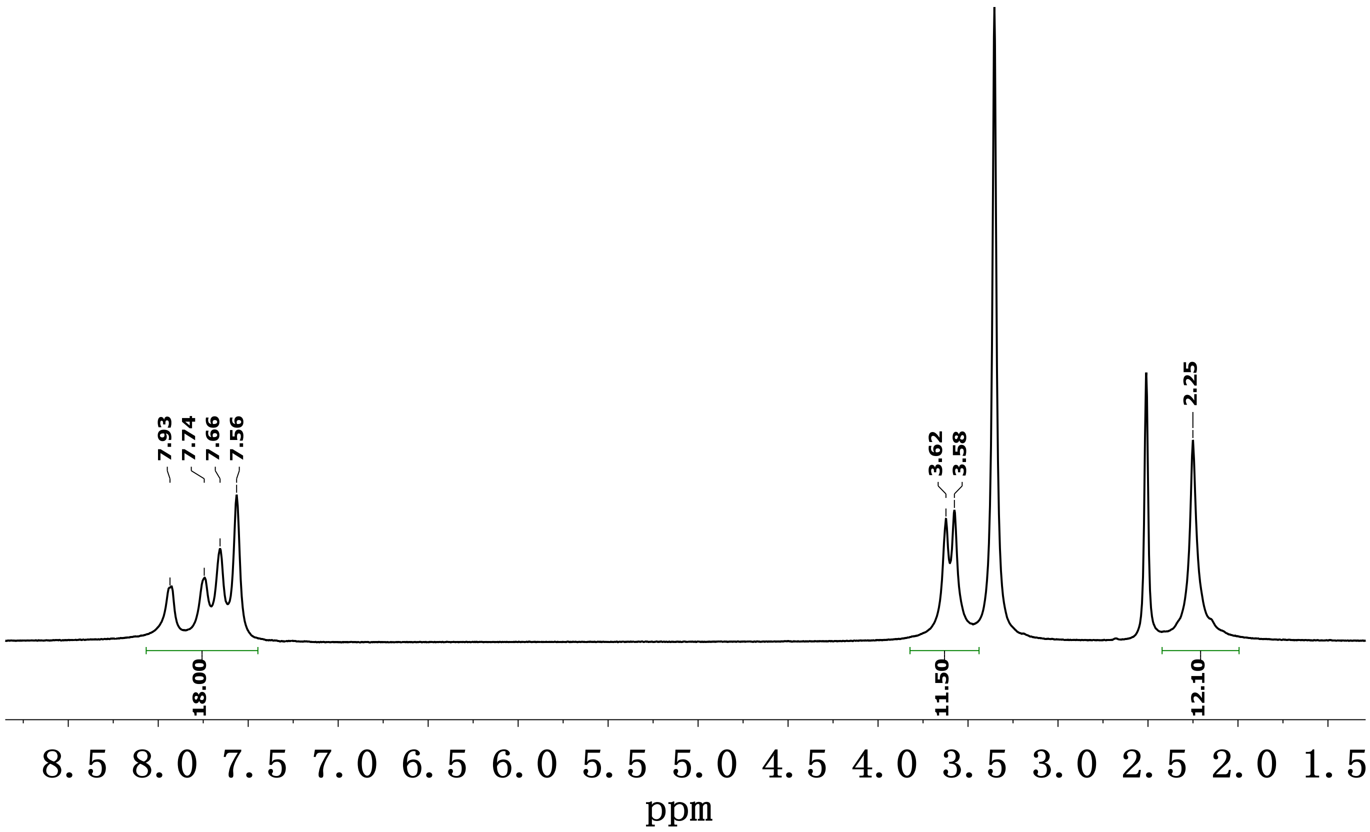

Supplementary Figure 56. ^{1}H NMR of PAImMM(**10**) in DMSO-*d*6.

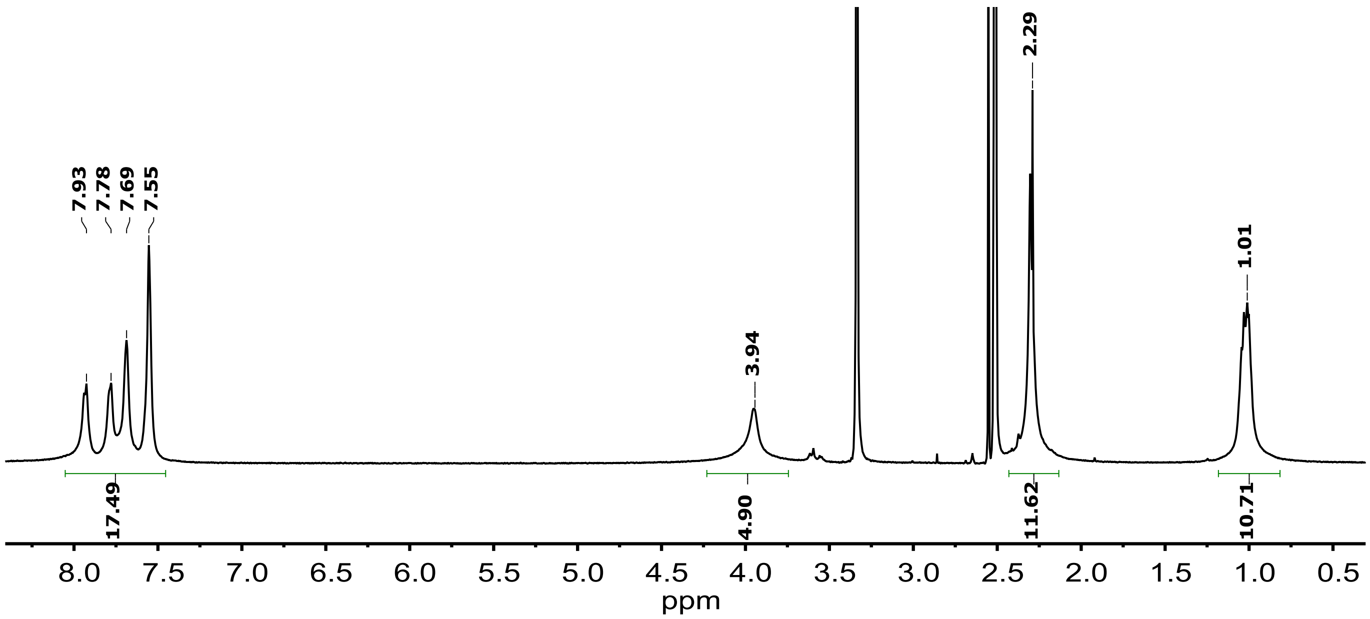

Supplementary Figure 57. ^{1}H NMR of PAImEE(**12**) in DMSO-*d*6.

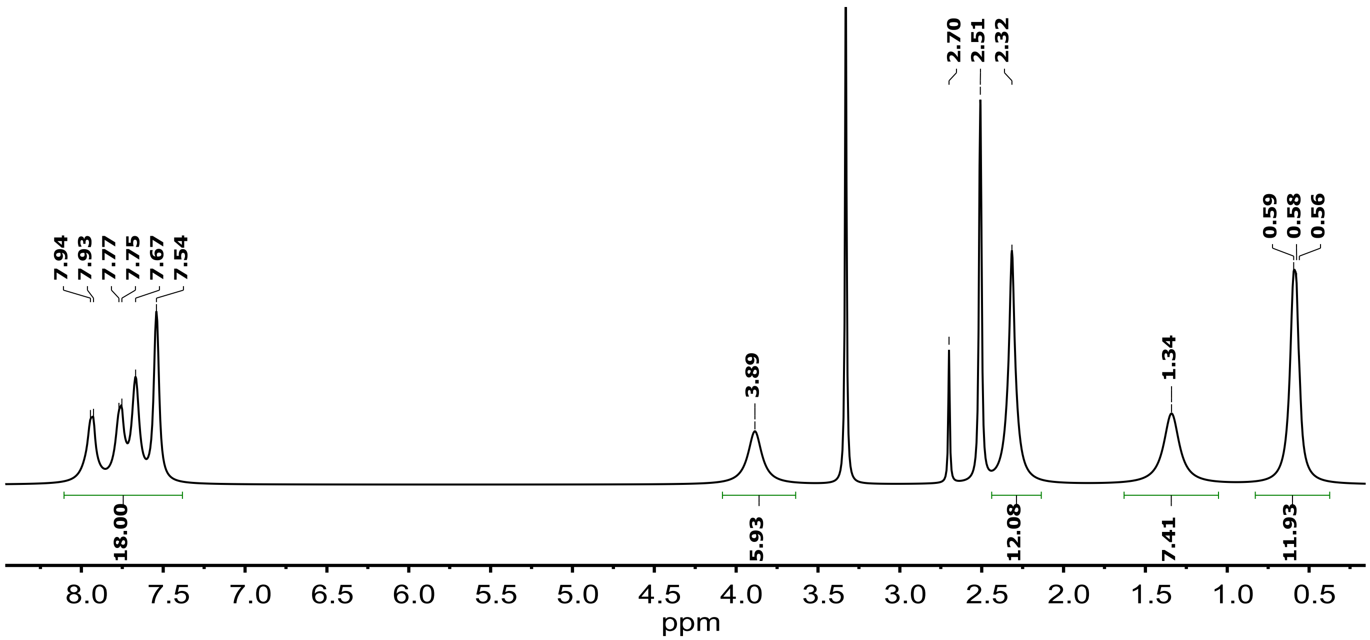

Supplementary Figure 58. ^{1}H NMR of PAImPP(**13**) in DMSO-*d*6.

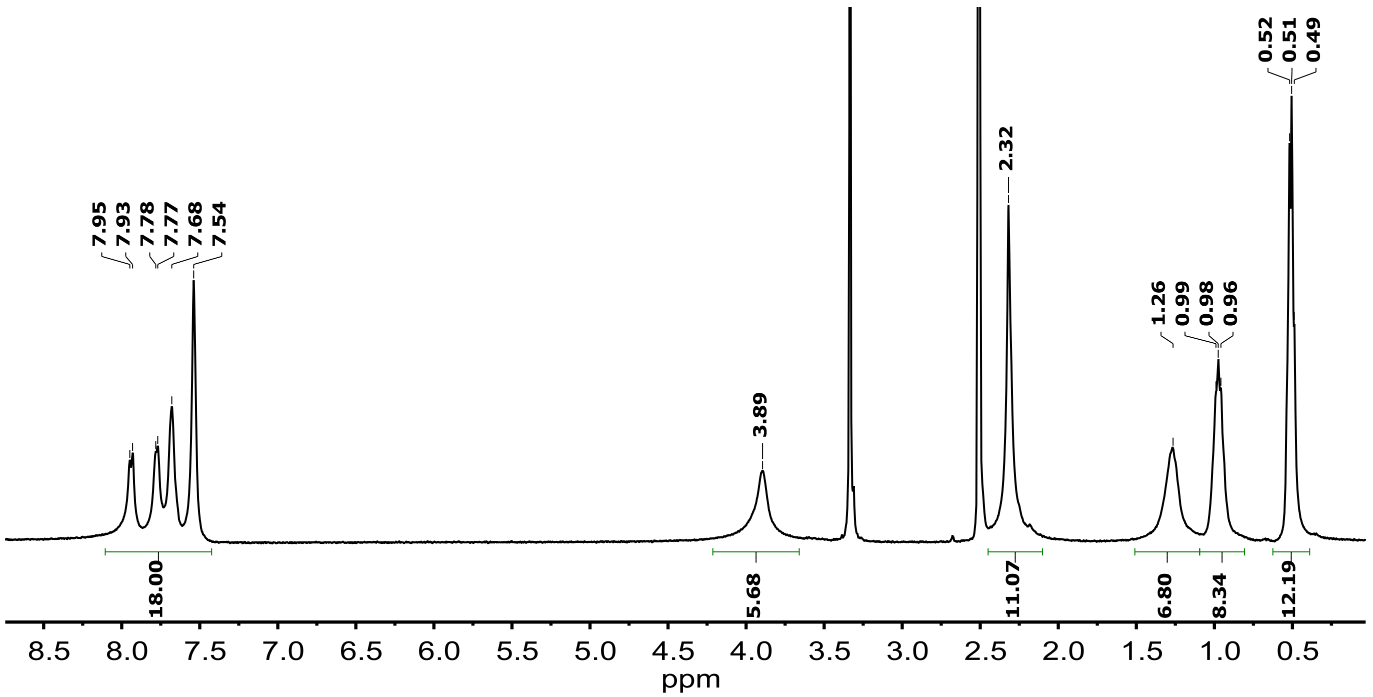

Supplementary Figure 59. ^{1}H NMR of PAImBB(**14**) in DMSO-*d*6.

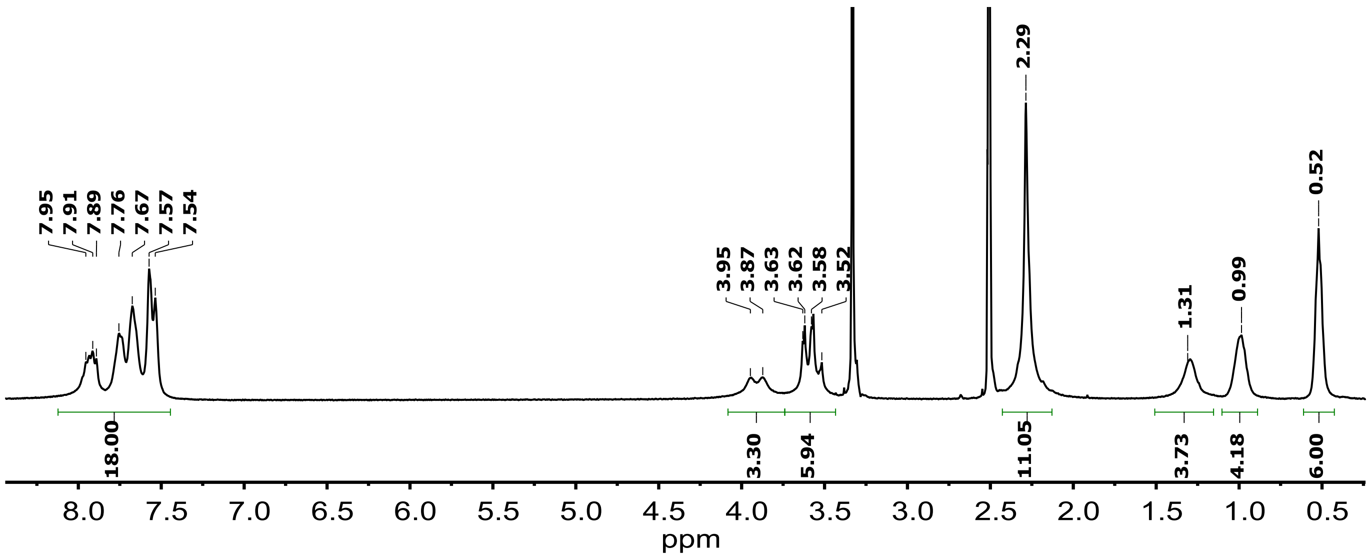

Supplementary Figure 60. ^{1}H NMR of PAImMB(**11**) in DMSO-*d*6.

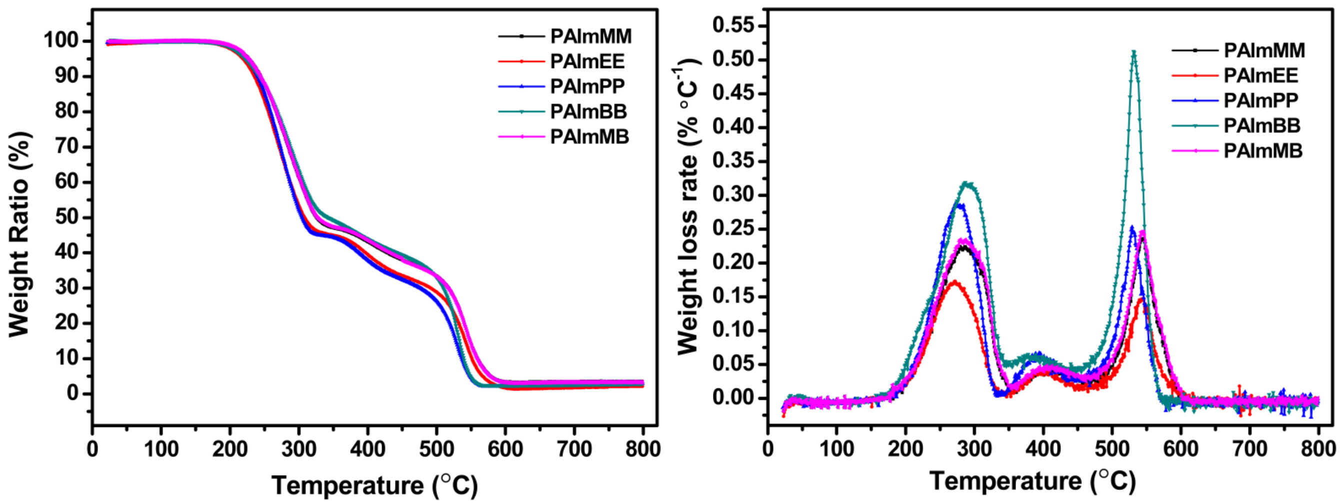

Supplementary Figure 61. **Thermogravimetric analysis (TGA) and DTG of PAImXY(n)**. All membranes were heated in N_2 atmosphere with 5 ˚C heating rate from room temperature to 800 ˚C.

Supplementary Tables

Supplementary Table 1. Fitted parameters of degradation rates and calculated half life time

Model compound	Degradation rate constant [h^{-1}]	Calculated half-life [h]
1	0.00375	>180
2	Too short to measure	
3	0.00115	>600
4	Too short to measure	
5	Too short to measure	
6	0.86348	0.8
7	Too short to measure	
8	0.03747	18.5
9	0.01729	40.1
10 (first slope)	0.02632	26.3
(second slope)		
	0.0116	59.8
11 (first slope)	0.0866	7.8
(second slope)	0.01725	40.2
12	0.01404	49.4
13 first slope)	0.04821	14.4
(second slope)	0.00578	119.9
14 first slope)	0.04723	14.7
(second slope)	0.00838	82.7

Supplementary Table 2. Crystallographic Information for compounds **10-12**

	**10**	**11**	**12**
molecular formula	C48H56N4O2I2	C50H57N4O3I2	C55H68N4OI2
formula weight (g mol^{-1})	974.79	1015.82	1054.96372
crystal dimensions (mm)	0.179, 0.293, 0.299	0.060, 0.419, 0.530	0.041, 0.155, 0.254
crystal system	monoclinic	monoclinic	monoclinic
space group	P21/n	P21/c	P21/c
a (Å)	10.5091(3)	18.3115(3)	18.9191(2)
b (Å)	15.9600(4)	14.8465(3)	16.2903(2)
c (Å)	14.3360(3)	18.2217(4)	17.6138(2)
α (deg)	90	90	90
β (deg)	104.9430(10)	91.114(2)	108.7780(10)
γ (deg)	90	90	90
V (Å3)	2323.2	4952.85(17)	5139.59(11)
Z	2	4	12
T (K)	296(2)	296(2)	150(2)
ρcalcd (g cm^{-3})	1.393	1.362	1.363
μ (mm)	10.938	10.300	9.914
2θmax (deg.)	68.27	65.83	66.88
total/unique reflections	4257/ 4257	8726/ 8726	9053/9053
obsd reflns [$I_0 \geq 2\sigma(I_0)$]	4014	6252	7620
R1, wR2 [$I_0 \geq 2\sigma(I_0)$][a]	0.0455, 0.1337	0.0690, 0.1939	0.0368, 0.0915
GOF	1.089	1.038	1.045
largest difference peak/hole (e$^-$/ Å^3)	0.962/ -0.735	1.590/ -1.780	2.768/ -0.748
CCDC no.	1852658	1852659	1852660

Supplementary Table 3. Crystallographic Information for compounds **13-14**

	**13**	**14**
molecular formula	$C_{58}H_{74}N_4OI_2$	$C_{50}H_{59}N_4O_{1.5}I_2$
formula weight (g mol^{-1})	1097.04	993.84
crystal dimensions (mm)	0.260, 0.400, 0.429	0.085, 0.145, 0.207
crystal system	triclinic	tetragonal
space group	P-1	I4/m
a (Å)	8.968(2)	16.3499(5)
b (Å)	10.8015(18)	16.3499(5)
c (Å)	16.278(5)	35.9904(12)
α (deg)	105.36(2)	90
β (deg)	98.73(2)	90
γ (deg)	94.876(18)	90
V (Å3)	1489.9(6)	9620.9(7)
Z	1	34
T (K)	150(2)	150(2)
ρcalcd (g cm^{-3})	1.216	1.396
μ (mm)	8.568	1.350
2θmax (deg.)	66.65	28.27
total/unique reflections	5130/5130	4490/4490
obsd reflns [$I_0 \geq 2\sigma(I_0)$]	5057	3967
R1, wR2 [$I_0 \geq 2\sigma(I_0)$][a]	0.0532, 0.1424	0.0392, 0.1055
GOF	1.093	1.060
largest difference peak/hole (e^-/ $Å^3$)	2.100/ -1.432	1.463/ -1.108
CCDC no.	1852661	1852662

Supplementary Table 4. Cartesian coordinates in Å for compound 8

Atom	X	Y	Z
C	1.04597	-0.680758	0.06646
C	1.044982	0.681891	-0.06658
N	-0.285856	-1.079074	0.106363
N	-0.287444	1.078302	-0.105898
C	-1.081426	-0.000947	0.000351
C	-2.556743	-0.001716	0.000573
C	-3.249347	-0.347704	-1.175371
C	-3.249088	0.338876	1.177562
C	-4.641267	-0.339935	-1.148666
C	-4.641566	0.323668	1.152862
C	-5.355606	-0.007141	0.002063
H	-5.18034	-0.601635	-2.050128
H	-5.180595	0.57283	2.057791
C	-0.756053	-2.451094	0.31483
H	-0.161339	-2.913351	1.096025
H	-1.795809	-2.418031	0.619173
H	-0.662705	-3.02605	-0.602055
C	-0.759883	2.449779	-0.312879
H	-0.164296	2.914643	-1.091837
H	-1.798885	2.41521	-0.619618
H	-0.669756	3.023137	0.60535
C	-2.524287	0.7165	2.445938
H	-1.701267	0.037105	2.667172
H	-2.10605	1.722833	2.379661
H	-3.206748	0.704064	3.292697
C	-2.523863	-0.728579	-2.442416
H	-1.706757	-0.043863	-2.669288
H	-2.097301	-1.730991	-2.370565
H	-3.207742	-0.726796	-3.288101
C	-6.861832	0.017154	-0.006319
H	-7.227771	0.988303	-0.347384
H	-7.266585	-0.737415	-0.679433
H	-7.266649	-0.153663	0.989933

C	2.178197	1.615499	-0.193952	
C	2.365236	2.652273	0.726755	
C	3.093922	1.457969	-1.239622	
C	3.446413	3.516544	0.599911	
H	1.677373	2.774092	1.55166	
C	4.176372	2.321905	-1.36082	
H	2.953061	0.663616	-1.958715	
C	4.353858	3.353379	-0.44322	
H	3.582893	4.311576	1.319481	
H	4.876652	2.191695	-2.173982	
H	5.194941	4.025751	-0.53939	
C	2.180451	-1.612924	0.193348	
C	2.369302	-2.648351	-0.728496	
C	3.095387	-1.455438	1.239701	
C	3.451493	-3.511414	-0.602066	
H	1.682063	-2.769976	-1.55396	
C	4.178854	-2.318164	1.36049	
H	2.953129	-0.662074	1.959615	
C	4.358135	-3.348339	0.441781	Charge = +1
H	3.589395	-4.305416	-1.322502	Multiplicity = 1
H	4.878534	-2.188024	2.174181	E0 = -1116.74340854 hartree

Supplementary Table 5. Cartesian coordinates in Å for compound 9

Atom	X	Y	Z
C	-1.338355	0.508115	0.218241
C	-1.207924	-0.809586	-0.124535
N	-0.050668	1.026133	0.323167
N	0.156775	-1.06881	-0.21549
C	0.842297	0.05583	0.057666
C	2.312113	0.199933	0.063174
C	3.004251	0.296533	-1.159492
C	3.001449	0.243958	1.29087
C	4.389474	0.438119	-1.126188
C	4.387036	0.379686	1.267066
C	5.099618	0.476925	0.07261
H	4.92557	0.524005	-2.062642
H	4.92227	0.411411	2.207429
C	0.289589	2.442753	0.560301
H	-0.333672	2.794954	1.377173
H	1.321892	2.467516	0.895503
C	0.766618	-2.394327	-0.439903
H	0.220438	-2.871248	-1.248751
H	1.780389	-2.217175	-0.782399
C	2.283362	0.170138	2.616413
H	1.851172	1.135779	2.887618
H	1.470639	-0.554771	2.608438
H	2.974742	-0.108599	3.408516
C	2.294747	0.236466	-2.490365
H	2.07712	-0.794742	-2.776975
H	1.347783	0.774096	-2.481899
H	2.917519	0.664653	-3.272712
C	6.601314	0.596805	0.077641
H	7.065007	-0.392306	0.083065
H	6.959924	1.120884	-0.806949
H	6.953804	1.126524	0.961515
C	-2.260237	-1.817667	-0.359612
C	-3.121366	-2.184342	0.679217

C	-2.430841	-2.393131	-1.623374
C	-4.132272	-3.11321	0.458484
H	-2.995022	-1.745263	1.658694
C	-3.43931	-3.325467	-1.839114
H	-1.788338	-2.099263	-2.441917
C	-4.291357	-3.687439	-0.799329
H	-4.791501	-3.389638	1.269421
H	-3.563879	-3.761358	-2.820458
H	-5.076499	-4.410886	-0.969549
C	-2.567715	1.294111	0.441127
C	-3.472498	1.489291	-0.606927
C	-2.858941	1.82456	1.702518
C	-4.645709	2.205585	-0.397272
H	-3.253748	1.084228	-1.584785
C	-4.030305	2.544842	1.90717
H	-2.180159	1.659344	2.527847
C	-4.92542	2.736909	0.858358
H	-5.337469	2.350976	-1.215174
H	-4.246469	2.947541	2.886807
H	-5.837002	3.295254	1.019833
C	0.10884	3.318132	-0.679253
H	-0.920899	3.244837	-1.032647
H	0.749288	2.945435	-1.48086
C	0.450897	4.781043	-0.383485
H	-0.188473	5.144507	0.424937
H	1.478467	4.845356	-0.016649
C	0.287996	5.68142	-1.60861
H	0.53723	6.715666	-1.370601
H	0.939975	5.359911	-2.422543
H	-0.738972	5.66194	-1.977194
C	0.75118	-3.270207	0.814663
H	1.331296	-2.783331	1.601075
H	-0.273746	-3.350482	1.177659
C	1.307108	-4.67737	0.558018
H	1.123607	-5.274805	1.452695

H	0.734671	-5.149505	-0.244701	
C	2.800676	-4.723398	0.224516	Charge = +1
H	3.392484	-4.255115	1.013105	Multiplicity = 1
H	3.139811	-5.754434	0.122426	E0 = -1352.70118274 hartree

Supplementary Table 6. Cartesian coordinates in Å for compound 10

Atom	X	Y	Z
C	4.98132	0.676905	0.10296
C	4.98132	-0.676906	-0.102938
N	3.649018	1.072376	0.160019
N	3.649018	-1.072377	-0.159999
C	2.857894	0	0.000009
C	1.377127	0	0.000006
C	0.701368	-0.005286	1.231013
C	0.701374	0.005285	-1.231004
C	-0.701371	0.004971	1.231011
C	-0.701366	-0.004972	-1.231009
C	3.175011	2.451398	0.31185
H	3.799886	3.105042	-0.287451
H	2.150233	2.508217	-0.036173
H	3.222884	2.753312	1.354238
C	3.175012	-2.451399	-0.31183
H	3.799885	-3.105042	0.287475
H	2.150232	-2.508217	0.03619
H	3.222888	-2.753314	-1.354217
C	1.442231	0.022789	-2.544196
H	1.08971	0.836229	-3.178309
H	2.513473	0.148172	-2.416678
H	1.279668	-0.904481	-3.09671
C	1.442219	-0.022788	2.544208
H	1.089692	-0.836225	3.178322
H	2.513461	-0.148177	2.416695
H	1.279659	0.904485	3.096717
C	6.115051	-1.611678	-0.216911
C	6.308752	-2.369473	-1.376967
C	7.023553	-1.734085	0.839413
C	7.3902	-3.237275	-1.474323
H	5.626395	-2.269211	-2.209544
C	8.106015	-2.600377	0.736542
H	6.877288	-1.155666	1.740499

C	8.290466	-3.354362	-0.418791
H	7.532768	-3.815294	-2.376568
H	8.801048	-2.688607	1.559763
H	9.131798	-4.028732	-0.497226
C	6.11505	1.611678	0.216935
C	6.308749	2.369472	1.376992
C	7.023554	1.734086	-0.839388
C	7.390197	3.237275	1.47435
H	5.626392	2.269208	2.209568
C	8.106015	2.600379	-0.736515
H	6.87729	1.155668	-1.740475
C	8.290464	3.354363	0.418819
H	7.532763	3.815292	2.376596
H	8.801049	2.688611	-1.559735
H	9.131795	4.028734	0.497255
C	-1.377126	-0.000001	-0.000001
C	-2.857894	0	-0.000004
N	-3.649018	1.072407	-0.159812
N	-3.64902	-1.072408	0.159798
C	-4.98132	0.676925	-0.102832
C	-3.175019	2.451461	-0.311373
C	-4.981321	-0.676924	0.10281
C	-3.175022	-2.451462	0.311363
C	-6.11505	1.611719	-0.216635
H	-3.799896	3.104983	0.28806
H	-2.15024	2.508218	0.036658
H	-3.222898	2.753579	-1.353703
C	-6.115052	-1.611718	0.216606
H	-3.799896	-3.104983	-0.288073
H	-2.150241	-2.50822	-0.036662
H	-3.222907	-2.753579	1.353692
C	-6.308744	2.369732	-1.376549
C	-7.023557	1.733927	0.839708
C	-6.308754	-2.36973	1.37652
C	-7.023553	-1.733927	-0.839742

C	-7.39019	3.237555	-1.473746	
H	-5.626381	2.269625	-2.20914	
C	-8.106018	2.60024	0.736995	
H	-6.877297	1.155339	1.740685	
C	-7.3902	-3.237552	1.473711	
H	-5.626397	-2.269621	2.209115	
C	-8.106014	-2.600241	-0.737036	
H	-6.877286	-1.15534	-1.740719	
C	-8.290461	3.354444	-0.418196	
H	-7.532753	3.815744	-2.375883	
H	-8.801055	2.688316	1.560229	
C	-8.290464	-3.354443	0.418156	
H	-7.532769	-3.81574	2.375848	
H	-8.801045	-2.688318	-1.560274	
H	-9.131791	4.028831	-0.496508	
H	-9.131795	-4.028829	0.496463	
C	-1.442213	-0.022137	-2.54421	
H	-1.089647	-0.835376	-3.178554	
H	-2.513449	-0.147616	-2.416734	
H	-1.279695	0.905301	-3.096455	
C	-1.442226	0.022135	2.544208	Charge = +2
H	-1.089659	0.835371	3.178557	Multiplicity = 1
H	-2.51346	0.147619	2.416727	E0 = -1922.49462295 hartree

Supplementary Table 7. Cartesian coordinates in Å for compound 11

Atom	X	Y	Z
C	-4.968689	0.339738	-0.214946
C	-4.875868	-0.999627	0.051183
N	-3.666602	0.828952	-0.275947
N	-3.520171	-1.296465	0.141445
C	-2.804988	-0.178318	-0.0574
C	-1.326396	-0.084919	-0.055314
C	-0.648303	-0.090876	-1.285676
C	-0.653494	-0.000291	1.174326
C	0.752076	-0.002358	-1.285443
C	0.746867	0.085772	1.174263
C	-1.392765	0.002031	2.488473
H	-1.149676	0.890691	3.071787
H	-2.471049	-0.022465	2.362443
H	-1.110674	-0.860541	3.09441
C	-1.381217	-0.197846	-2.599189
H	-1.022992	-1.052745	-3.173522
H	-2.452987	-0.316225	-2.472496
H	-1.214779	0.688536	-3.213558
C	-5.942717	-2.007338	0.189054
C	-6.103551	-2.725637	1.378854
C	-6.821133	-2.240124	-0.87408
C	-7.123647	-3.662103	1.499457
H	-5.444372	-2.541714	2.215763
C	-7.842589	-3.17485	-0.748045
H	-6.699237	-1.693638	-1.798376
C	-7.994838	-3.888271	0.437308
H	-7.241451	-4.208623	2.424608
H	-8.514926	-3.347973	-1.576616
H	-8.78862	-4.615849	0.533771
C	-6.174521	1.169265	-0.405688
C	-6.444267	1.769822	-1.640043
C	-7.079143	1.335128	0.647519
C	-7.595098	2.530433	-1.81265

H	-5.76586	1.627873	-2.469986
C	-8.231683	2.0922	0.469516
H	-6.876379	0.874602	1.604078
C	-8.49019	2.693321	-0.758905
H	-7.795495	2.987503	-2.771524
H	-8.923948	2.21453	1.290718
H	-9.3858	3.283186	-0.895719
C	1.424904	0.07815	-0.055477
C	2.904333	0.167495	-0.051505
N	3.625219	1.292643	-0.177955
N	3.761649	-0.856248	0.09301
C	4.979761	0.983809	-0.111056
C	5.065864	-0.370083	0.058229
C	3.381466	-2.267146	0.307844
C	6.053305	1.98964	-0.210553
C	6.261969	-1.218145	0.252025
H	4.163298	-2.704261	0.923834
H	2.465326	-2.263682	0.890125
C	6.172497	3.006912	0.742661
C	6.980426	1.919976	-1.255025
C	6.811	-1.942904	-0.809557
C	6.866048	-1.283308	1.511372
C	7.199224	3.938999	0.648366
H	5.474523	3.060937	1.566494
C	8.008725	2.851884	-1.343615
H	6.891216	1.141574	-1.999124
C	7.94355	-2.725358	-0.611784
H	6.360976	-1.885044	-1.790611
C	7.99925	-2.065808	1.705469
H	6.447694	-0.721665	2.334883
C	8.118925	3.863358	-0.394195
H	7.284412	4.718434	1.392468
H	8.719181	2.789196	-2.155924
C	8.538074	-2.788764	0.645208
H	8.362722	-3.279676	-1.439746

H	8.459377	-2.109382	2.68267
H	8.917899	4.588203	-0.464911
H	9.4193	-3.39646	0.796841
C	1.481213	0.192165	2.486968
H	1.281529	-0.674439	3.118884
H	2.556967	0.268136	2.359741
H	1.154486	1.071624	3.043265
C	1.491802	0.016593	-2.599439
H	1.19759	0.881642	-3.196105
H	2.569516	0.055655	-2.471864
H	1.262008	-0.868525	-3.19331
C	-3.283485	2.244094	-0.452897
H	-3.911303	2.653745	-1.238533
H	-2.258556	2.252146	-0.812284
C	-3.414633	3.061068	0.831832
H	-4.442254	3.006095	1.194948
H	-2.779709	2.624703	1.605014
C	3.21365	-3.055709	-0.991897
H	2.399829	-2.624544	-1.577004
H	4.121079	-2.954085	-1.587062
C	-3.02388	4.52413	0.605634
H	-1.998576	4.570985	0.229964
H	-3.658272	4.950462	-0.175474
C	2.937213	-4.544979	-0.744085
H	2.974953	-5.053467	-1.708962
H	3.749209	-4.967353	-0.146462
C	-3.141894	5.366295	1.876516
H	-4.165019	5.36415	2.256046
H	-2.49342	4.981434	2.66538
H	-2.858554	6.401874	1.687674
C	1.592977	-4.843842	-0.074629
H	1.534595	-4.435168	0.934763
H	0.766235	-4.426289	-0.652174
H	1.433911	-5.919535	0.002696
C	-2.952776	-2.631175	0.356429

H	-2.982319	-2.886986	1.411912	
H	-3.530476	-3.353208	-0.210703	
H	-1.925893	-2.634609	0.0101	
C	3.069814	2.634661	-0.377047	Charge = +2
H	3.146208	3.210256	0.540802	Multiplicity = 1
H	3.621157	3.13128	-1.168802	E0 = -2158.45148025 hartree

Supplementary Table 8. Cartesian coordinates in Å for compound 12

Atom	X	Y	Z
C	-4.984455	0.682736	-0.040406
C	-4.984441	-0.682738	0.040473
N	-3.651782	1.084704	-0.062041
N	-3.65176	-1.084679	0.062111
C	-2.861831	0.000021	0.000033
C	-1.379233	0.000024	0.000018
C	-0.701576	0.136446	-1.222816
C	-0.701542	-0.13641	1.222832
C	0.701542	0.136427	-1.222837
C	0.701576	-0.136429	1.22281
C	-1.438198	-0.282033	2.530581
H	-1.141481	0.49667	3.23387
H	-2.515952	-0.225293	2.413082
H	-1.207432	-1.238176	3.003177
C	-1.438268	0.282123	-2.530538
H	-1.141458	-0.496447	-3.233931
H	-2.516013	0.225227	-2.413032
H	-1.207637	1.23836	-3.003015
C	-6.130204	-1.612647	0.085189
C	-6.381306	-2.386716	1.223036
C	-6.996294	-1.704201	-1.008641
C	-7.475015	-3.244175	1.259862
H	-5.734155	-2.306144	2.085456
C	-8.092132	-2.559049	-0.966101
H	-6.807575	-1.110272	-1.891676
C	-8.331754	-3.332187	0.1661
H	-7.661585	-3.835592	2.145243
H	-8.754779	-2.622576	-1.817805
H	-9.18314	-3.997717	0.197494
C	-6.13024	1.612617	-0.08514
C	-6.381321	2.386705	-1.222979
C	-6.996374	1.704121	1.00866
C	-7.475054	3.244135	-1.259824

H	-5.73414	2.30617	-2.085378
C	-8.092234	2.558939	0.966099
H	-6.80767	1.110178	1.891689
C	-8.331835	3.332098	-0.166093
H	-7.661607	3.835567	-2.145199
H	-8.754914	2.622427	1.81778
H	-9.183238	3.997604	-0.197503
C	1.379233	-0.000009	-0.000024
C	2.861831	-0.00001	-0.000039
N	3.651766	1.084685	-0.062109
N	3.651776	-1.084698	0.062038
C	4.984445	0.682737	-0.040464
C	3.172087	2.482962	-0.052775
C	4.984452	-0.682737	0.040408
C	3.172109	-2.482979	0.052701
C	6.130214	1.61264	-0.085177
H	3.761627	3.029585	-0.781761
H	2.145907	2.46538	-0.405491
C	6.130231	-1.612625	0.08514
H	3.761597	-3.029582	0.781746
H	2.145902	-2.465393	0.40534
C	6.99631	1.704181	1.008649
C	6.381315	2.386716	-1.223021
C	6.99636	-1.704137	-1.008663
C	6.38131	-2.386714	1.222979
C	8.092152	2.559023	0.96611
H	6.807592	1.110248	1.891681
C	7.47503	3.244169	-1.259845
H	5.734161	2.306153	-2.085438
C	8.092214	-2.558963	-0.966104
H	6.807659	-1.110194	-1.891691
C	7.475036	-3.244151	1.259822
H	5.734131	-2.306173	2.08538
C	8.331773	3.332168	-0.166087
H	8.754804	2.62254	1.817811

H	7.661599	3.835591	-2.145223	
C	8.331814	-3.332121	0.166088	
H	8.754892	-2.622457	-1.817787	
H	7.661589	-3.835584	2.145198	
H	9.183163	3.997693	-0.19748	
H	9.183213	-3.997634	0.197496	
C	1.438268	-0.282103	2.530533	
H	1.207627	-1.238333	3.003019	
H	2.516013	-0.225219	2.413026	
H	1.141466	0.496477	3.233919	
C	1.438198	0.282049	-2.530586	
H	1.207435	1.238193	-3.003182	
H	2.515952	0.225306	-2.413088	
H	1.14148	-0.496653	-3.233876	
C	-3.172076	-2.482954	0.052774	
H	-3.761628	-3.029583	0.781746	
H	-2.145902	-2.465372	0.405508	
C	-3.172122	2.482988	-0.052708	
H	-3.761604	3.029585	-0.781761	
H	-2.145911	2.465404	-0.405336	
C	-3.264038	3.129744	1.324082	
H	-4.290727	3.158212	1.6833	
H	-2.653177	2.600128	2.052707	
H	-2.89963	4.153747	1.253603	
C	-3.263847	-3.129659	-1.324049	
H	-4.290499	-3.158105	-1.683378	
H	-2.652904	-2.600019	-2.052588	
H	-2.899454	-4.153667	-1.25357	
C	3.264007	-3.12973	-1.324092	
H	2.899608	-4.153736	-1.25361	
H	2.653129	-2.600116	-2.052704	
H	4.29069	-3.158188	-1.68333	
C	3.26388	3.129678	1.324042	Charge = +2
H	2.899493	4.153688	1.253558	Multiplicity = 1
H	2.652942	2.600049	2.052593	E0 = -2079.80389233 hartree

Supplementary Table 9. Cartesian coordinates in Å for compound 13

Atom	X	Y	Z
C	-4.984654	0.674917	-0.110442
C	-4.984654	-0.674918	0.110444
N	-3.651896	1.072809	-0.172987
N	-3.651896	-1.07281	0.172988
C	-2.861969	0	0
C	-1.379355	0	0
C	-0.701579	0.015656	-1.230309
C	-0.701579	-0.015657	1.230309
C	0.701578	0.015658	-1.230309
C	0.701579	-0.015659	1.230308
C	-1.438312	-0.032151	2.546094
H	-1.137508	0.808791	3.171637
H	-2.515949	0.018333	2.423988
H	-1.211225	-0.939675	3.108036
C	-1.438313	0.032151	-2.546094
H	-1.137509	-0.808791	-3.171638
H	-2.51595	-0.018333	-2.423988
H	-1.211226	0.939675	-3.108036
C	-6.13049	-1.594761	0.252244
C	-6.377107	-2.251279	1.462741
C	-7.001548	-1.793894	-0.823208
C	-7.470944	-3.099954	1.589363
H	-5.726271	-2.085746	2.31023
C	-8.097586	-2.639216	-0.691016
H	-6.816667	-1.290607	-1.761629
C	-8.332434	-3.295802	0.513478
H	-7.653857	-3.600189	2.530006
H	-8.764089	-2.786592	-1.529241
H	-9.183857	-3.954303	0.614636
C	-6.13049	1.594761	-0.252242
C	-6.377107	2.251278	-1.462739
C	-7.001547	1.793895	0.82321
C	-7.470945	3.099954	-1.589361

H	-5.726272	2.085745	-2.310229
C	-8.097584	2.639217	0.691018
H	-6.816665	1.290608	1.761631
C	-8.332433	3.295803	-0.513476
H	-7.653858	3.600188	-2.530004
H	-8.764087	2.786594	1.529244
H	-9.183857	3.954303	-0.614633
C	1.379355	0	-0.000001
C	2.861969	0	-0.000001
N	3.651896	1.072813	-0.172965
N	3.651896	-1.072813	0.172965
C	4.984654	0.67492	-0.11043
C	3.173483	2.463629	-0.303574
C	4.984654	-0.67492	0.110429
C	3.173483	-2.463629	0.303573
C	6.13049	1.594767	-0.252205
H	3.777539	2.943405	-1.067983
H	2.153	2.415553	-0.672302
C	6.13049	-1.594767	0.252205
H	3.77754	-2.943407	1.067979
H	2.153001	-2.415554	0.672302
C	7.001548	1.793865	0.823254
C	6.377109	2.25132	-1.462681
C	7.001549	-1.793865	-0.823254
C	6.377109	-2.25132	1.462681
C	8.097587	2.639189	0.691089
H	6.816664	1.29055	1.76166
C	7.470949	3.099998	-1.589276
H	5.726275	2.085815	-2.310177
C	8.097588	-2.639189	-0.691088
H	6.816665	-1.290551	-1.761659
C	7.470948	-3.099998	1.589277
H	5.726274	-2.085815	2.310177
C	8.332437	3.295811	-0.513385
H	8.76409	2.786539	1.529319

H	7.653863	3.600261	-2.529903
C	8.332438	-3.295811	0.513386
H	8.764091	-2.786538	-1.529317
H	7.653862	-3.600261	2.529904
H	9.183862	3.954314	-0.614522
H	9.183863	-3.954313	0.614524
C	1.438314	-0.032163	2.546092
H	1.211214	-0.939683	3.108035
H	2.515952	0.018305	2.423984
H	1.137524	0.808784	3.171636
C	1.438313	0.032163	-2.546093
H	1.211212	0.939683	-3.108036
H	2.515951	-0.018304	-2.423986
H	1.137523	-0.808785	-3.171637
C	-3.173486	-2.463624	0.303619
H	-3.77755	-2.94339	1.068027
H	-2.153006	-2.415546	0.672355
C	-3.173486	2.463624	-0.303619
H	-3.77755	2.943389	-1.068028
H	-2.153006	2.415545	-0.672355
C	-3.237776	3.243463	1.009392
H	-4.263849	3.249085	1.377781
H	-2.632035	2.735387	1.760422
C	-3.237776	-3.243463	-1.009393
H	-4.26385	-3.249085	-1.377781
H	-2.632035	-2.735386	-1.760422
C	3.237781	-3.24345	-1.009449
H	2.632058	-2.735355	-1.760479
H	4.26386	-3.249078	-1.377823
C	3.237783	3.243451	1.009446
H	2.63206	2.735356	1.760478
H	4.263862	3.24908	1.377819
C	-2.743149	4.677871	0.820198
H	-1.708968	4.69846	0.473938
H	-3.352487	5.213362	0.090973

H	-2.791163	5.224531	1.761003	
C	-2.743149	-4.67787	-0.820199	
H	-1.708968	-4.69846	-0.47394	
H	-3.352487	-5.213363	-0.090975	
H	-2.791163	-5.22453	-1.761005	
C	2.743134	-4.677854	-0.820283	
H	2.79116	-5.224502	-1.761094	
H	3.35245	-5.213363	-0.091053	
H	1.708945	-4.698433	-0.474044	
C	2.743134	4.677854	0.82028	Charge = +2
H	2.791161	5.224504	1.76109	Multiplicity = 1
H	3.352449	5.213363	0.091048	E0 = -2237.10897140 hartree

Supplementary Table 10. Cartesian coordinates in Å for compound 14

Atom	X	Y	Z
C	-4.930243	0.664748	-0.160034
C	-4.930243	-0.664746	0.159998
N	-3.597609	1.0573	-0.250661
N	-3.597609	-1.057298	0.250626
C	-2.806944	0.000001	-0.000016
C	-1.323583	0.000001	-0.000011
C	-0.645024	-0.087772	-1.226976
C	-0.645036	0.087776	1.226961
C	0.758454	-0.092096	-1.22676
C	0.758442	0.092101	1.226758
C	-1.381303	0.177026	2.54013
H	-1.076994	1.062313	3.099005
H	-2.458695	0.222402	2.414557
H	-1.157917	-0.685693	3.170158
C	-1.38128	-0.177021	-2.540152
H	-1.076967	-1.062309	-3.099024
H	-2.458672	-0.222395	-2.414589
H	-1.157887	0.685698	-3.170178
C	-6.07673	-1.569042	0.376678
C	-6.318637	-2.12681	1.636618
C	-6.953873	-1.84995	-0.675222
C	-7.4143	-2.958988	1.835985
H	-5.662333	-1.896322	2.464515
C	-8.051594	-2.678561	-0.470655
H	-6.772817	-1.423216	-1.651552
C	-8.282045	-3.236606	0.783366
H	-7.593693	-3.382263	2.81433
H	-8.723029	-2.889782	-1.291094
H	-9.134934	-3.882032	0.940798
C	-6.07673	1.569044	-0.376716
C	-6.318635	2.126813	-1.636657
C	-6.953874	1.849952	0.675183
C	-7.414298	2.958991	-1.836025

H	-5.66233	1.896325	-2.464553
C	-8.051595	2.678563	0.470615
H	-6.772819	1.423218	1.651513
C	-8.282044	3.236609	-0.783407
H	-7.593689	3.382267	-2.814369
H	-8.723031	2.889785	1.291053
H	-9.134932	3.882035	-0.940839
C	1.437169	0.000001	0.000002
C	2.920238	0.000001	0.00001
N	3.711049	1.049604	-0.28151
N	3.711044	-1.049603	0.281538
C	5.043668	0.659698	-0.179276
C	3.240507	2.423242	-0.551629
C	5.043664	-0.6597	0.179322
C	3.240498	-2.42324	0.551652
C	6.190868	1.556604	-0.422317
H	3.848832	2.818364	-1.360268
H	2.221462	2.341107	-0.914809
C	6.190859	-1.55661	0.42237
H	3.848832	-2.81837	1.36028
H	2.221457	-2.341104	0.914846
C	7.061719	1.876454	0.623636
C	6.439932	2.067657	-1.700514
C	7.061709	-1.87647	-0.62358
C	6.439917	-2.067659	1.70057
C	8.160595	2.697286	0.39509
H	6.875123	1.485716	1.613904
C	7.536784	2.892135	-1.924049
H	5.788209	1.806805	-2.523031
C	8.16058	-2.697308	-0.395028
H	6.875118	-1.485736	-1.61385
C	7.536764	-2.892143	1.924111
H	5.788194	-1.8068	2.523084
C	8.398362	3.208622	-0.877334
H	8.827253	2.938683	1.211085

H	7.72187	3.279181	-2.916236
C	8.398342	-3.20864	0.877398
H	8.827237	-2.938714	-1.211021
H	7.721845	-3.279185	2.9163
H	9.252166	3.847962	-1.053527
H	9.252141	-3.847984	1.053596
C	1.493872	0.198804	2.539162
H	1.262437	-0.6487	3.186319
H	2.571748	0.233487	2.414794
H	1.195907	1.098101	3.07919
C	1.493895	-0.198795	-2.539158
H	1.262476	0.648717	-3.186309
H	2.571771	-0.233492	-2.414779
H	1.195927	-1.098085	-3.079197
C	-3.125009	-2.438408	0.474397
H	-3.722146	-2.857297	1.279042
H	-2.099281	-2.371131	0.825131
C	-3.125006	2.43841	-0.474434
H	-3.722142	2.857298	-1.279079
H	-2.099278	2.37113	-0.825166
C	-3.216576	3.307067	0.779819
H	-4.242084	3.300562	1.152406
H	-2.590119	2.878137	1.564088
C	-3.216579	-3.307064	-0.779856
H	-4.242086	-3.300553	-1.152447
H	-2.590117	-2.878136	-1.564123
C	3.326977	-3.326419	-0.680596
H	2.660937	-2.94432	-1.45672
H	4.34026	-3.280634	-1.080231
C	3.327004	3.326429	0.680612
H	2.66098	2.944331	1.456751
H	4.340295	3.280651	1.080229
C	-2.782358	4.747692	0.496758
H	-1.762345	4.749352	0.105262
H	-3.41528	5.166309	-0.289686

C	2.977779	-4.787146	-0.366563	
H	3.198195	-5.379431	-1.256292	
H	3.645749	-5.155364	0.416423	
C	2.977794	4.787153	0.366577	
H	3.198225	5.379443	1.256298	
H	3.645747	5.155368	-0.416424	
C	-2.855037	5.636675	1.738796	
H	-3.871797	5.67931	2.132808	
H	-2.206535	5.25966	2.531393	
H	-2.543321	6.655828	1.509674	
C	1.521914	5.022869	-0.042564	
H	1.26927	4.518937	-0.976185	
H	0.836844	4.663208	0.727061	
H	1.330268	6.086267	-0.186749	
C	1.521908	-5.022872	0.042605	
H	1.269279	-4.518946	0.976232	
H	0.836822	-4.663211	-0.727006	
H	1.330271	-6.086272	0.186787	
C	-2.78237	-4.747692	-0.496795	
H	-1.76236	-4.749357	-0.105294	
H	-3.415298	-5.166306	0.289645	
C	-2.855047	-5.636673	-1.738836	Charge = +2
H	-3.871806	-5.6793	-2.132854	Multiplicity = 1
H	-2.206538	-5.25966	-2.531427	E0 = -2394.40986690 hartree

Supplementary Methods

Materials and chemicals

Chemicals were purchased from Sigma Aldrich unless otherwise noted. Acetic acid (glacial) and potassium iodide (99.0%) were purchased from Caledon Laboratories Ltd. 1,4-Phenylenediboronic acid (97%) were purchased from Combi-Blocks, Inc.. Ethanol (anhydrous grade) was purchased from Commercial Alcohols Inc.. Potassium hydroxide (ACS grade, pellets) was purchased from Macron Fine Chemicals™. Dimethylsulfoxide (spectrograde), potassium carbonate (99.0%), potassium chloride (ACS grade), sodium bicarbonate (ACS grade), and hexanes (ACS grade) were purchased from ACP Chemicals Inc.. Methylene chloride (ACS grade, stabilized), sodium dithionite, acetone (ACS grade), and methanol (ACS grade), were purchased from Fisher Scientific. Chloroform (ACS grade) and sodium hydroxide (ACS grade) were purchased from BDH Chemicals. Tetrakis(triphenylphosphine)palladium (99%) was purchased from Strem Chemicals. Dimethylsulfoxide-d6 (99.9%-D), dichloromethane-d2 (99.8%-D), chloroform-D (99.8%-D), methanol-d4 (99.8%-D) were purchased from Cambridge Isotope Laboratories, Inc.. Nuclear magnetic resonance (NMR) spectra were obtained on a 400 or 500 MHz Bruker AVANCE III running IconNMR under Top Spin 2.1. The residual ^{1}H NMR solvent peaks for DMSO-d6, CD_2Cl_2, $CDCl_3$, and MeOD were set to 2.50 ppm, 5.32 ppm, 7.26 ppm, and 3.31 ppm, respectively.

Synthesis of compound 9

Compound **9** was synthesised following our previously reported route for compound **8**.[1] 2.030g precursor of compound **8** (6.0 mmol) was dissloved in 20 mL DMSO. 1.3 mL of 5M KOH_{aq} was added. The solution colour changed from light yellow to dark red. After 10 min, bromobutane (0.70 mL, 6.5 mmol) was added and reacted for 18 hours and poured into DI water containing 2.0g KOH. Diethyleather was added to extract the monobutylated product. The organic layer was decanted and washed with water, brine, and water, dried over anhydrous $MgSO_4$, filtered, and dried under reduced pressure. The product was redissloved in 20 mL acetonitrile and 6.5 mL of bromobutane (60.0 mmol) was added and reacted at 80 ˚C for three days. Acetonitrile was removed and the residue was purified using flash column chromatography with ethyl acetate and ethanol. The residue then was redissolved in methane chloride and filtred into a round bottom flask, dried using a rotary evaporation and dried in vacuum oven at 80 ℃, yielding 2.62g (82% yield) as a white crystal fine solid.

Synthesis of 1,4-dibromo-2,3,5,6-tetramethylbenzene (precursor of 15)[2,3]

1,2,4,5-tetramethylbenzene (12.5 g, 0.09 mol) was dissolved in 60 mL dichloromethane. To the stirred solution was added I_2 (0.5g, 1.97 mmol) followed by a slow dropwise addition of a solution of Br_2 (12 mL, 37.3g, 0.24 mol) in 50 mL of dichloromethane. After the addition was complete, the resulting solution was refluxed for1h. Upon cooling, 5M $NaOH_{aq}$ (25 mL) was added to the reaction mixture. The product was collected by filtration, washed with H_2O and acetone, and recrystallized from dichloromethane to afford 1,4-dibromo-2,3,5,6-tetramethylbenzene (13 g, 50%) as a colorless needle-shaped crystals. ^{1}H NMR (400 MHz, CD_2Cl_2, ppm) δ: 2.56-2.47 (s, 12H). ^{13}C NMR (125 MHz, CD_2Cl_2, ppm) δ: 135.02, 128.13, 22.28. ^{1}H and ^{13}C NMR spectra are shown below.

Synthesis of 1,4-dialdehyde-2,3,5,6-tetramethylbenzene (15)[4]

1,4-Dibromo-2,3,5,6-tetramethylbenzene (1.168g, 4.0 mmol) was dissolved in 25 mL distilled THF then cooled down to -78 ˚C. Under an Ar atmosphere, 3.2 mL of 2.5M n-BuLi (8 mmol) in hexane was added and stirred for 30 minutes. To the solution was added 0.3 mL DMF (0.30g, 4 mmol). The solution was stirred for a further 30 minutes before adding 6.4 mL 2.5 M n-BuLi slowly, the solution was stirred a further 2 h, 0.9 mL of DMF was added and the solution allowed to warm to room temperature. 20 mL 2M HCl was added to the mixture followed by 50 mL diethyl ether. The organic phase was separated and the aqueous phase washed twice with 20 mL diethyl ether. The organic extracts were combined, washed with water, and dried over $MgSO_4$, filtered and removed in vacuo. The white residue was recrystallized from petroleum ether to give the colorless needle-shaped crystal (0.37g, 49%). ^{1}H NMR (400 MHz, CD_3Cl, ppm) δ: 2.56-2.47 (s, 12H). ^{13}C NMR (125 MHz, CD_3Cl, ppm) δ: 135.02, 128.13, 22.28. ^{1}H and ^{13}C NMR spectra are shown below. HRMS (m/z): $[M]^+$ calcd for $C_{12}H_{15}O_2^+$, 191.1072; found, 191.1059.

Synthesis of 2,2'-(2,3,5,6-tetramethyl-2-yl)bis(4,5-diphenyl-imidazole) (precursor to model compounds)

In an 100 mL round-bottom flask, 1,4-dialdehyde-2,3,5,6-tetramethylbenzene (0.38g, 2.0 mmol), benzil (0.92 g, 4.4 mmol), and ammonium acetate (3.0g, 40 mmol) were added with 10 mL acteic acid and 40 mL ethanol. The reaction mixture was heated to reflux for 18 h. The reaction was then cooled to room temperature and poured into water. The precipitate was recrystallized from acetonitrile to yield 6 (0.91 g, 80%) as white crystals. ^{1}H NMR (400 MHz, DMSO-*d*6, ppm) δ: 12.51-12.33 (s, 2H), 7.65-7.14 (m, 20H), 2.14-2.05 (s, 12H). ^{13}C NMR (125 MHz, DMSO-*d*6, ppm) δ: 146.49, 136.52, 136.03, 134.29, 133.63, 133.49, 131.81, 129.16, 128.62, 128.39, 127.87, 127.61, 127.03. ^{1}H and ^{13}C NMR spectra are shown below. HRMS (m/z): $[M]^+$ calcd for $C_{40}H_{35}N_4^+$, 571.2862; found, 571.2851.

Synthesis of 2,2'-(2,3,5,6-tetramethyl-2-yl)bis(4-chlorophenyl-5-diphenyl-imidazole) (17)

In an 100 mL round-bottom flask, 1,4-dialdehyde-2,3,5,6-tetramethylbenzene (0.57g, 3.0 mmol), chlorobenzil (1.61 g, 6.6 mmol), and ammonium acetate (4.6 g, 60 mmol) were added with 15 mL acteic acid and 60 mL ethanol. The reaction mixture was heated to reflux for 18 h. The reaction was then cooled to room temperature and poured into water. The precipitate was recrystallized from acetonitrile/water to yield **6** (1.50 g, 78%) as white crystals. ^{1}H NMR (400 MHz, DMSO-d6, ppm) δ: 12.51-12.33 (s, 2H), 7.65-7.14 (m, 20H), 2.14-2.05 (s, 12H). ^{13}C NMR (125 MHz, DMSO-d6, ppm) δ:. ^{1}H and ^{13}C NMR spectra are shown below. HRMS (m/z): $[M]^+$ calcd for $C_{40}H_{33}Cl_2N_4^+$, 639.2082; found, 639.2073.

Synthesis of model compounds 10-14

Compounds **10**, 2,2'-(2,3,5,6-tetramethylbenzene-1,4-yl)bis(4,5-diphenyl-imidazole) (0.285g, 0.5 mmol) was dissloved in 10 mL DMSO. 0.4 mL 5M KOH was added following with iodomethane (100 μl, 1.6 mmol). The mixture was stirred for 30 minutes. Then produce was precipated in 50 mL water containing 2.0 g KOH, filtered, dissloved in DCM, washed with water, brine and dried over $MgSO_4$. The product was filtered, and 1.0 mL iodomethane was added and reacted in a sealed flask at 80 ˚C over night. **10** was recrystallized from ethanol/water (80/20 mL) to yield 0.38g (86%). ^{1}H NMR (400 MHz, methanol-*d*4, ppm) δ: 7.63-7.45 (m, 20H), 3.70-3.58 (s, 12H), 2.38-2.52 (s, 12H).

^{13}C NMR (125 MHz, methanol-*d*4, ppm) δ: 144.01, 137.71, 132.85, 130.83, 130.28, 128.87, 126.11, 125.05, 33.38, 16.59. ^{1}H and ^{13}C NMR spectra are shown below. HRMS (m/z): $[M]^{2+}$ calcd for $C_{44}H_{44}N_4^{2+}$, 314.6822; found, 314.6795. Compounds **11-14** were synthesized following the similar procedure, instead, the second step alkylation of them are finished in acetonitrile. Compound **11** was recrystallized to 82% yield. ^{1}H NMR (400 MHz, CD_2Cl_2, ppm) δ: 7.58-7.36 (m, 20H), 4.23-4.09 (m, 4H), 3.83-3.72 (d, 6H), 2.44-2.29 (s, 12H), 1.48-1.32 (m, 4H), 1.19-1.04 (m, 4H), 0.67-0.53 (m, 6H). ^{13}C NMR (125 MHz, CD_2Cl_2, ppm) δ: 144.12, 137.84, 133.10, 132.33, 130.90, 130.85, 130.82, 130.77, 130.71, 130.57, 129.40, 129.21, 126.17, 124.94, 124.51,47.45, 36.16, 36.01, 31.40, 31.37, 19.34, 19.21, 12.97. The ^{1}H and ^{13}C NMR spectra are shown below. HRMS (m/z): $[M]^{2+}$ calcd for $C_{50}H_{56}N_4^{2+}$, 356.2253; found, 356.2248. Compound **12** was recrystallized to 88% yield. ^{1}H NMR (400 MHz, DMSO-*d*6, ppm) δ: 7.73-7.48 (m, 20H), 4.02-3.82 (t, 8H), 2.33-2.20 (s, 12H), 1.04-0.93 (t, 12H). ^{1}H NMR spectra are shown below. HRMS (m/z): $[M]^{2+}$ calcd for $C_{48}H_{52}N_4^{2+}$, 342.2096; found, 342.2098. Compound **13** was recrystallized to 83% yield. ^{1}H NMR (400 MHz, DMSO-*d*6, ppm) δ: 7.69-7.47 (m, 20H), 3.96-3.77 (t, 8H), 2.38-2.18 (s, 12H), 1.42-1.22 (m, 8H), 0.65-0.47 (t, 12H). ^{13}C NMR (125 MHz, CD_2Cl_2, ppm) δ: 171.56, 142.85, 137.78, 132.57, 131.59, 129.42, 125.86, 22.36, 18.43, 11.21. ^{1}H NMR spectra are shown below. HRMS (m/z): $[M]^{2+}$ calcd for $C_{52}H60N_4^{2+}$, 370.2409; found, 370.2414. Compound **14** was recrystallizedto 81% yield. ^{1}H NMR (400 MHz, DMSO-*d*6, ppm) δ: 7.68-7.47 (m, 20H), 3.95-3.80 (t, 8H), 2.35-2.23 (s, 12H), 1.33-1.19 (m, 8H), 1.02-0.88 (m, 8H), 0.54-0.44 (t, 12H). ^{1}H NMR spectra are shown below. HRMS (m/z): $[M]^{2+}$ calcd for $C_{56}H_{68}N_4^{2+}$, 398.2722; found, 398.2714.

Monoalkylation of 2,2'-(2,3,5,6-tetramethyl-2-yl)bis(4-chlorophenyl-5-diphenyl-imidazole)

Mono-methylated monomer, **18.** In an 100 mL round-bottom flask, 2,2'-(2,3,5,6-tetramethyl)bis(4-chlorophenyl-5-diphenyl-imidazole) (0.64 g, 1.0 mmol) was dissloved in 20 mL DMSO. 5M KOH (0.8 mL, 4.0 mmol) was added. The solution changed colour from initial dark green to light yellow within 10 min. After 30 minutes, iodomethane (0.2 mL, 3.2 mmol) was added and after 30 minutes, the solution was poured into 400 mL of water containing 4.0 g KOH. The precipatate was filtered, washed with water, and with acetonitrile to yield **18** (1.17 g, 93%) as a white powder. ^{1}H NMR (400 MHz, CD_2Cl_2, ppm) δ: 7.61-7.10 (m, 18H), 3.23-3.11 (d, 6H), 2.17-2.01 (s, 12H). ^{13}C NMR (125 MHz, CD_2Cl_2, ppm) δ: 135.73, 135.13, 133.92, 132.33, 132.27, 131. 41, 131.19, 130.83, 130.08, 129.27, 129.10, 129.05, 128.69, 128.06, 127.74, 126.58, 126.19. ^{1}H and ^{13}C NMR spectra are shown below. HRMS (m/z): $[M]^{+}$ calcd for $C_{40}H_{33}Cl_2N_4^{+}$, 667.2395; found, 667.2380. Compounds **19-21** were synthesized following a similar procedure. Compound **19**was prepared in 91% yield. ^{1}H NMR (400 MHz, CD_2Cl_2, ppm) δ: 7.64-7.12 (m, 18H), 3.69-3.55 (m, 4H), 2.22-2.10 (s, 12H), 1.00-0.86 (m, 6H). The ^{1}H NMR spectra are shown below. HRMS (m/z): $[M]^{+}$ calcd for $C_{44}H_{41}Cl_2N_4^{+}$, 695.2708; found, 695.2675. Compound **20** was prepared in 94% yield. ^{1}H NMR (400 MHz, CD_2Cl_2, ppm) δ: 7.61-7.15 (m, 18H), 3.61-3.42 (m, 4H), 2.25-2.10 (s, 12H), 1.43-1.24 (m, 4H), 0.71-0.52 (m, 6H). ^{1}H NMR spectra are shown below. HRMS (m/z): $[M]^{+}$ calcd for $C_{46}H_{45}Cl_2N_4^{+}$, 723.3021; found, 723.2989. Compound **21** was prepared in 89% yield. ^{1}H NMR (400 MHz, CD_2Cl_2, ppm) δ: 7.63-7.13 (m, 18H), 3.67-3.44 (m, 4H), 2.24-2.07 (s, 12H), 1.37-1.19 (m, 4H), 1.09-0.88 (m, 4H), 0.73-0.52 (m, 6H).). ^{13}C NMR (125 MHz, CD_2Cl_2, ppm) δ: 195.21, 194.28, 185.70, 171.61, 164.43, 146.86, 146.67, 135.92, 135.10, 134.98, 132.41, 131.10, 129.29, 129.10, 128.70,

128.03, 127.76, 127.66, 126.59, 43.74, 19.60, 19.49, 17.39, 13.99, 13.06, 12.94, 12.90. ^{1}H and ^{13}C NMR spectra are shown below. HRMS (m/z): $[M]^+$ calcd for $C_{48}H_{49}Cl_2N_4^+$, 751.3334; found, 751.3314.

Synthesis of PAImXY(#)

PAImMM(**10**), 2,2'-bipyridine (0.075 g, 0.48 mmol) was placed in a 50 mL round-bottom flask. Using a needle through the septum, the flask was evacuated and refilled with argon three times. $Ni(COD)_2$ (0.132 g, 0.48 mmol) was transferred into the flask. The flask was evacuated and purged with argon three times. Anhydrous DMF (5 mL) was added and the mixture heated to 80˚C for 30 minutes. In a separate flask, 2,2'-(2,3,5,6-tetramethyl-2-yl)bis(3-methyl-4-chlorophenyl-5-diphenyl-imidazole) (0.1335 g, 0.2 mmol) and 5 mL anhydrous DMF was added. The flask was purged with argon and after the catalyst heated for 30 minutes before transferring the monomer solution into catalyst solution. The solution was heated at 80 °C while stirring for 20 h. After cooling, the solution was poured into 200 mL 6 M HCl, to consume the catalyst. And the solid was filtered and washed with water, aqueous sodium bicarbonate and acetone. After drying in vacuo, the solid was dissolved in 5 mL DCM and 5 mL DMSO. 20 times equilvent MeI was added and the solution heated to 80 °C for three days. The polymer was precipated into 100 mL ethyl acetate washed with acetone and filtered to get brown solid 0.1695 g (100 % yield). The other polymers were prepared following the same way which PAImMB(**11**) was prepared in 100% yield, PAImEE(**12**) was prepared in 100% yield, PAImPP(**13**) was prepared in 99% yield, PAImBB(**14**) was prepared in 98% yield.

Thermal stability. The thermal stability of the membranes was measured by thermogravimetric analysis (TGA) using a SHIMADZU thermogravimetric analyzer apparatus under nitrogen atmosphere at a heating rate of 5 ˚C/minbetween the temperature range 25 to 800 ˚C.

Supplementary References

1 Fan, J. *et al.* Cationic Polyelectrolytes, Stable in 10 M KOHaq at 100 °C. *ACS Macro Letters* **6**, 1089-1093 (2017).

2 Gu, C. *et al.* Porous Organic Polymer Films with Tunable Work Functions and Selective Hole and Electron Flows for Energy Conversions. *Angew. Chem. Int. Ed.* **55**, 3049-3053 (2016).

3 Liu, Y., Xu, X., Zheng, F. & Cui, Y. Chiral octupolar metal-organoboron NLO frameworks with (14,3) topology. *Angew. Chem. Int. Ed.* **47**, 4538-4541 (2008).

4 Kuhnert, N., Rossignolo, G. M. & Lopez-Periago, A. The synthesis of trianglimines: on the scope and limitations of the[3-3] cyclocondensation reaction between (1R,2R)-diaminocyclohexane and aromatic dicarboxaldehydes. *Org. Biomol. Chem.* **1**, 1157-1170 (2003).
